# Nitrogen‐Doped Mesoporous Carbon Loaded With Zn Single Atoms as a Catalyst for the Synthesis of 2,4(5)‐Diarylimidazoles

**DOI:** 10.1002/open.70318

**Published:** 2026-10-13

**Authors:** Maryam Shojaei‐Asl, Mohsen Moradian

**Affiliations:** ^1^ Department of Organic Chemistry Faculty of Chemistry University of Kashan Kashan Iran

**Keywords:** 2,4(5)‐diarylimidazole synthesis, green chemistry, nitrogen‐doped mesoporous carbon, one‐pot Radziszewski reaction, zinc single‐atom catalyst

## Abstract

Single‐atom catalysts have emerged as highly effective alternatives to conventional metal catalysts due to their uniform active sites and superior catalytic performance. Stabilizing single zinc atoms on nitrogen‐doped mesoporous carbon (NMC) enhances their stability, selectivity, electrical conductivity, and compatibility with green chemistry principles. In this study, a Zn@NMC catalyst was synthesized via solvent evaporation‐induced self‐assembly, followed by thermal polymerization and pyrolysis, and characterized by ^1^H nuclear magnetic resonance (NMR), Brunauer–Emmett–Teller, thermogravimetric analysis, scanning electron microscopy, and energy‐dispersive X‐ray mapping analyses. The catalyst was successfully applied in the one‐pot Radziszewski synthesis of 12 2,4(5)‐diarylimidazole derivatives from phenylglyoxal, various substituted benzaldehydes, and ammonium acetate. ^1^H NMR, ^13^C NMR, and Fourier transform infrared spectroscopy were used to characterize the products. Optimal results were obtained in methanol at room temperature (25 °C) for 12 h, affording the desired products in yields of up to 97%. The incorporation of isolated zinc atoms into the nitrogen‐rich carbon matrix significantly improved selectivity toward the target diarylimidazoles by suppressing side reactions and preventing catalyst deactivation.

## Graphical abstract

A zinc single‐atom catalyst anchored on nitrogen‐doped mesoporous carbon (Zn@NMC) was synthesized via an evaporation‐induced self‐assembly method and applied in the one‐pot synthesis of 2,4(5)‐diarylimidazoles. The catalyst was fully characterized using FT‐IR, XRD, SEM, EDX, BET, and TGA analyses. Zn@NMC enabled efficient imidazole synthesis at room temperature in methanol, requiring only 7 mg catalyst and achieving excellent yields (75%–97%). The nitrogen‐rich carbon matrix stabilized atomic Zn sites, enhancing selectivity and preventing deactivation. The catalyst exhibited remarkable recyclability and stability over multiple cycles. This green, low‐energy method provides a practical and sustainable route for heterocyclic drug‐related scaffolds.

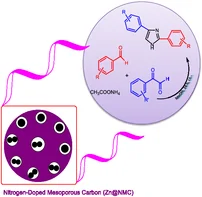

## 1 Introduction

The imidazole ring structure is key structure to many natural substances and biologically active pharmaceutical agents. The biological activities of the imidazole ring system include antimicrobial [1], antiviral [2], antitubercular [3], anticancer [4], anti‐inflammatory [2], antibacterial [5], antiparasitic [6], antifungal [7], anti‐obesity [8], antihypertensive [9], and various enzyme‐inhibiting activities [3, 5]. The amino acid histidine, the sedative etomidate [10], the anti‐ulcer drug cimetidine [11], the proton pump inhibitor omeprazole [12], the antifungal agent ketoconazole [13], and the benzodiazepine antidote flumazenil [14] are examples of imidazole derivatives. Due to the wide range of uses of imidazole derivatives in the development of therapeutic agents, there is an ongoing demand for straightforward and effective strategies for synthesizing imidazole and related compounds. Many imidazole derivatives are widely documented in scholarly literature. However, there remains a need to establish a pharmacophore with an imidazole core capable of demonstrating diverse biological effects. Japp and Radziszewski developed a pioneering method for creating an imidazole core in 1882. Their approach involved combining 1,2‐dicarbonyl compounds with aldehydes and ammonia to produce 2,4,5‐triphenylimidazoles [15, 16, 17]. As a result, a variety of methods for making this crucial heterocyclic compound have been documented in the academic literature. For instance, 2,4‐diaryl‐*1H*‐imidazoles are frequently synthesized using amidines and α‐bromoarylketones [18]. Zhang and Chen established a method for synthesizing asymmetric 2,4‐diarylimidazoles lacking C5 substitution. In this process, acetophenones undergo in situ transformation into α‐tosyloxyacetophenones, which then react with arylamidines to yield the desired target compounds [19]. Ueno and Togo outlined a two‐step method for synthesizing imidazoles by reacting polymer‐bound [hydroxy(sulfonyloxy)iodo] benzene with ketones or alcohols, followed by treatment with benzamidine [20]. The Suzuki reaction is comparably beneficial for the synthesis of 2,4‐diarylimidazoles, as indicated by the study conducted by Langhammer and Erker [21]. Bellina et al. described a three‐step synthetic approach for creating 2,4(5)‐diaryl‐*1H‐*imidazoles, using 1‐benzyl‐*1H*‐imidazole as the starting material. This methodology encompasses the C‐5 arylation of 1‐benzyl‐*1H*‐imidazole using aryl halides in the absence of a base catalyst, and the resulting yields of 2,4(5)‐diaryl‐*1H*‐imidazoles were deemed satisfactory [22]. Abdel‐Wahab et al. synthesized 2,4(5)‐diarylimidazoles from aldehydes, amines, and nitriles or carboxylic acids. The resulting compounds showed anti‐inflammatory characteristics by inhibiting cyclooxygenase (COX‐2) activity [23]. The synthesis of diarylimidazoles often faces significant challenges such as low yields, lack of selectivity, and the formation of undesired by‐products, necessitating the use of catalysts. Conventional methods for imidazole synthesis via condensation reactions are often inefficient, yielding low product quantities and offering a restricted range of substrates, particularly when incorporating various aryl groups or targeting regioselectivity [24]. Metal‐based catalysts, such as palladium, copper, and iodine, are utilized to accelerate reaction rates, boost yields, and selectively generate the desired diarylimidazole isomers by assisting crucial bond‐forming processes such as C–N or C–C coupling [24, 25, 26]. In contemporary research, single‐atom catalysts (SACs) with uniformly distributed active sites have emerged as powerful tools for enhancing the catalytic efficiency and selectivity in diarylimidazole synthesis [27, 28, 29]. Unlike conventional heterogeneous catalysts, SACs exhibit improved catalytic efficiency and tunable reactivity. This is due to the isolated metal atoms that minimize secondary reactions and facilitate the production of desired compounds. The integration of homogeneous and heterogeneous catalysis by SACs enables scalable, stable, and cost‐effective methods, making them highly advantageous for precise chemical synthesis and a range of industrial applications [27,29, 30, 31]. Due to their large surface area and adaptable, interconnected pore structures, mesoporous carbon (MC) materials serve as excellent substrates for SACs. These characteristics enhance the even distribution and stabilization of single‐metal atoms, thereby minimizing clustering and optimizing atomic efficiency [32, 33]. MCs, featuring pore sizes ranging from 2 to 50 nm, are widely used in adsorption, catalysis, and mass transport due to their high surface area, ample pore volume, thermal stability, and electrical conductivity [34]. Nonetheless, their surfaces are often passive, resulting in insufficient hydrophilicity, which limits their broad application [35]. Nitrogen‐doped mesoporous carbons (NCMs) address these issues and have been developed as recyclable heterogeneous base catalysts, in accordance with green chemistry principles [36, 37, 38, 39]. The incorporation of nitrogen into the doping process facilitates the introduction of polar nitrogen functional groups onto mesoporous carbon, thereby augmenting its surface polarity and facilitating more robust interactions with solvent molecules, in addition to improving its electrical conductivity [40, 41, 42, 43]. NCMs synthesized by the calcination and ammoxidation of polyacrylonitrile and N‐carbons have shown efficiency in reactions such as the Knoevenagel condensation of benzaldehyde with ethyl cyanoacetate [44, 45]. Researchers have employed nitrogen‐doped carbons and similar materials for various purposes such as energy storage [40, 46, 47], organic synthesis [48], and catalysis of hydrogen evolution [49]. Numerous studies have been published on the application of SACs in the synthesis and modification of diverse organic compounds. For example, Cheong et al. [50] found that a single‐atom Fe catalyst, which was isolated and supported on mesoporous nitrogen‐doped carbon (Fe/N‐C), demonstrated outstanding performance and compatibility with various functional groups in the transfer hydrogenation of nitroarenes. This is attributed to the atomically distributed Fe sites and decreased energy barriers, which resulted in high selectivity [50]. Gaikwad et al. [51] illustrated that Cu SACs anchored on mesoporous graphitic carbon nitride (Cu@mpg‐C_3_N_4_) effectively facilitated the visible‐light‐driven photocatalytic oxidative dehydrogenation of N‐heterocyclic amines into N‐heteroaromatics, achieving superior conversion and selectivity (>99%) in an aqueous environment, underscoring their promise as sustainable and green catalysts [51]. An evaluation of existing research reveals that, thus far, SACs on NMC frameworks have not been used to produce arylimidazoles. In this study, a simple one‐pot solution‐phase synthesis method was developed to prepare imidazole derivatives with functional groups at C2 and C4 and a fixed C5, called 2,4(5)‐diarylimidazoles, as NPY5 receptor antagonists. Zn@*N*‐doped mesoporous carbon (Zn@NMC) was synthesized and used as the catalyst. The optimal conditions for the synthesis of 2,4(5)‐diarylimidazoles were determined. Using a low catalyst amount under ambient conditions aligns with green chemistry, lowering energy consumption and making it suitable for multicomponent reactions in synthesizing pharmaceutical compounds.

## 2 Materials and Methods

### 2.1 Preparation of the Catalyst

NMC containing a single zinc atom (Zn@NMC) was synthesized using a controlled method of evaporation‐induced self‐assembly (EISA). This methodology encompassed an innovative nitrogen‐doped carbon precursor, which was subjected to thermopolymerization and pyrolysis processes. This research involved three main phases in the catalyst synthesis process: ligand preparation, metal incorporation, and polymerization, as shown in Scheme 1. A portion of the synthetic procedures was conducted in accordance with the established reference [52]. The ligand was first prepared through the reaction of allyl esters with ethylenediamine. A flask was charged with a mixture containing ethylenediamine (1 mmol) and allyl ester (2 mmol), and then methanol (20 mmol) was introduced. The solution was then refluxed at 100 °C for 2 h. Following this interval, the reflux apparatus was removed, and the contents of the flask were transferred to ice to facilitate precipitate formation. The resultant precipitate was filtered and dried. Subsequently, an array of metallic compounds, including nickel acetate and zinc acetate, was introduced to the ligand. The ligand (1 mmol) and the related acetate salt (1 mmol) were dissolved in methanol (10 mmol) and then slowly added to the ligand. The resultant mixture was subjected to reflux at 80 °C for 6 h. Subsequently, the materials contained within the flask were subjected to refrigeration for duration of 24 h to facilitate the process of precipitation, which was subsequently filtered and dried in preparation for further applications. In the concluding phase, the polymerization process was executed through the incorporation of benzoyl peroxide into the dried metal precipitate within a methanol solvent for 4 h at 80 °C.

**SCHEME 1:**
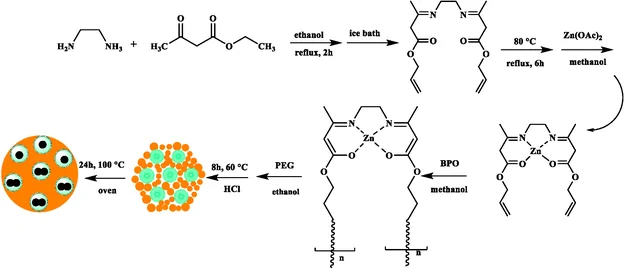
Synthesis of the bidentate ligand coordinated to the Zn single atom.

Subsequently, polyethylene glycol was added and combined with the polymerized ligand in a mixture of ethanol and water, which was then placed in an oil bath for 8 h at 60 °C. After completing this stage, the obtained precipitate was dried, and the material was subjected to a nitrogen gas atmosphere in a furnace to form a mesoporous carbon structure. Scheme 2 showcases the method of synthesizing Zn@NMC using mesoporous carbon (MC) along with the freshly prepared ligand.

**SCHEME 2:**
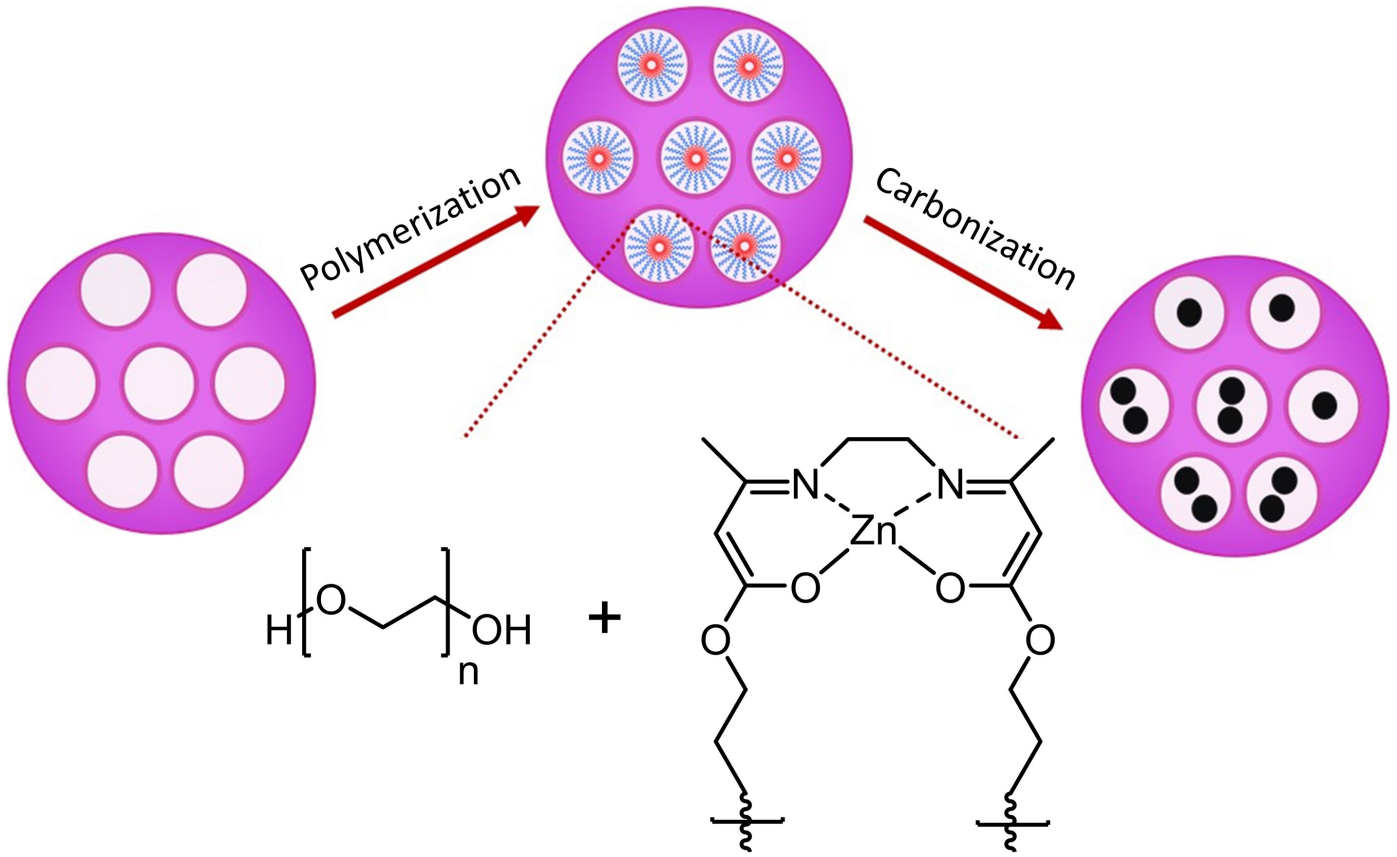
Synthesis of Zn@NMC from mesoporous carbon (MC) and the synthesized ligand.

### 2.2 Synthesis of Aryl Glyoxal

Twelve mL of dioxane was added to a round‐bottomed flask. Subsequently, 10 mmol of selenium dioxide and 1 mL of distilled water were added to the flask. The temperature of the reaction mixture was slowly raised to 55 °C to improve the dissolution of selenium dioxide. Following this, 10 mmol of acetophenone was added to the reaction mixture, and the reaction was carried out under reflux at 100 °C for 7 h. After the reaction was completed, the solution was carefully processed to separate the solid precipitate from the liquid component. Afterward, distilled water was introduced to the remaining solution, and the resulting blend was heated to 100 °C with the aid of a mechanical stirrer for a designated period to achieve the desired concentration. To facilitate the crystallization process, the solution was stored in a refrigerated environment. Ultimately, the precipitate was collected through filtration and then rinsed with distilled water to ensure its purity.

### 2.3 General Procedure for the Synthesis of 2,4(5)‐Diarylimidazoles

Over 10 min, a mixture of an aryl aldehyde solution (0.70 mmol), ammonium acetate (3.41 mmol), and Zn@NMC catalyst dispersed in 3.5 mL of methanol was gradually introduced into a methanol solution (3.8 mL) of phenylglyoxal monohydrate (0.70 mmol). The mixture was subjected to continuous agitation for an extended period overnight at room temperature, and the solvent was eliminated through evaporation. The residual material was then distributed in equal parts (20 mL) of a saturated sodium bicarbonate solution in water and CH_2_Cl_2_. The organic layer was dried using sodium sulfate (Na_2_SO_4_), and the solvent was eliminated through vacuum evaporation. The crude reaction mixture was subjected to targeted extraction using a strong cation exchanger column (2 g, 30–90 µm, capacity 0.4 mequiv/g). This approach enabled the selective separation of the nonbasic compound, 2‐benzoyl‐4(5)‐phenylimidazole, from the basic compound 2,4(5)‐diarylimidazole. The column was initially washed with a 10 mL solution made by mixing dichloromethane and methanol in equal volumes. Subsequently, 10 mL of methanol was utilized to extract the nonbasic products. The target compound, 2,4(5)‐diarylimidazoles, was isolated using a 10 mL methanol solution that contained 5% ammonia. A 5% (w/w) hydrochloric acid solution in ethanol was employed to transform the free base into its hydrochloride salt. The resulting product was crystallized using a combination of absolute ethanol and diethyl ether.

### 2.4 Spectroscopic and Physical Data

The Fourier transform infrared (FT‐IR) and nuclear magnetic resonance (NMR) spectra of all synthesized compounds are included in the Supporting Information (Figures S1–S37).

*2*,*4*(*5*)*‐Diphenylimidazole* (***3a***); White solid; Yield 85%; m.p: 275–277 °C (Lit. m.p: 274–275 °C) [53]; FT‐IR (KBr): ν (cm^−1^) = 3407 (N–H), 3058–2850 (C–H), 1605 (C═N), 1582–1490 (C═C); ^1^H NMR (400 MHz, DMSO‐*d*_6_); *δ* (ppm) = 12.76 (s, NH,1H), 7.97 (d, *J *= 8.4 Hz, 2H, CH‐Ar), 7.85 (d, *J  *= 7.2 Hz, 2H, CH), 7.78 (s,1H, CH‐Im), 7.68 (m, 3H, CH‐Ar), 7.39 (t, *J *= 7.6 Hz, 2H, CH), 7.23 (t, *J *= 7.2 Hz, 1H, CH) ppm; ^13^C NMR (100 MHZ, DMSO‐*d*_6_); *δ* (ppm) =  143.8, 141.3, 135, 131.7, 130.7, 130.5, 128.9, 127.8, 126.6, 124.9, 115.

*2‐*(*2‐Chlorophenyl*)*‐4*(*5*)*–phenyl imidazole* (***3b***); Pale yellow solid; Yield 87%; m.p: 264–267 °C (New Compound); FT‐IR (KBr): ν (cm^−1^) = 3431 (N–H), 2976–3027 (C–H), 1603 (C═N), 1563–1455 (C═C); ^1^H NMR (400 MHz, DMSO‐*d*_6_); *δ* (ppm)  =  12.47 (s, NH, 1H), 7.85 (d, *J *= 7.2 Hz, 1H, CH‐Ar), 7.77 (s,1H, CH‐Im), 7.60 (d, *J* = 8.2 Hz, CH, 2H), 7.46 (m, 2H, CH‐Ar), 7.39 (t, *J *= 7.6 Hz, 2H, CH‐Ar), 7.23 (t, *J *= 7.2 Hz, 1H, CH) ppm;^13^C NMR (100 MHZ, DMSO‐*d*_6_); *δ* (ppm) = 143.8, 141.3, 134.9, 131.7, 131.3, 130.7, 130.5, 130.4, 128.9, 127.8, 126.8, 124.9, 115.0; Elemental Analysis, Calc: C, 70.73; H, 4.35; Cl, 13.92; N, 11.00; Found, C, 71.03; H, 4.33; Cl, 13.98; N, 10.66.

*2‐*(*4‐Chlorophenyl*)*‐4*(*5*)*–phenyl imidazole* (***3c***); White solid; Yield 87%; m.p: 260–264 °C (Lit. m.p. 263–265 °C) [54]; FT‐IR (KBr): ν (cm^−1^) = 3417 (N–H), 3030–2898 (C–H), 1601 (C═N), 1483–1449 (C═C); ^1^H NMR (400 MHz, DMSO‐*d*_6_); *δ* (ppm) = 12.78 (s, NH, 1H), 8.02 (d, *J =*  8.4 Hz, 2H, CH‐Ar), 7.87 (d, *J  *=  8.4 Hz, 2H, CH), 7.81 (s,1H, CH‐Im), 7.55 (d, *J *= 8.4 Hz, 2H, CH‐Ar), 7.38 (t, *J *= 7.6 Hz, 2H, CH), 7.22 (t, *J *= 7.2 Hz, 1H, CH) ppm; ^13^C NMR (100 MHZ, DMSO‐*d*_6_); *δ* (ppm) = 145.7, 141.1, 134.9, 133.6, 129.9, 129.3, 128.9, 127, 126.8, 124.1, 115.2.

*2‐*(*4‐Methylphenyl*)*‐4*(*5*)*–phenyl imidazole* (***3d***); White solid; Yield 90%; m.p: 258–262 °C (New Compound); FT‐IR (KBr): ν (cm^−1^) = 3410 (NH), 3030–2915 (C–H), 1602 (C═N), 1494–1451 (C═C); ^1^H NMR (400 MHz, DMSO‐*d*_6_); *δ* (ppm)  =  12.85 (s, 1H, NH), 7.92 (d, 2H, *J =* 8.4 Hz, CH‐Ar), 7.85 (d, *J *= 8.2 Hz, 2H, CH‐Ar), 7.70 (s, 1H, CH‐Im), 7.38 (t, *J *= 8 Hz, 2H, CH), 7.28 (d, *J *= 8.4 Hz, 2H, CH), 7.22 (t, *J* = 7.6 Hz, 1H, CH) ppm; ^13^C NMR (100 MHZ, DMSO‐*d*_6_); *δ* (ppm) = 147.3, 140.8, 138.2, 134.5, 131.8, 130.3, 128.2, 126.8, 125.4, 119.3, 115.1, 21.34; Elemental Analysis, Calc: C, 82.02; H, 6.02; N, 11.96; Found, C, 82.06; H, 5.99; N, 11.95.

*2‐*(*4‐Boromophenyl*)*‐4*(*5*)*–phenyl imidazole* (***3e***); Yellow solid; Yield 94%; m.p: 265–266 °C (New Compound); FT‐IR (KBr): ν (cm^−1^) = 3421 (N–H), 3073–2930 (C–H), 1600 (C═N), 1557–1481 (C═C); ^1^H NMR (400 MHz, DMSO‐*d*_6_); *δ* (ppm)  =  12.77 (s, 1H, NH), 7.96 (d, *J  *= 8.4 Hz, 2H, CH‐Ar), 7.87 (d, *J* = 8 Hz, 2H, CH‐Ar), 7.81 (s, 1H, CH‐Im), 7.69 (d, *J* = 8.8 Hz, 2H, CH), 7.39 (t, *J* = 7.6 Hz, 2H, CH), 7.23 (t, *J* = 7.2 Hz, 1H, CH) ppm;^13^C NMR (100 MHZ, DMSO‐*d*_6_); *δ* (ppm) = 145.2, 141.7, 134.9, 133.1, 129.9, 129.3, 128.9,127, 126.8, 124.9, 115.2; Elemental Analysis, Calc: C, 60.22; H, 3.71; Br, 26.71; N, 9.36; Found: C, 60.52; H, 3.51; Br, 26.91; N, 9.40.

*2‐*(*3‐Boromophenyl*)*‐4*(*5*)*–phenyl imidazole* (***3f***); Pale yellow solid; Yield 76%; m.p: 270–272 °C (New Compound); FT‐IR (KBr): ν (cm^−1^) = 3402 (NH), 3069–2951 (C–H), 1665 (C═N), 1564–1483 (C═C).^1^H NMR (400 MHz, DMSO‐*d*_6_); *δ* (ppm) = 12.76 (s, 1H, NH), 7.95 (d, *J* = 8 Hz, 2H, CH‐Ar), 7.85 (d, *J* = 7.6 Hz, 2H, CH‐Ar), 7.81 (s, 1H, CH‐Im), 7.68 (m, 2H, CH), 7.38 (t, *J *= 8.4 Hz, 2H, CH), 7.22 (t, *J *=  7.6 Hz, 2H, CH) ppm.^13^C NMR (100 MHZ, DMSO‐*d*_6_); *δ* (ppm) = 144.7, 141.8, 134.9, 133.2, 131.5, 131.2, 128.9, 127.7, 126.9, 124.9, 124.2, 122.7, 115.4; Elemental Analysis, Calc: C, 60.22; H, 3.71; Br, 26.71; N, 9.36; Found: C, 59.92; H, 3.91; Br, 26.71; N, 9.46.

*2‐Phenyl‐4*(*5*)*‐*(*4‐bromophenyl*) *imidazole* (***3g***); White solid; Yield 93%; m.p: 198–210 °C (New Compound); FT‐IR (KBr): ν (cm^−1^) = 3403 (NH), 3057–2847 (C–H), 1605 (C═N), 1460–489 (C═C); ^1^H NMR (400 MHz, DMSO‐*d*_6_); *δ* (ppm) = 8.01 (d, *J =* 8.4 Hz, 2H, CH), 7.82 (d, *J =*  8 Hz, 2H, CH), 7.80 (s, 1H, CH‐Im), 7.57 (d, *J =* 7.6 Hz, 2H, CH‐Ar), 7.47 (t, *J =* 7.2 Hz, 2H, CH‐Ar), 7.37 (t, *J =* 7.2 Hz, 2H, CH‐Ar) ppm; ^13^C NMR (CDCl3); *δ* (ppm) = 144.7, 141.8, 134.9, 133.2, 131.5, 128.9, 127.6, 126.9, 124.9, 124.2, 122.7, 115.4; Elemental Analysis, Calc: C, 60.22; H, 3.71; Br, 26.71; N, 9.36; Found: C, 60.57; H, 3.73; Br, 26.41; N, 9.29.

*2‐*(*4‐Methylphenyl*)*‐4*(*5*)*‐*(*4‐boromophenyl*) *imidazole* (***3h***); White solid; Yield 90%; m.p: 204–210 °C (New Compound); FT‐IR (KBr): ν (cm^−1^) = 3434 (NH), 3143–2915 (C–H), 1597 (C═N), 1489–1428 (C═C); ^1^H NMR (400 MHz, DMSO‐*d*_6_); *δ* (ppm) = 12.65 (s, 1H, NH), 7.89 (d, *J =* 8.4 Hz, 2H, CH), 7.82 (d, *J  =*  8.4 Hz, 2H, CH),7.81 (s,1H, CH‐Im), 7.56 (d, *J =*  7.6 Hz, 2H, CH), 7.28 (d, *J =* 8.4 Hz, 2H, CH), 2.35 (s, 3H, CH_3_) ppm;^13^C NMR (CDCl3); *δ* (ppm) = 146.7, 140.2, 138.1, 134.5, 131.8, 129.8, 128.2, 126.8, 125.4, 119.3, 115.1, 20.9; Elemental Analysis, Calc: C, 61.36; H, 4.18; Br, 25.51; N, 8.94; Found: C, 61.66; H, 3.98; Br, 25.66; N, 8.7.

*2‐*(*4‐Chlorophenyl*)*‐4*(*5*)*‐*(*4‐boromophenyl*) *imidazole* (***3i***); Yellow solid; Yield 85%; m.p: 240–242 °C (New Compound); FT‐ IR (KBr): ν (cm^−1^) = 3430 (NH), 3079–2926 (C–H), 1660 (C═N), 1480–1428 (C═C); ^1^H NMR (400 MHz, DMSO‐*d*_6_); *δ* (ppm) = 12.83 (s, 1H, NH), 8.01 (d, *J =*  8.4 Hz, 1H, CH), 7.87 (s, 1H, Im), 7.82 (d,* J =* 8.4 Hz, 2H, CH), 7.55 (d, *J =* 7.6 Hz, 4H, CH‐Ar) ppm;^13^C NMR (CDCl3); *δ* (ppm) = 145.5, 140.6, 134.2, 133.2, 131.8, 129.7, 129.3, 127.0, 126.8, 119.5, 115.8; Elemental Analysis, Calc: C, 54.00; H,3.02; Br, 23.95; Cl, 10.63; N, 8.40; Found: C, 54.25; H, 2.87; Br, 24.05; Cl, 10.67; N, 8.6.

*2‐*(*4‐Boromophenyl*)*‐4*(*5*)*‐*(*4‐boromophenyl*) *imidazole* (***3j***); Pale yellow solid; Yield 88%; m.p: 250–252 °C (New Compound); FT‐IR (KBr): ν (cm^−1^) = 3430 (N–H), 3143–2919 (C–H), 1650 (C═N), 1477–1426 (C═C); ^1^H NMR (400 MHz, DMSO‐d_6_); *δ* (ppm) = 12.83 (s, 1H, NH), 7.95 (d, *J = *8.4 Hz, 2H, CH), 7.87 (s, 1H, CH‐Im), 7.81 (d, *J = *8.2 Hz, 2H, CH), 7.68 (d, *J = *7.6 Hz, 2H, CH‐Ar), 7.75 (d, *J = *7.2 Hz, 2H, CH‐Ar) ppm;^13^C NMR (CDCl3); *δ* (ppm)  =  145.5, 140.6, 134.2, 132.2, 131.8, 130.1, 127.3, 126.8, 121.8, 119.5, 115.9; Elemental Analysis, Calc: C, 46.65; H, 2.67; Br, 42.27; N, 7.41; Found: C, 47.85; H, 2.65; Br,42.4 7; N, 7.03.

*2‐*(*3‐Boromophenyl*)*‐4*(*5*)*‐*(*4‐boromophenyl*) *imidazole* (***3k***); White solid; Yield 83%; m.p: 230–235 °C (New Compound); FT‐IR (KBr): ν (cm^−1^) = 3427 (NH), 3070–2805 (C–H), 1564 (C═N), 1442–1427 (C═C); ^1^H NMR (400 MHz, DMSO‐*d*_6_); *δ* (ppm) = 12.89 (s, 1H, NH), 8.19 (s, 1H, CH), 8.00 (d, *J =*  8.4 Hz, 2H, CH‐Im), 7.90 (s,1H, CH), 7.85 (d, *J =* 8.4 Hz, 2H, CH), 7.58 (d, *J =*  8.4 Hz, 2H, CH‐Ar), 7.45 (t, *J =*  7.6 Hz, 1H, CH‐Ar) ppm;^13^C NMR (CDCl3); *δ* (ppm) =  144.9, 140.8, 134.1, 133, 131.9, 131.5, 131.3, 127.8, 126.9, 124.3, 122.7, 119.6, 116.1; Elemental Analysis, Calc: C, 47.65; H, 2.67; Br, 42.27; N,7.41; Found: C, 47.45; H, 2.68; Br, 42.07; N, 7.8.

*2‐*(*2‐Chlorophenyl*)*‐4*(*5*)*‐*(*4‐bromophenyl*) *imidazole* (***3l***); White solid; Yield 80%; m.p: 216–218 °C (New Compound); FT‐IR (KBr); ν (cm‐1) = 3423 (N–H), 3129–3082 (C–H), 1593 (C═N), 1476–1447 (C═C); ^1^H NMR (400 MHz, DMSO‐*d*_6_); *δ* (ppm) = 12.79 (s, 1H, NH), 7.93 (d, *J = *8.4 Hz, 2H, CH), 7.83 (d, *J =* 8.2 Hz, 2H, CH), 7.68 (d, *J = *7.6 Hz, 1H, CH), 7.38 (t, *J =* 7.2 Hz, 2H, CH), 7.21 (d, *J = *7.6 Hz, 1H, CH); Elemental Analysis, Calc: C, 54.00; H,3.02; Br, 23.95; Cl, 10.63; N, 8.40; Found: C, 53.75; H, 3.17; Br, 23.85; Cl, 10.58; N, 8.65.

## 3 Results and Discussion

### 3.1 Characterization of the Catalyst

#### 3.1.1 Fourier Transform Infrared Analysis

The FT‐IR spectrum of NMC reveals several unique functional groups, as shown in Figure 1. The broad peak at 3442 cm^−1^ is associated with either O–H stretching vibrations or N─H bonds, resulting from the nitrogen incorporation [55]. The absorption band detected at 1623 cm^−1^ signifies the C═C stretching in the aromatic carbon structure. In comparison, the band at 1421 cm^−1^ corresponds to the stretching vibrations of the amine C─N bonds, thus confirming the successful nitrogen doping [56]. The absorption at 1267 cm^−1^ signifies C–N stretching vibrations within the carbon structure. The peaks at 1113 and 1025 cm^−1^ are attributable to C–O stretching vibrations, where the 1113 cm^−1^ peak notably shifts following nitrogen doping compared to the undoped carbon [57]. The peaks at lower wavenumbers, specifically at 521 and 453 cm^−1^, are related to the vibrations of the carbon skeleton and suggest that the core structure remains intact even after nitrogen is incorporated [55]. The peaks at 521 and 453 cm^−1^ confirm the presence of zinc in the NMC structure [58].

**FIGURE 1:**
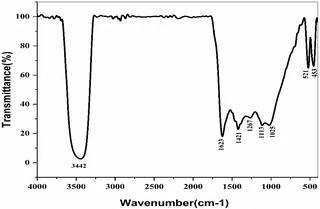
FT‐IR spectrum of Zn@NMC.

#### 3.1.2 X‐ray Diffraction Analysis

Figure 2 illustrates the X‐ray diffraction (XRD) pattern of NMC, highlighting distinct peaks that offer insights into its structural characteristics. The peak detected around 25° is associated with the (002) plane of the hexagonal graphite structure, signifying the existence of graphitic carbon layers within the material. The diffraction peak identified at approximately 32° (more accurately at 33.27°, as reported in the literature) is ascribed to the formation of crystalline domains arising from the incorporation of nitrogen into the carbon matrix. The diffraction peak observed near 45° is associated with the (100) plane of the hexagonal graphite structure [59]. The sharp peak at approximately 33° corresponds to the (100) or (002) crystal planes of ZnO with a hexagonal wurtzite structure, indicating the coordination of zinc metal with ligands through oxygen. At angles of 45°, 57°, and 67°, the peaks are linked to the (101), (102), and (103) crystallographic planes of ZnO, respectively [60].

**FIGURE 2:**
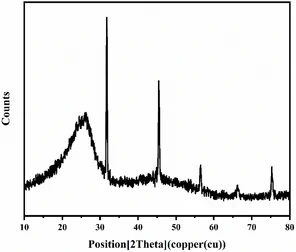
XRD spectrum of Zn@NMC.

#### 3.1.3 Energy‐Dispersive X‐ray Analysis

Figure 3 shows the energy‐dispersive X‐ray (EDX) analysis of Zn@NMC, which detected the presence of carbon (C), oxygen (O), nitrogen (N), and zinc (Zn) in the material. The weight percentages of C, N, O, and Zn were approximately 73.54%, 9.33%, 11.19%, and 5.94%, respectively. These findings substantiate the practical fabrication of NMC and the homogeneous distribution of individual Zn atoms throughout its structure.

**FIGURE 3:**
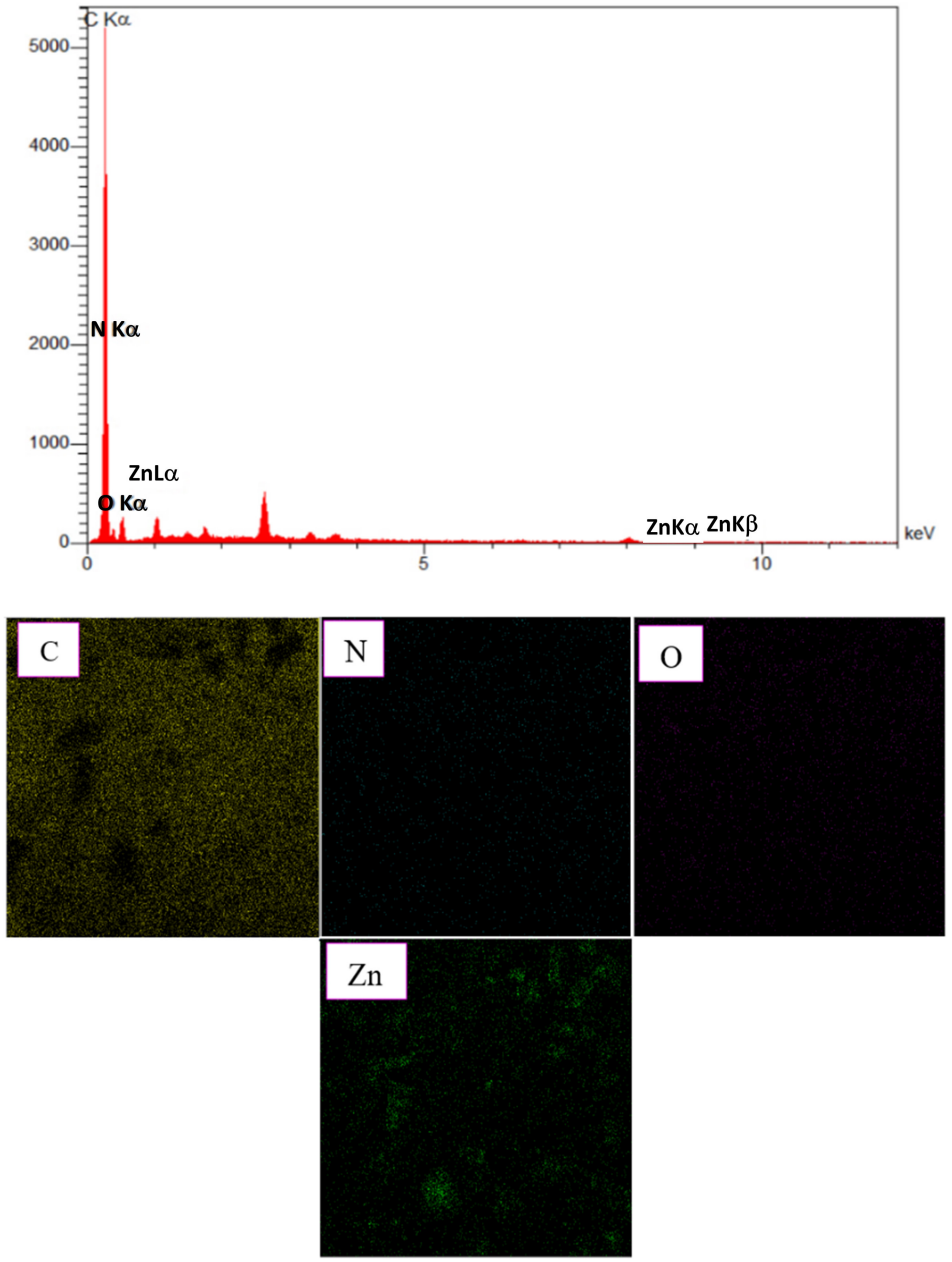
EDX and elemental composition of Zn@NMC.

#### 3.1.4 Scanning Electron Microscopy Analysis

The scanning electron microscopy (SEM) images of Zn@NMC are shown in Figure 4. Consistent with the results reported in the literature, doping NMC with metals led to the formation of a predominantly homogeneous, rough, and porous morphology. At lower magnifications, larger particles with smooth surfaces were observed [61].

**FIGURE 4:**
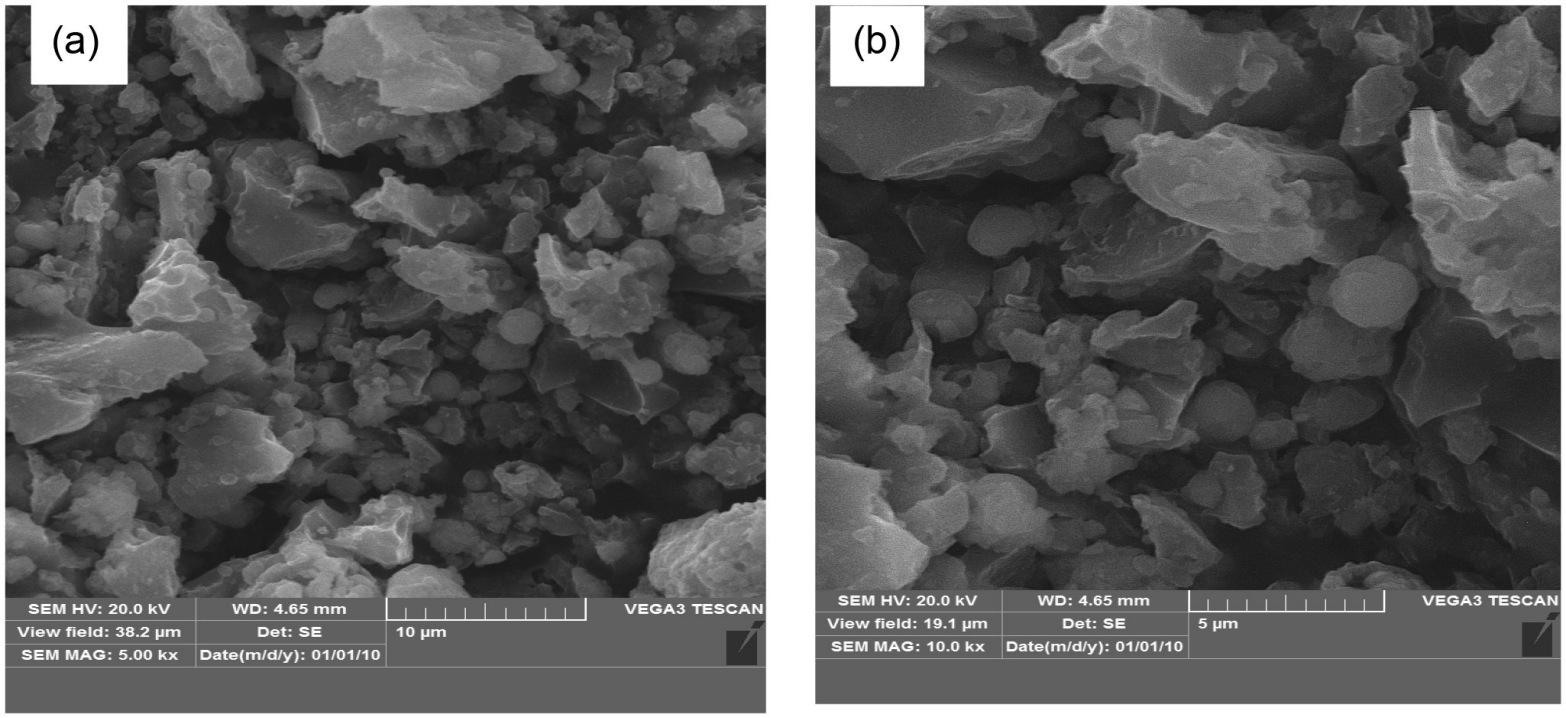
SEM images of Zn@NMC.

#### 3.1.5 Brunauer–Emmett–Teller Analysis

Figure 5a shows the N_2_ adsorption–desorption isotherm for the Zn@NMC composite. As per the classification set by the International Union of Pure and Applied Chemistry, this isotherm exhibits a type‐IV shape, indicative of mesoporous materials with pore sizes between 2 and 50 nm. The hysteresis loop reveals the existence of mesopores and the occurrence of capillary condensation within these pores. The steady rise in adsorbed volume up to a relative pressure of approximately 0.8, with a pronounced increase close to *P*/*P*_0_ = 1.0, verifies the presence of a mesoporous structure characterized by a high specific surface area, reaching a maximum adsorbed volume of approximately 18 cm^3^/g. The presence of mesopores and the process of capillary condensation within them are indicated by the hysteresis loop [62, 63, 64]. The plot of the pore size distribution (Figure 5b) reveals that the majority of pores are within the mesoporous range, predominantly featuring a primary peak at sizes under 10 nm. This suggests that the material primarily possesses a mesoporous structure, with a minor presence of micropores, resulting in a higher specific surface area and enhanced adsorption capabilities [65].

**FIGURE 5:**
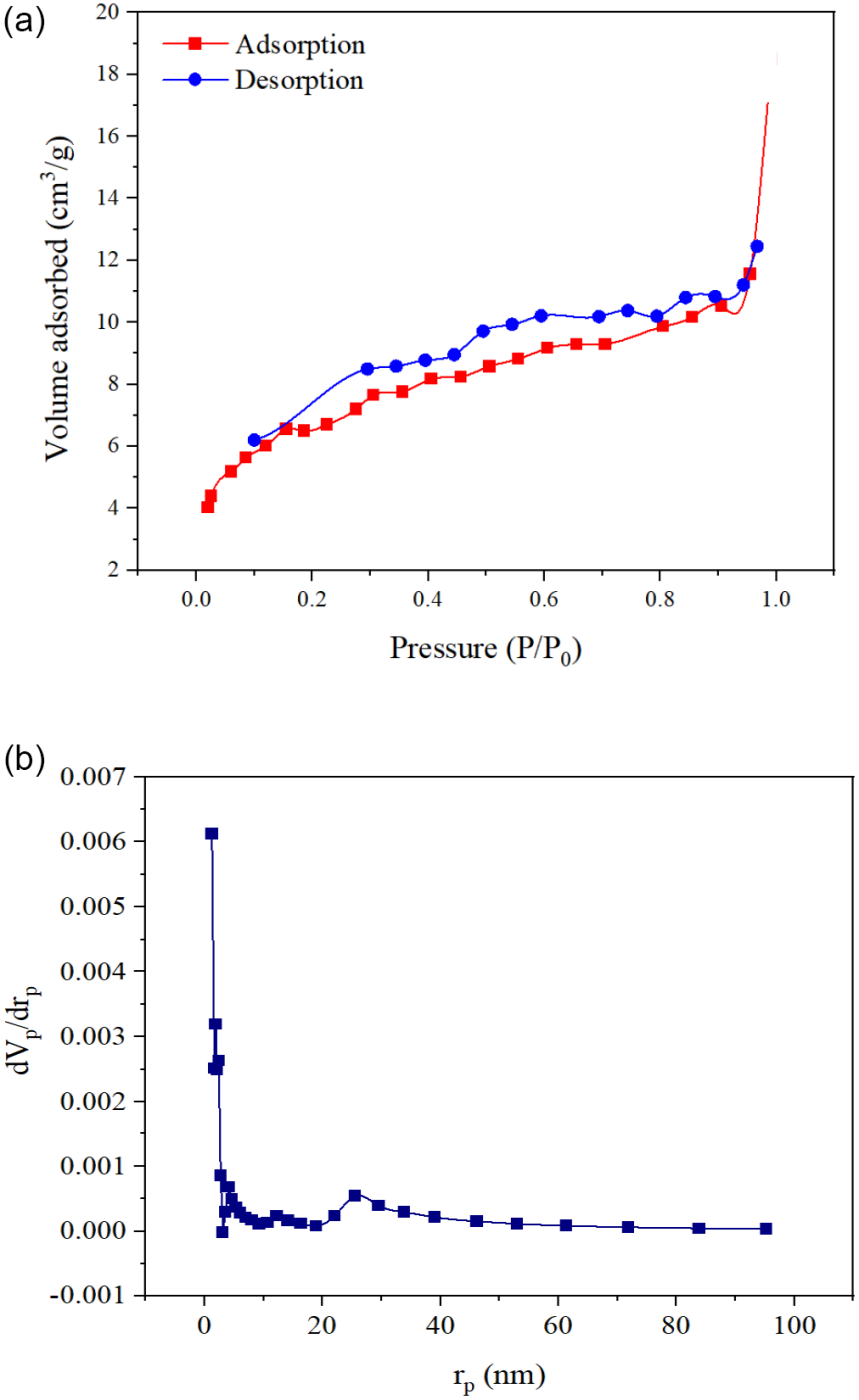
BET analysis of the nitrogen adsorption–desorption isotherm (a) and pore‐size distribution (b) of Zn@NMC.

Brunauer–Emmett–Teller (BET) measurements revealed that Zn@NMC possessed mesopores with a surface area of 300 m^2^/g and a total pore volume of 0.49 cm^3^/g, which is somewhat close to the surface area reported for NMC containing cerium and iron (357.6 m^2^/g) [66]. This relatively large specific surface area, combined with the availability of Zn‐ and nitrogen‐active sites, can significantly enhance the reaction rates and facilitate the reaction. The overall pore volume was determined to be 0.49 cm^3 ^g^−1^, which is lower than those documented for NMC in the existing scholarly literature. This suggests that the pores are partially filled with zinc metal, confirming the successful attachment of zinc to the carbon substrate [67]. The pores offer suitable surfaces for the entry of reactants, thus enabling efficient activation at the catalyst’s active sites.

#### 3.1.6 Thermogravimetric Analysis Analysis

The thermal behavior of Zn@NMC was investigated using thermogravimetric analysis (TGA) and differential thermal analysis (DTA) curves (Figure 6) in the temperature range from 32 to 692 °C. Upon heating to 692 °C, a steady weight reduction from 100% to approximately 77% was observed, suggesting the sequential breakdown of organic substances and the development of a carbon framework. The DTA curve reveals three notable endothermic peaks: The initial peak at 76.2 °C is linked to the evaporation of moisture and surface‐adsorbed solvents without significant chemical alterations; the second peak at 294.16 °C signifies the breakdown of organic materials and Zn–N complexes, leading to the emission of volatile organics and a steady decrease in weight; the third peak at 566.16 °C pertains to the carbonization and graphitization phase, during which mesoporous carbon is developed and most non‐carbon substances are eliminated [68]. Taken together, these findings suggest that the material maintains stability up to approximately 550 °C, thereby offering a pyrolysis route to achieve N‐doped mesoporous carbon with atomically distributed Zn‐active sites, which are suitable for electrocatalytic applications.

**FIGURE 6:**
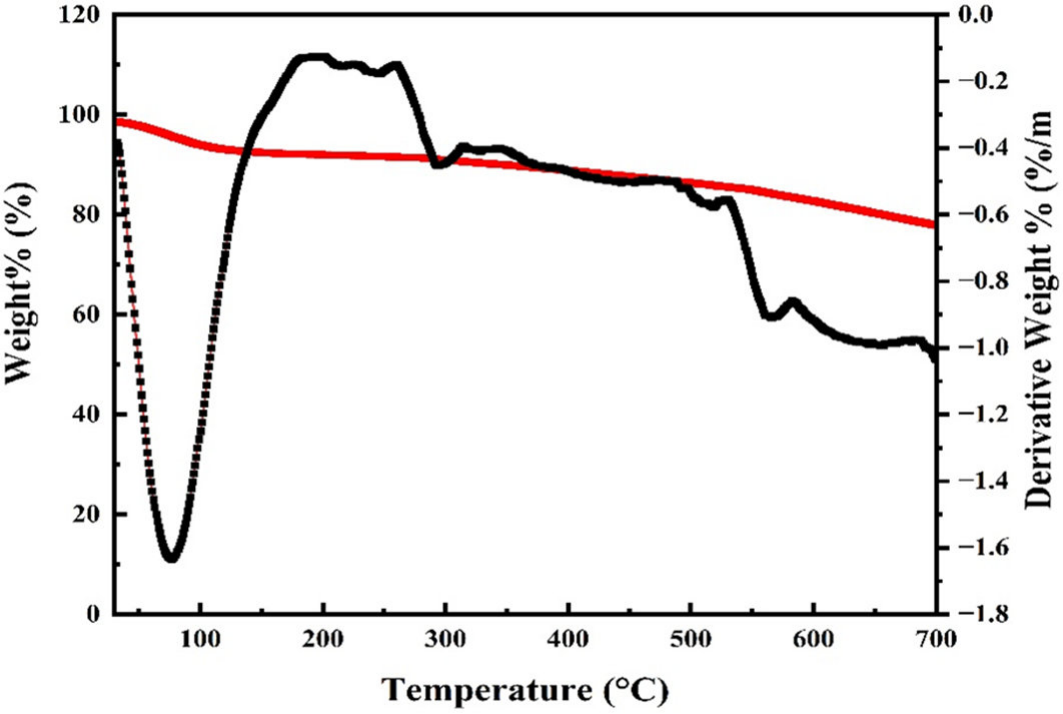
TGA/DTA analyses of Zn@NMC.

### 3.2 Model Reaction Optimization

The characteristics of an ideal synthesis include simplicity in the synthesis and purification steps while utilizing commercially available raw materials. After evaluating several synthetic strategies, the improved Radziszewski method was selected for the synthesis. In this method, the synthesis of 2,4‐diaryl‐*1H‐*imidazoles (Table 1) was achieved using ammonium acetate as the ammonia source, in combination with phenylglyoxal and benzaldehyde.

**TABLE 1:** Model reaction optimization.

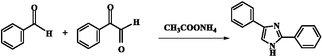					
Entry	Solvent	Catalyst/amount, mg	Time, min	Temp, °C	Yield, %
1	Methanol	—	12	25	N.R.
2	Methanol	ZnO NPs/50	30	40	21
3	Methanol	ZnCl_2_/50	30	40	15
4	Methanol	Zn@NMC/3	12	25	65
5	Methanol	Zn@NMC/5	12	25	78
6	Methanol	Zn@NMC/7	10	25	85
7	Methanol	Zn@NMC/7	12	25	97
8	Methanol	Zn@NMC/7	15	25	97
9	Methanol	Zn@NMC/7	18	25	95
10	Methanol	Zn@NMC/10	12	25	85
11	Methanol	Zn@NMC/15	12	25	81
12	Acetic acid	Zn@NMC/7	12	25	77
13	Acetic acid	Zn@NMC/7	12	Reflux	N.R.
14	Dichloromethane	Zn@NMC/7	12	25	45
15	*N*,*N*‐dimethylformamide	Zn@NMC/7	12	25	55
16	Ethanol	Zn@NMC/7	12	25	83
17	Methanol	Zn@NMC/7	12	30	85
18	Methanol	Zn@NMC/7	12	40	79
19	Methanol	Zn@NMC/7	12	60	74
20	Methanol	Zn@NMC/7	12	70	68
21	Methanol	Zn@NMC/7	12	80	63

Various parameters, including temperature, solvent type, reaction duration, and catalyst quantity, were evaluated to establish optimal conditions for the model reaction. The model reaction for the synthesis of phenylglyoxal (1) using benzaldehyde (2) as the substrate was studied in different solvents, including methanol, acetic acid, dichloromethane, and *N*,*N*‐dimethyl formamide, at various temperatures (25, 30, 40, 60, 70, 80 °C, and reflux conditions) in the presence of different amounts (3, 5, 7, 10, and 15 mg) of Zn@NMC as the nanocatalyst. The target product was not synthesized in the absence of the nanocatalyst (Table 1, entry 1). Therefore, the use of the Zn@NMC nanocatalyst is essential for this conversion. Accordingly, experiments were conducted with varying catalyst quantities to ascertain the optimal catalyst ratio (Table 1, entries 2–19). Optimal results were achieved using 7 mg of the Zn@NMC catalyst (Table 1, entry 2). Increasing the catalyst loading beyond 7 mg did not enhance the reaction efficiency (Table 1, entry 8).

The Zn‐N/OMC catalyst plays an important role in redox processes [69]. With an increase in the dose to 7 mg, all active sites became accessible to the reactants (phenylglyoxal and benzaldehyde), and further increases may lead to pore blockage or nanoparticle aggregation [70]. This result was also observed for similar catalysts, in which exceeding the optimal dose did not improve the yield of the reaction. For example, in the selective oxidation of benzyl alcohol to benzaldehyde using an NMC catalyst loaded with palladium (Pd/NOMC), increasing the dose from 10 to 20 mg reduced the conversion efficiency owing to catalyst aggregation [65].

Furthermore, we examined various solvents for synthesizing 2,4‐diaryl‐*1H*‐imidazoles (Table 1, entries 9–14). Three solvents were meticulously chosen for the synthesis process: dichloromethane (an aprotic nonpolar solvent), *N*,*N*‐dimethylformamide (an aprotic polar solvent), and methanol (a protic polar solvent); the corresponding reaction yields for each solvent are shown in Table 1. Methanol yielded the most substantial return (83%) in the synthesis of imidazole derivatives. This phenomenon can be ascribed to its exceptional efficacy resulting from its polar characteristics, which enhance imidazole formation. The protic nature of methanol enables the stabilization of ionic intermediates and transition states through hydrogen‐bonding interactions, which are critical during condensation and cyclization steps [71]. In contrast, dichloromethane and *N*,*N*‐dimethylformamide yielded the lowest yields.

Temperature evaluation revealed that 25 °C (ambient temperature) provided the highest efficiency (Table 1, entries 1–10 and 12–14). This can be attributed to the structural and chemical stability of the catalyst, as well as its activity at lower temperatures, where the catalytically active sites remain stable and accessible. The nitrogen sites in the catalyst effectively adsorbed the reactants at lower temperatures. In higher temperatures, the likelihood of harmful side reactions that negatively impact the overall yield of the main reaction increases significantly. Conversely, the occurrence of these side reactions was substantially reduced at 25 °C. Research has shown that metal catalysts fixed on carbon bases can facilitate processes, enabling multicomponent reactions to take place at room temperature [72, 73].

Additionally, as shown in this table, the reaction forming 2,4‐diaryl‐*1H*‐imidazoles was completed in 12 h with an excellent yield (96%). In fact, it can be said that at room temperature, the penetration of reactants into mesoporous cavities is somewhat time‐consuming. Still, the strong Zn/N interaction with the reactants provides sufficient residence time for the catalytic reaction to occur. Phenylglyoxal typically oscillates between hydrated, hemiacetal, and aldehyde tautomeric forms; optimal conditions are achievable when it exists in an optimal ratio of these three forms (Scheme 3).

**SCHEME 3:**
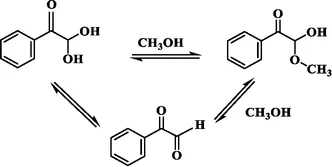
Likely equilibrium present among the tautomers of phenylglyoxal.

Under optimized conditions, 2,4‐diaryl‐*1H‐*imidazoles were synthesized with various substituents, incorporating both electron‐withdrawing and electron‐donating groups such as bromine(─Br), chlorine(─Cl), and methyl(─CH_3_), at *the ortho*, *meta*, and *para* positions (Table 2). The goal of this study was to investigate starting materials with different electronic properties in the central aromatic core. All compounds were obtained in high yields (76%–90%) without chromatographic purification. Notably, chlorinated or brominated substrates produced higher yields of the desired product in shorter reaction times when chlorine or bromine was in the *para* position (88% and 90%, respectively, for **3g**, **3h,** and **3i**). Chlorine or bromine substituents at the *meta* or *ortho* positions resulted in lower yields (75% and 79% for entries **3b**, **3f,** and **3l**), demonstrating an even more substantial negative effect on the product yield compared to the electron‐donating methyl group (87% and 80% yields for **3d** and **3h**).

**TABLE 2:** Synthesis of some imidazole derivatives using a catalyst.

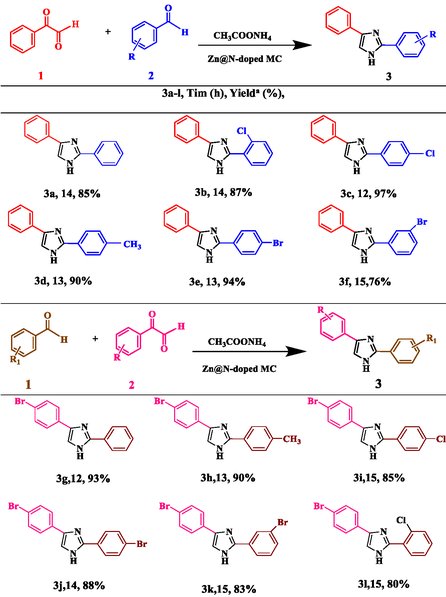

a Isolated yields.

An NMC nanocomposite catalyst containing zinc (Zn@NMC), which provides atomic‐level active zinc sites on hierarchically porous carbon structures, enabled the synthesis of imidazole derivatives from dicarbonyl compounds and aldehydes. The zinc atoms stabilized in the NMC matrix act as Lewis acid centers that coordinate the carbonyl groups of both α‐dicarbonyl and aldehyde reactants, activating the carbonyl groups for nucleophilic attack [40, 68]. The mesopores maximize the intraparticle diffusion rates of the reactants and enhance access to the nitrogen‐ and zinc‐active sites. Nitrogen doping creates electron‐rich environments and stabilizes the zinc atoms [40, 74]. Additionally, the nitrogen‐doped carbon surface activates ammonium acetate by providing basic sites through deprotonation, generating ammonia in situ.

#### 3.2.1 Proposed Reaction Mechanism

Based on the literature, the reaction likely proceeds via a multicomponent condensation pathway involving phenylglyoxal, an aldehyde, and ammonium acetate as the nitrogen source. The proposed reaction steps are as follows (Scheme 4):

**SCHEME 4:**
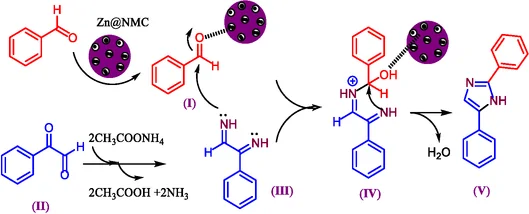
General synthesis mechanism of 2,4(5)‐diarylimidazoles.

Step 1: Activation of the Aldehyde: In this step, the carbonyl group of the aldehyde (I) coordinates with zinc, which acts as a Lewis acid center in the Zn@NMC catalyst. This coordination enhances the electrophilic character of the carbonyl carbon, making it more susceptible to nucleophilic attack.
Step 2: Nucleophilic attack of ammonia/ammonium acetate: Ammonia from ammonium acetate attacks the carbonyl carbons of phenylglyoxal (**II**), forming a carbinolamine intermediate (**IV**).
Step 3: Dehydration to form an imine: Carbinolamine undergoes dehydration to yield an imine. The mesoporous catalyst structure aids this reaction by enhancing mass transfer and eliminating water as a by‐product. Importantly, when ammonia is added to a carbonyl carbon, it releases one water molecule from the carbinolamine intermediate, leading to the formation of compound **IV**. The carbonyl carbons of phenylglyoxal (**II**) form a carbinolamine intermediate (**IV**).
Step 4: Nucleophilic attack and cyclization: The zinc catalyst simultaneously coordinates to one of the phenylglyoxal carbonyl carbon groups, whereas the imine acts as a nucleophile. The nitrogen atom attacks the remaining phenylglyoxal carbonyl carbon, triggering intramolecular cyclization. This forms a five‐membered aromatic imidazole ring (**V**) and releases another water molecule.

### 3.3 Comparison of the Catalyst Performance With the Literature

In this section, we compare the Zn@NMC nanocatalyst with other reported catalysts used for the synthesis of imidazole derivatives to evaluate their advantages (Table 3). The results clearly demonstrate that the Zn@NMC nanocomposite (7 mg) outperformed the other nanocomposites in terms of reaction time and temperature. The use of water at ambient temperature for the reaction, along with a small amount of nanocatalyst, is an eco‐friendly strategy for synthesizing amides. Studies have shown that nitrogen‐rich materials (NMCs), which are mainly composed of carbon and nitrogen, are nontoxic and pose no harm to the environment [57]. Moreover, considering the minimal zinc content in the NMCs, it can be inferred that Zn@NMC demonstrates strong environmental compatibility.

**TABLE 3:** Comparison of the present study with previous studies.

Compound	Catalyst	Amount of catalyst	Optimal condition	Efficiency	References
Diarylmethylamines	1,1′‐bi‐2‐naphthol	10 mol% 1 g	Toluene at −40 °C for 12 h	92%–97%	[75]
2,5‐Diarylimidazoles	Pd(OAc)2	5 mol%	Pd(OAc)_2_, xylene, 140 °C, 24 h	27%–71%	[26]
1,2,5‍‐‍Trisubstituted imidazoles	Benzoic acid	10 mol%	Toluene at 100 °C for 8 h	70%–85%	[76]
1‐Benzyl‐2,4‐diarylimidazole	Iodine/CuI	10 mol%	Toluene at 100 °C for 24 h	85%	[25]
Trisubstituted imidazole derivatives	NiCoFe_2_O_4_	0.004 g (4 mg) of catalyst.	Solvent free at 110 °C for 5 min	95%	[77]
1,4‐Diarylimidazoles	Ammonium acetate	30 mmol for 3 mmol	Acetic acid at 100 °C for 4–5 h	72%–92%	[78]
1‐Aryl‐4‐(5‐nitrofuran‐2‐il)imidazoles	Ammonium acetate	30 mmol for 3 mmol	Acetic acid at 100 °C for 4–5 h	72%–92%	[78]
2,4(5)‐Diarylimidazoles	Zn@NMC	7 mg	Methanol, 25 °C, for 12 h	75%–90%	Present work

## 4 Reusability of the Catalyst

Considering the importance of catalyst recovery and reuse, the performance of the catalyst after six cycles of reuse was investigated (see the results in Figure 7). After six recycling cycles, the yield showed only a slight decrease, and the catalyst performance remained excellent. For future reuse in reactions, the catalyst was extracted using an external magnet, thoroughly washed several times with ethanol and acetone, and dried at room temperature for 6 h. The effectiveness of the catalyst was attributed to its fast reaction time and high product yield. The formation of the products was confirmed by the physical and spectral data collected from these compounds. To examine the stability of the catalyst, an SEM image of the recovered catalyst was taken. The image in Figure 8a shows that the morphology of the catalyst remained unchanged after reuse, demonstrating its structural resistance. Furthermore, the XRD pattern analysis of the recovered catalyst, as shown in Figure 8b, revealed that the catalyst remained reusable even after multiple cycles of use. In addition, a hot filtration test was performed in the model reaction for the synthesis of 3a to investigate the possible leaching of the Zn@N‐doped mesoporous carbon (Zn@NMC) catalyst. After 3 minutes of reaction, the catalyst was removed from the reaction mixture using a strong external magnet. Thin‐layer chromatography was employed to monitor the progress of the reaction, which showed no further progress [79, 80, 81].

**FIGURE 7:**
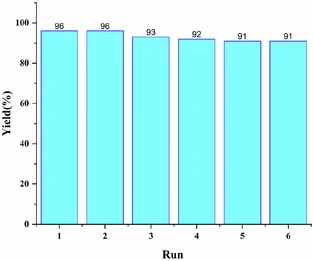
The reusability of Zn@N‐doped mesoporous carbon (Zn@NMC).

**FIGURE 8:**
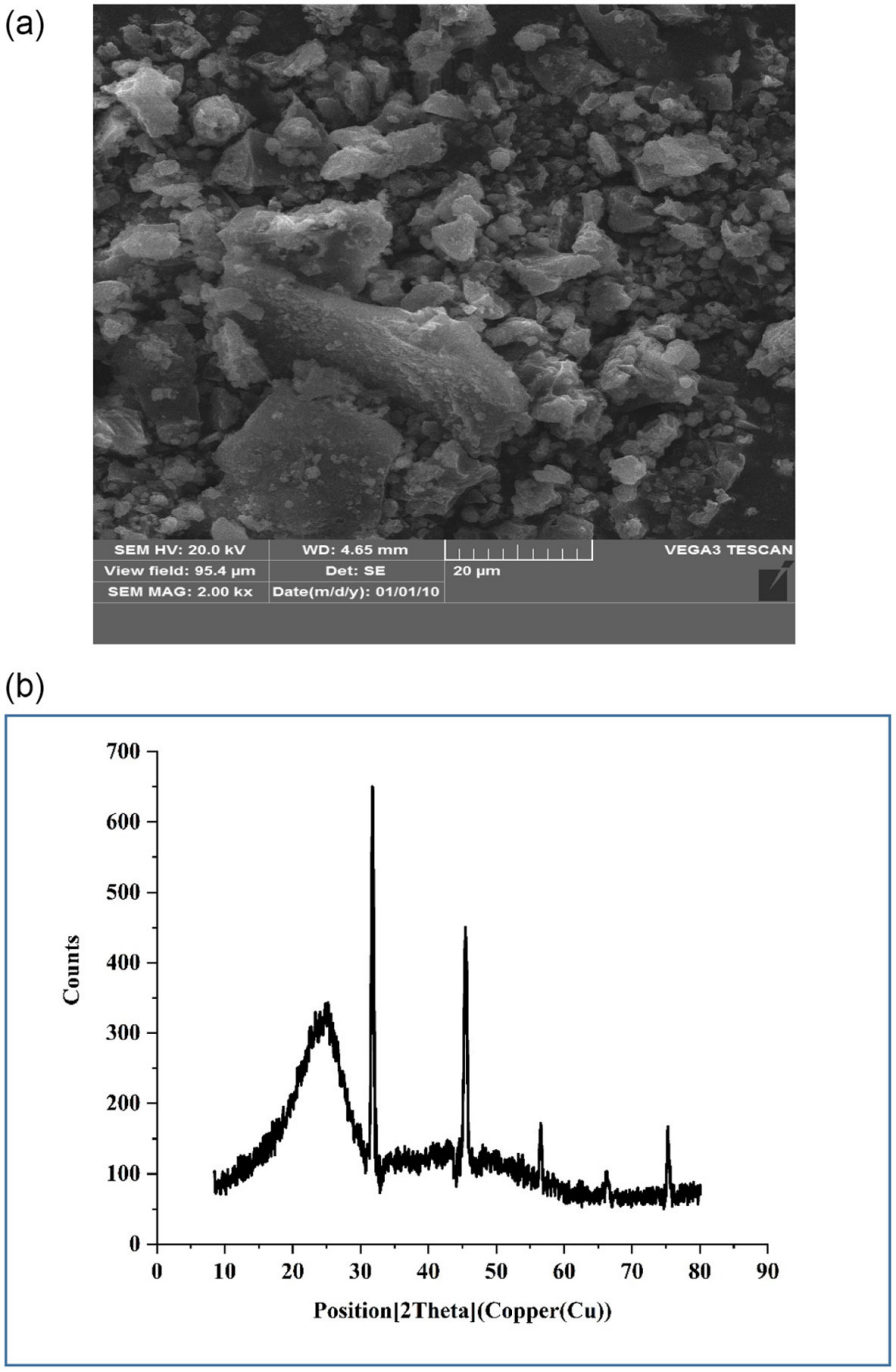
SEM (a) images of the Zn@N‐doped mesoporous carbon (Zn@NMC) after reuse. XRD (b) image after reuse of the Zn@N‐doped mesoporous carbon (Zn@NMC).

## 5 Conclusion

The primary objectives of this study were to develop Zn@N‐doped mesoporous carbon (Zn@NMC) via solvent EISA and to apply it in the synthesis of 2,4(5)‐diarylimidazoles. Following a detailed analysis of the catalyst through techniques such as FT‐IR, XRD, EDX, SEM, and BET, the optimal conditions for synthesizing 2,4(5)‐diarylimidazoles were accurately established. The results showed that using 7 mg of the catalyst and employing a methanol solvent at ambient temperature (25 °C) for 12 h achieved maximum efficiency. By utilizing the synergistic effects of single‐atom zinc sites and nitrogen‐rich carbon matrices, high selectivity for 2,4(5)‐diarylimidazoles was accomplished, and common challenges, such as side reactions and catalyst deactivation, were resolved. The use of a low catalyst loading and ambient temperature synthesis aligns with green chemistry principles and reduces energy consumption, demonstrating its great potential as a sustainable and efficient platform for the large‐scale synthesis of pharmaceutical scaffolds.

## Conflicts of Interest

The authors declare no conflicts of interest.

## Supporting information

Some characteristics of the catalyst and products are described in the Supporting Information available through the publisher’s site.

## Data Availability

The data that support the findings of this study are available in the Supporting Information of this article.
