# Supplementary material for: Nitrogen‐Doped Mesoporous Carbon Loaded With Zn Single Atoms as a Catalyst for the Synthesis of 2,4(5)‐Diarylimidazoles
Source: ChemistryOpen. 2026 Oct 13;15(11):e70318. doi: 10.1002/open.70318 (PMC13661297; doi:10.1002/open.70318)
Supplement: Supplementary file 1 [file OPEN-15-e70318-s001.pdf]

**SUPPORTING INFORMATION**

# Nitrogen-Doped Mesoporous Carbon Loaded with Zn Single Atoms as a Catalyst for the Synthesis of 2,4(5)-Diarylimidazoles

Maryam Shojaei-Asl and Mohsen Moradian*

Department of Organic Chemistry, Faculty of Chemistry, University of Kashan, Kashan, Iran.

Corresponding Author Email: m.moradian@kashanu.ac.ir

Colleague Email: shojmaryam68@gmail.com

**Table of Contents**

| | |
|---|---|
| General Experimental Method | S2 |
| Spectroscopic and analytical data for compounds 3a-3l and phenylglyoxal | S3-S22 |

## General Experimental Method

The $^{1}H$-NMR spectra were recorded on a 400 MHz instrument; Chemical shifts (δ scale) are expressed in parts per million (ppm) relative to the internal standard of the solvent's central resonance. $^{1}H$-NMR spectral data are presented by listing the multiplicity, the approximate J coupling constant in hertz (Hz), and the proton count. Peaks are designated as s for singlet, d for doublet, t for triplet, m for multiplet, or br s for broad singlet. The $^{13}C$-NMR spectra were obtained using a 100 MHz instrument, with chemical shifts (δ) expressed in parts per million (ppm) on the δ scale.

## General procedure for the synthesis of 2,4(5)-diarylimidazoles 3a-3l.

12 mL of dioxane was added to a round-bottomed flask. Subsequently, 10 mmol of selenium dioxide and 1 mL of distilled water were added to the flask. The temperature of the reaction mixture was slowly raised to 55 °C to improve the dissolution of selenium dioxide. Following this, 10 mmol of acetophenone was added to the reaction mixture, and the reaction was carried out under reflux at 100 °C for 7 hours. After the reaction was completed, the solution was carefully processed to separate the solid precipitate from the liquid component. Afterward, distilled water was added to the remaining solution, and the resulting mixture was heated to 100 °C with a mechanical stirrer for a specified period to achieve the desired concentration. To facilitate the crystallization process, the solution was stored in a refrigerated environment. Ultimately, the precipitate was collected through filtration and then rinsed with distilled water to ensure its purity.

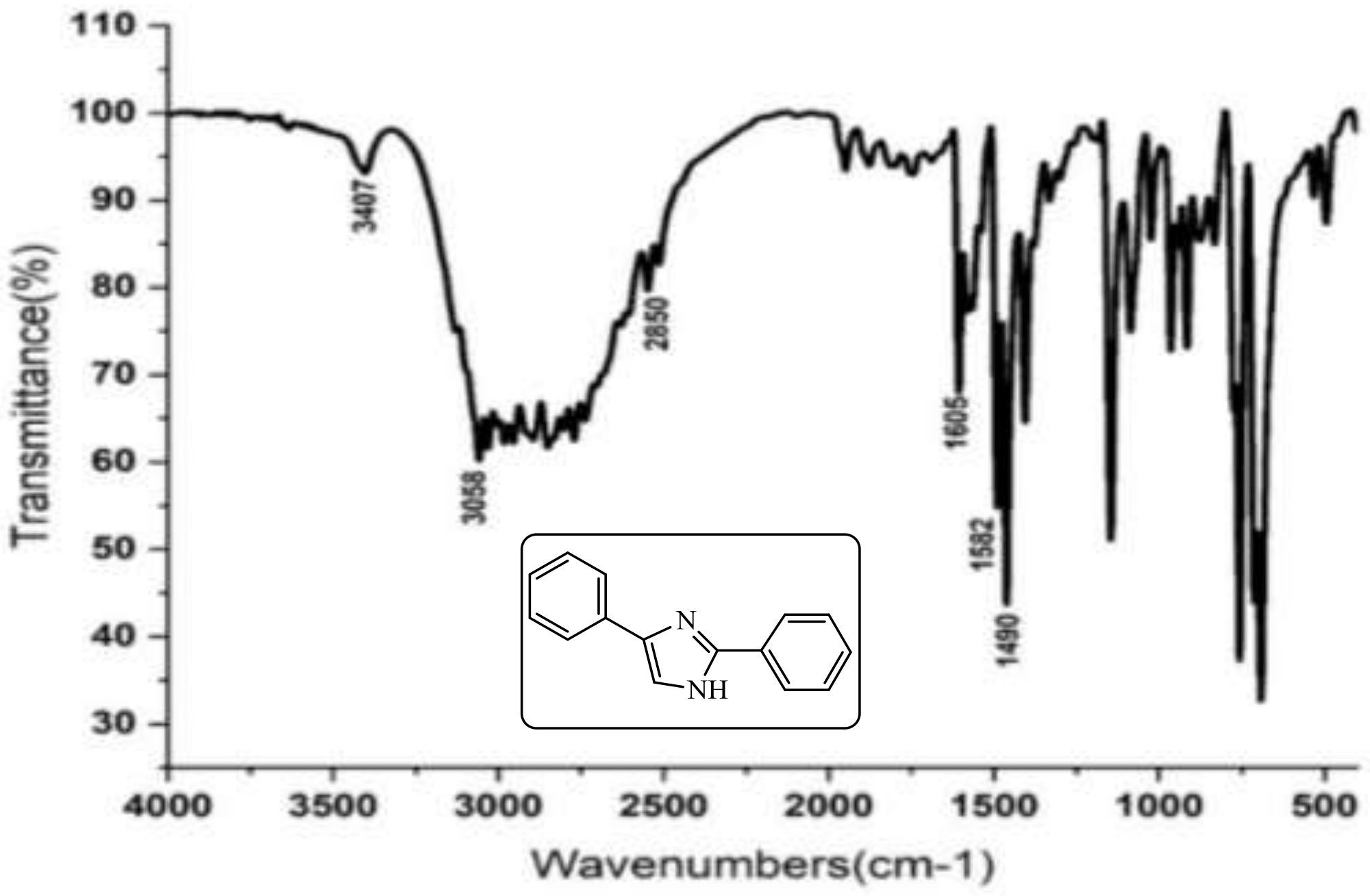


**Figure S1**: The FT-IR spectrum of compound 2,4(5)-diphenylimidazole (**3a**)

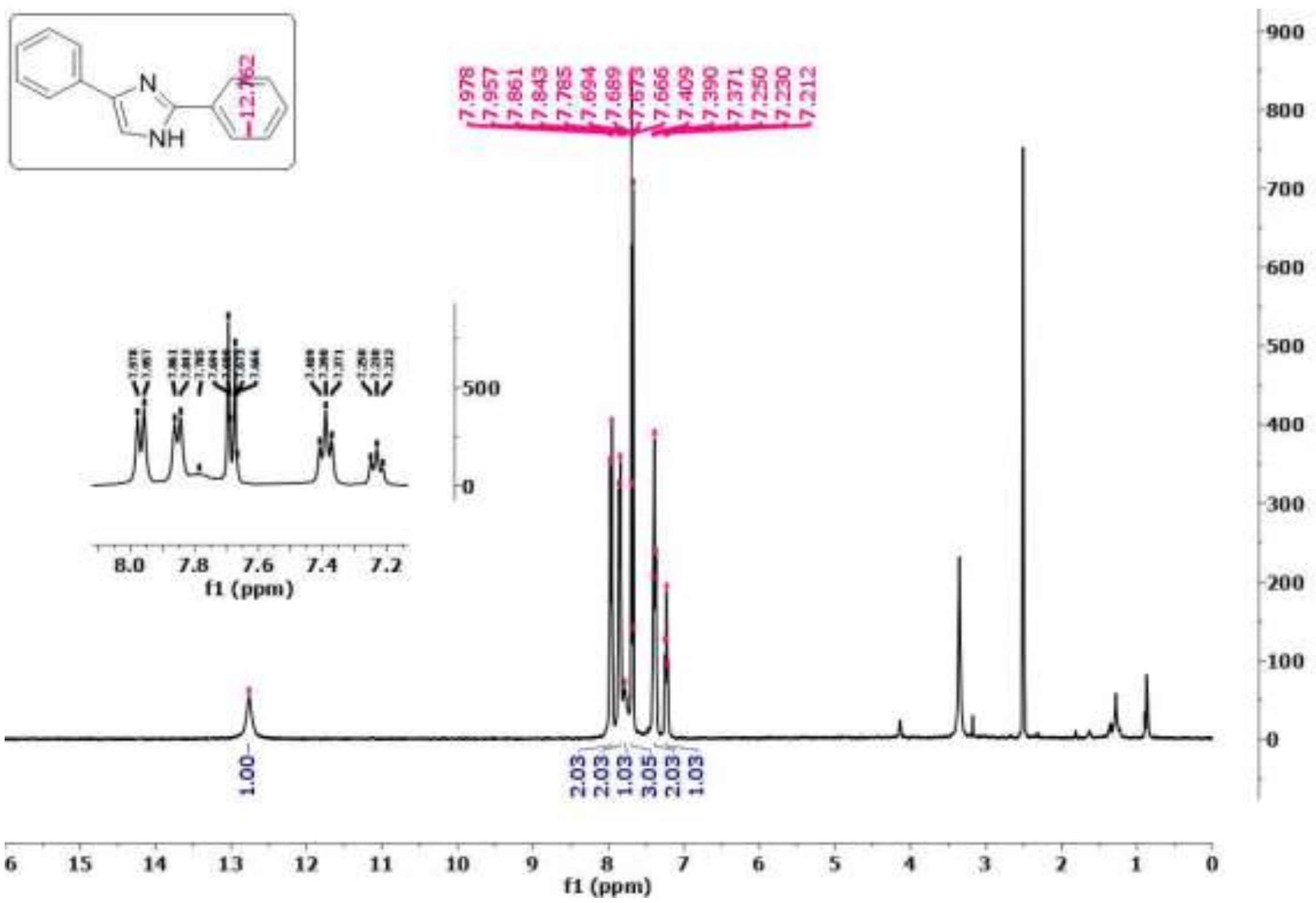


**Figure S2**: The $^{1}$H NMR spectrum of compound 2,4(5)-diphenylimidazole (**3a**)

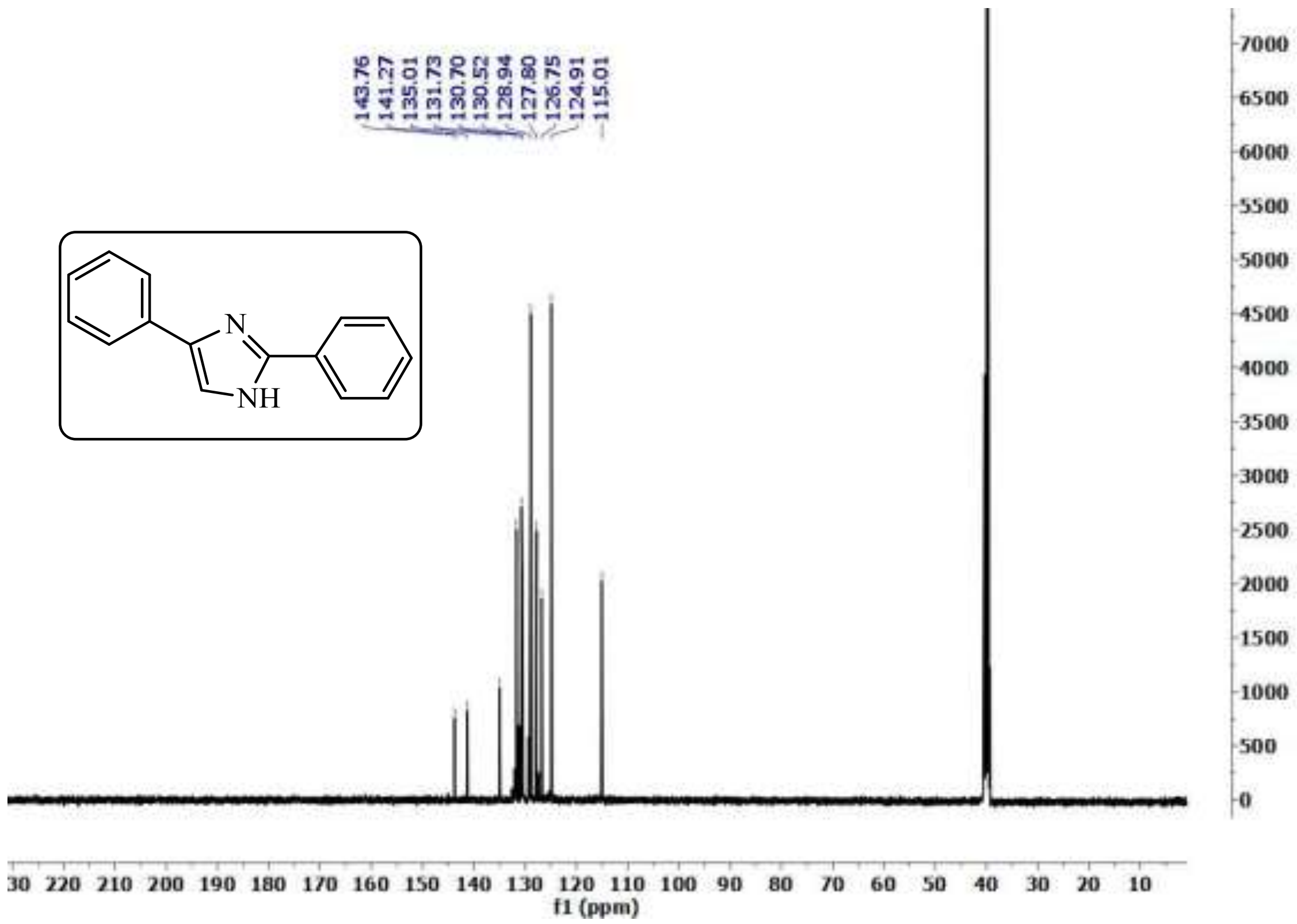


**Figure S3**: The $^{13}$C NMR spectrum of compound 2,4(5)-diphenylimidazole (**3a**)

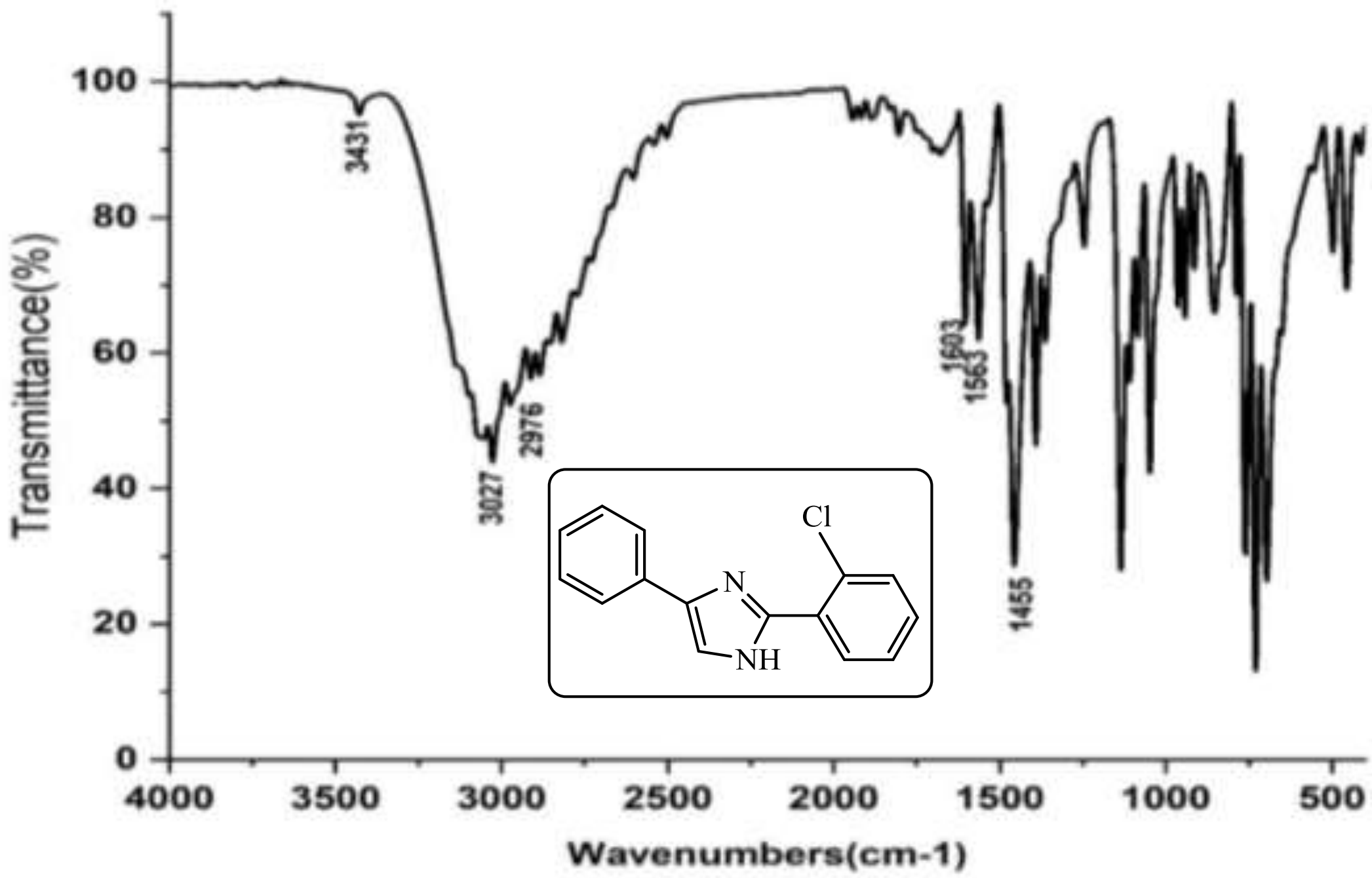


**Figure S4**: The FT-IR spectrum of compound 2-(2-Chlorophenyl)-4(5) –phenylimidazole (**3b**)

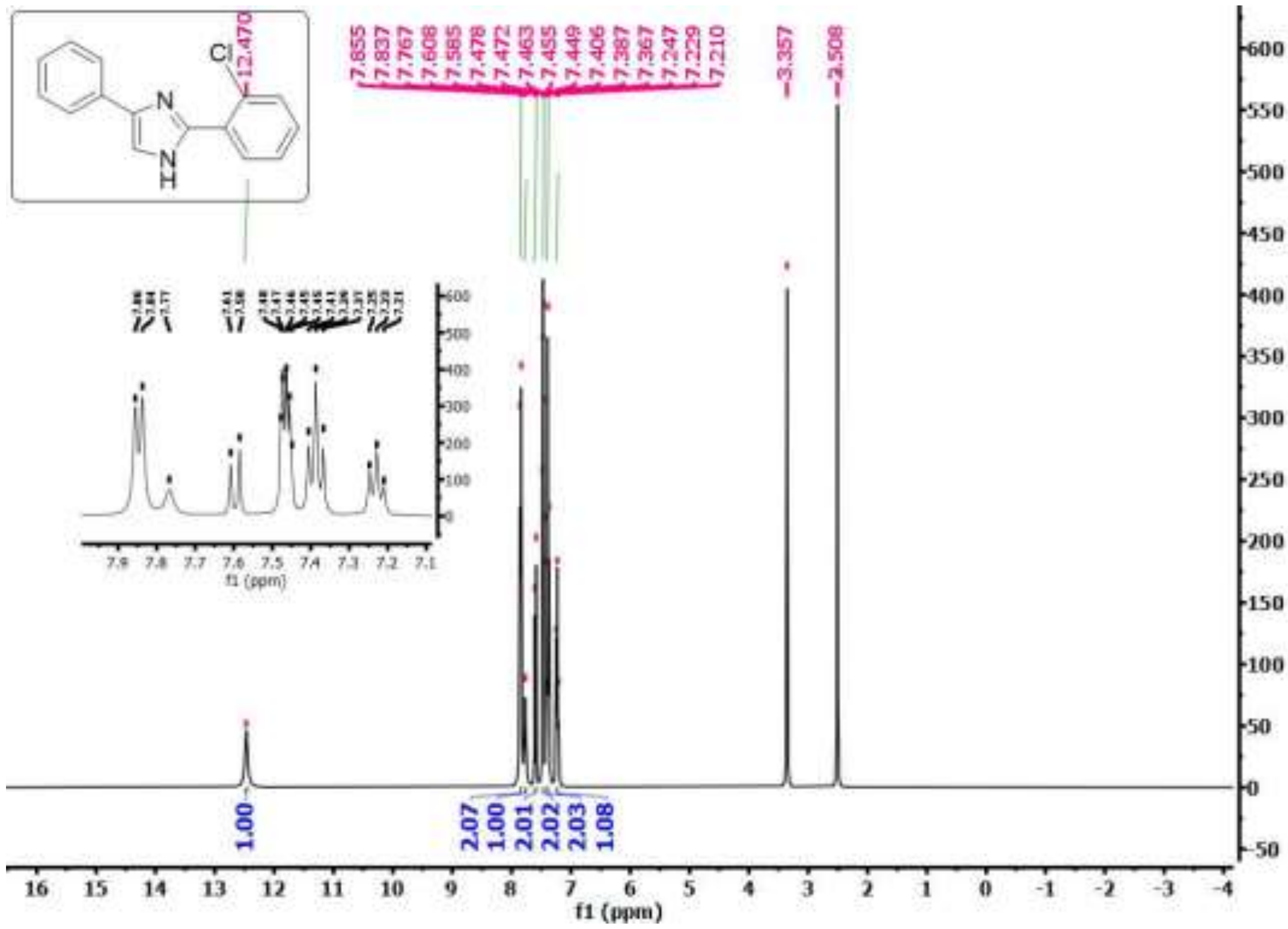


**Figure S5**: The $^{1}H$ NMR spectrum of compound 2-(2-Chlorophenyl)-4(5) –phenylimidazole (**3b**)

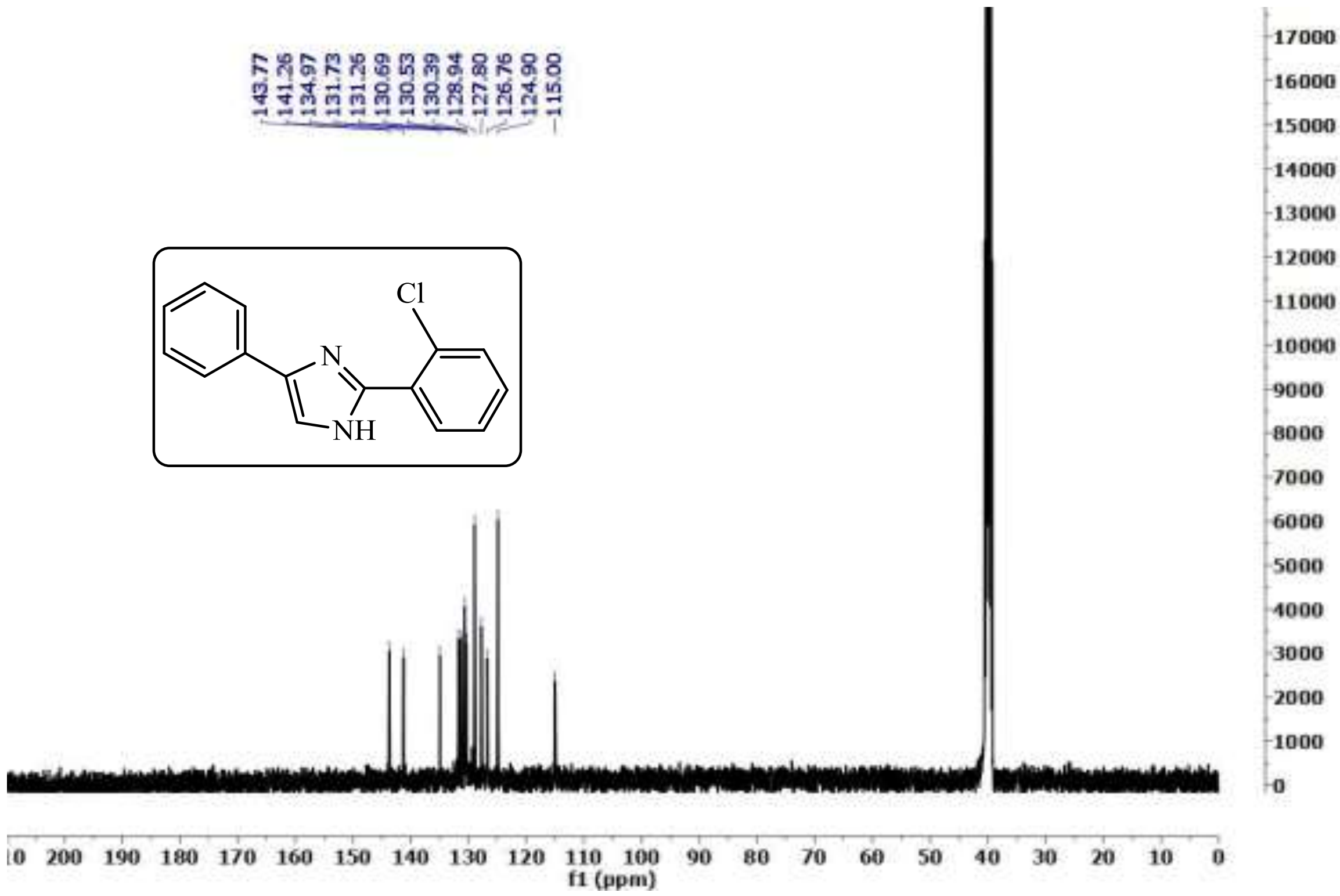


**Figure S6**: The $^{13}C$ NMR spectrum of compound 2-(2-Chlorophenyl)-4(5) –phenylimidazole (**3b**)

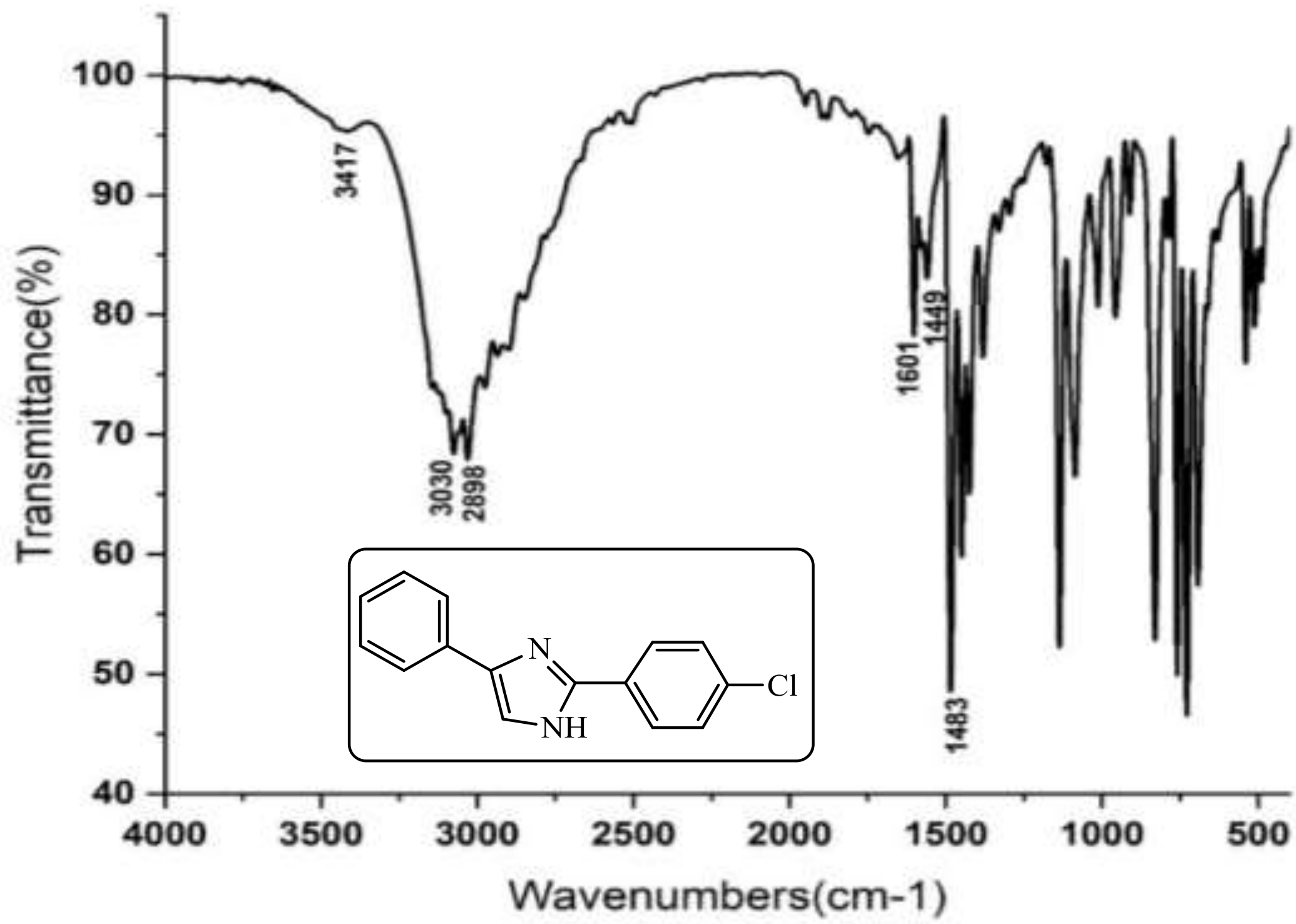


**Figure S7**: The FT-IR spectrum of compound 2-(4-Chlorophenyl)-4(5) – phenyl imidazole (**3c**)

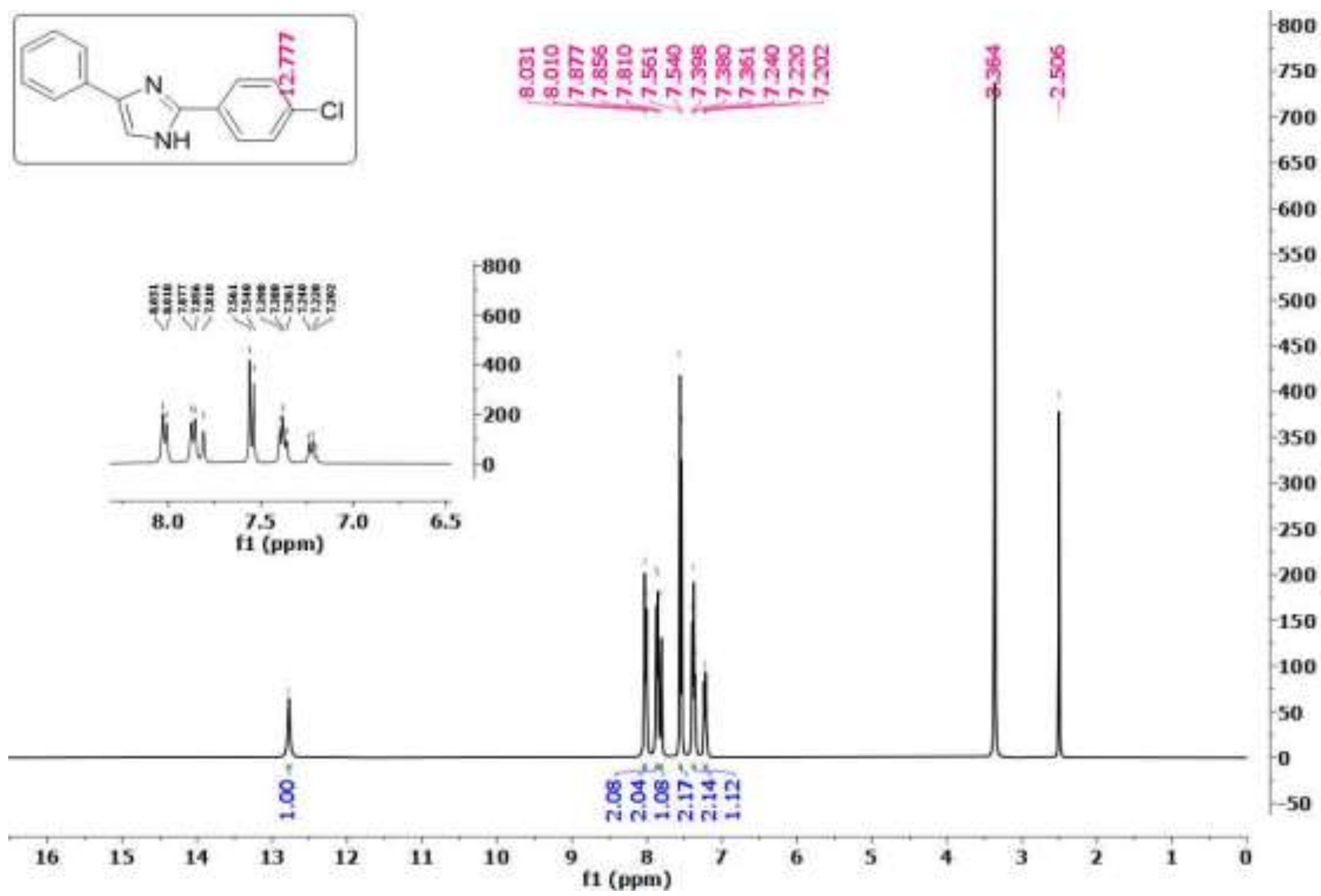


**Figure S8**: The $^{1}$H NMR spectrum of compound 2-(4-Chlorophenyl)-4(5) – phenyl imidazole (**3c**)

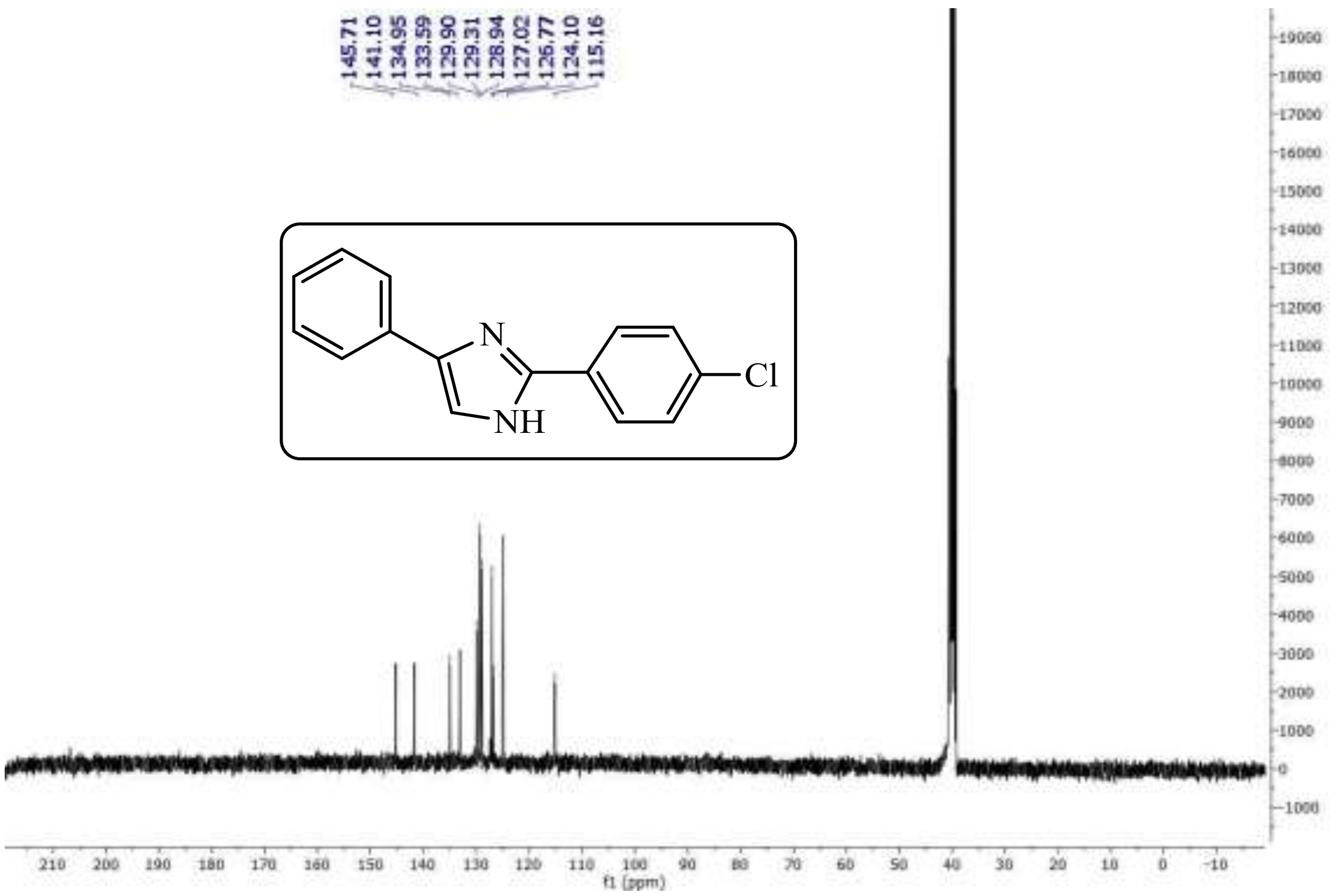


**Figure S9**: The $^{13}$C NMR spectrum of compound 2-(4-Chlorophenyl)-4(5) – phenyl imidazole (**3c**)

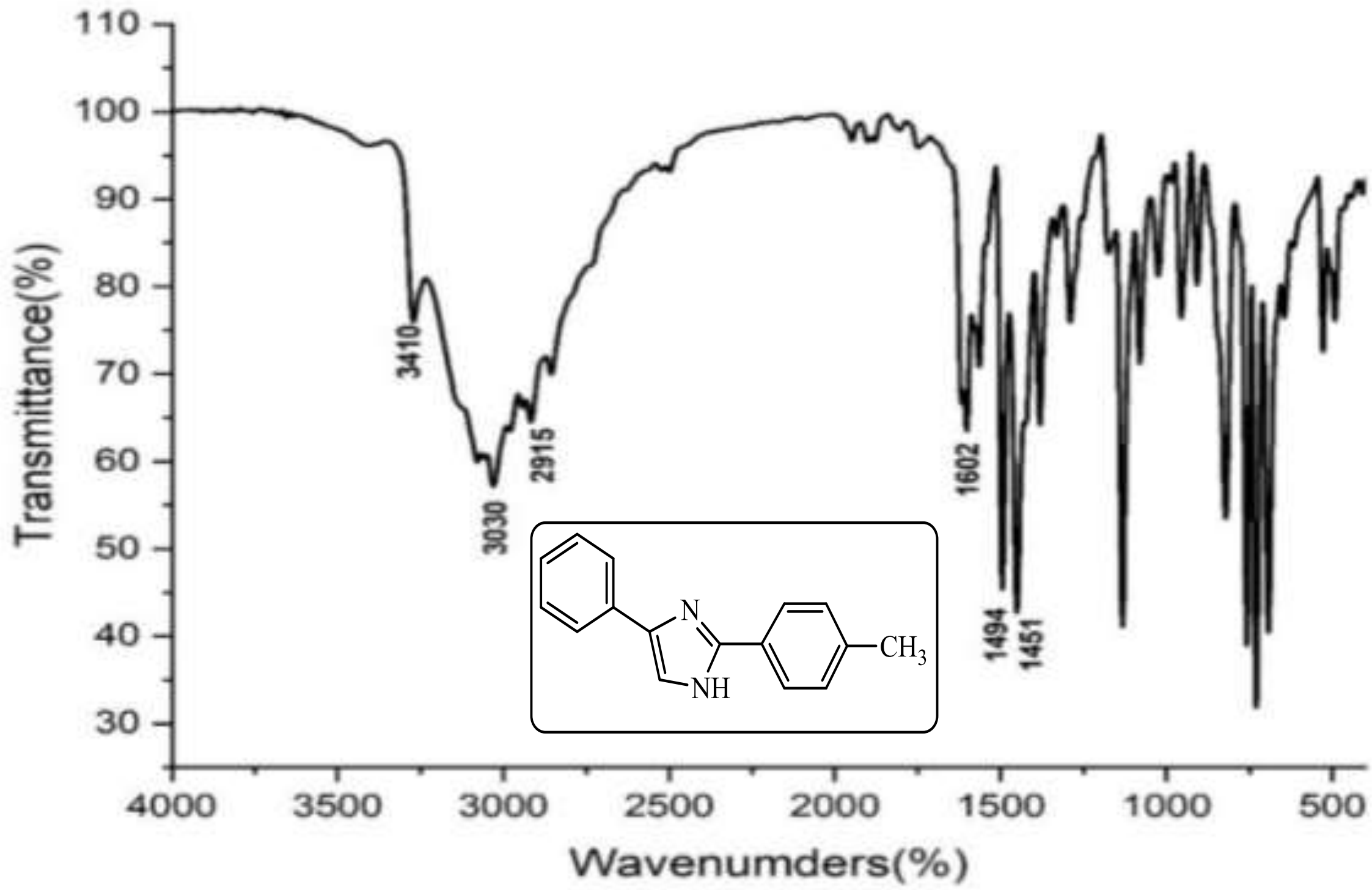


**Figure S10**: The FT-IR spectrum of compound 2-(4-methylphenyl)-4(5) – phenyl imidazole (**3d**)

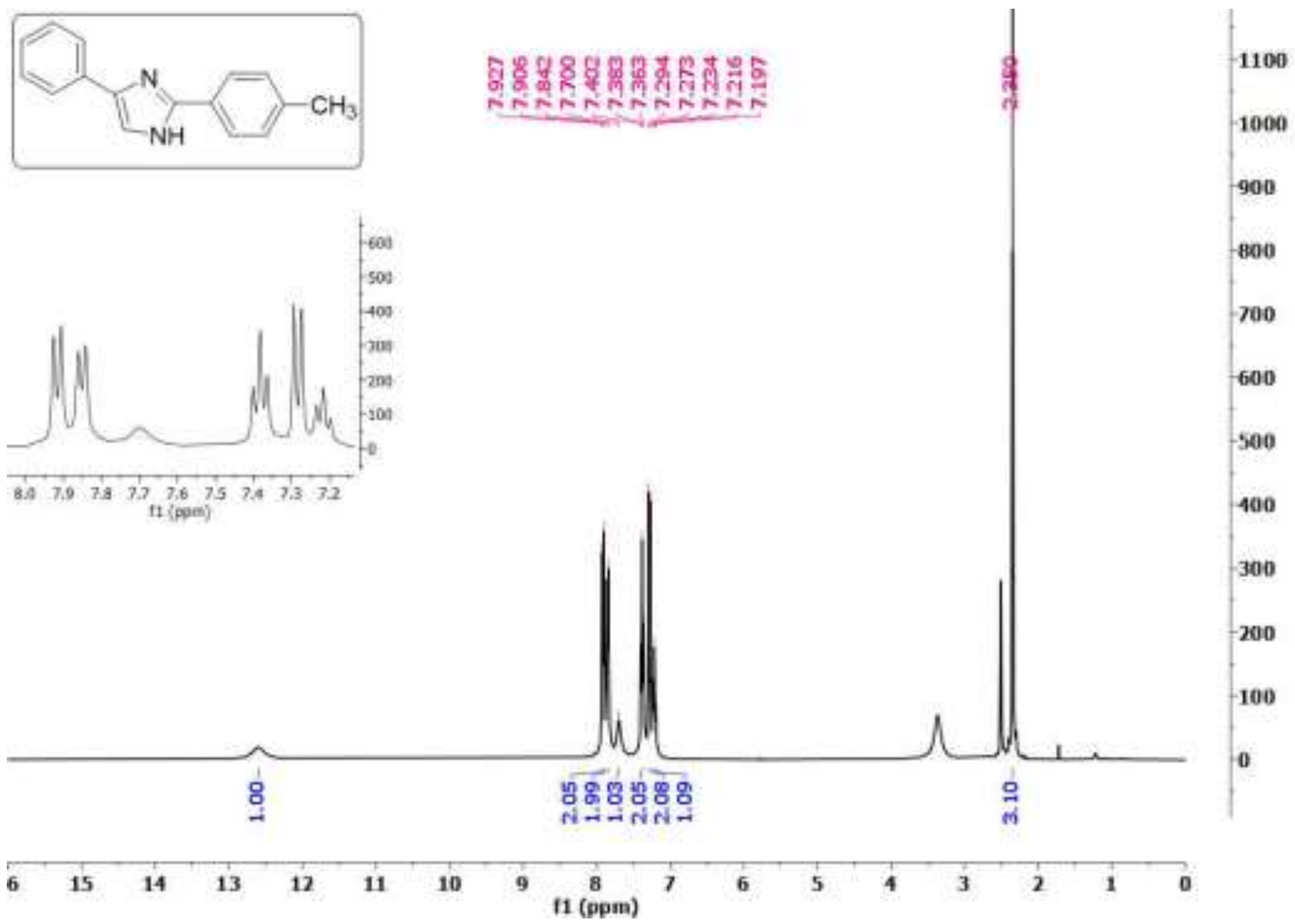


**Figure S11**: The $^{1}$H NMR spectrum of compound 2-(4-methylphenyl)-4(5) – phenyl imidazole (**3d**)

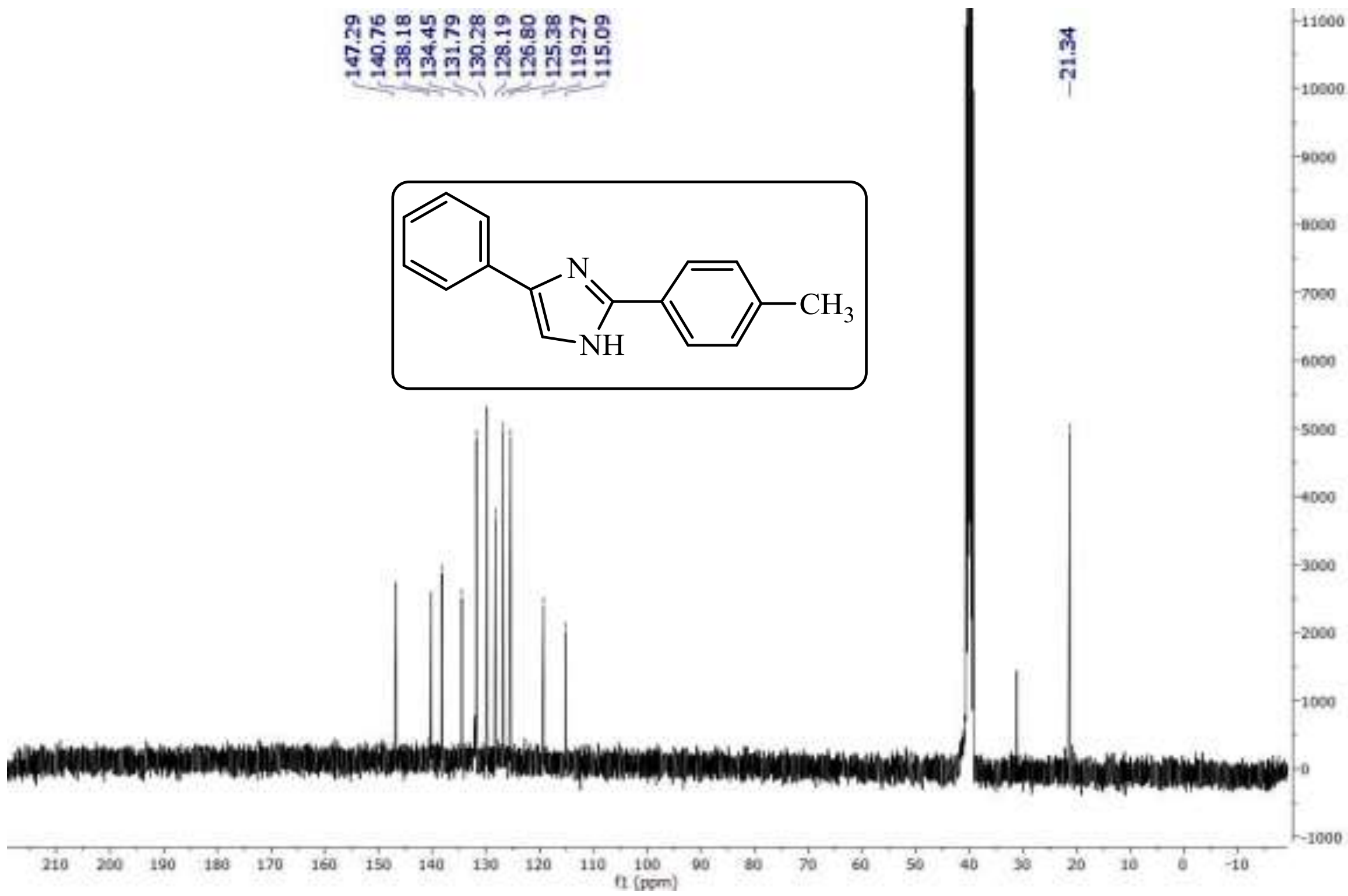


**Figure S12**: The $^{13}$C NMR spectrum of compound 2-(4-methylphenyl)-4(5) – phenyl imidazole (**3d**)

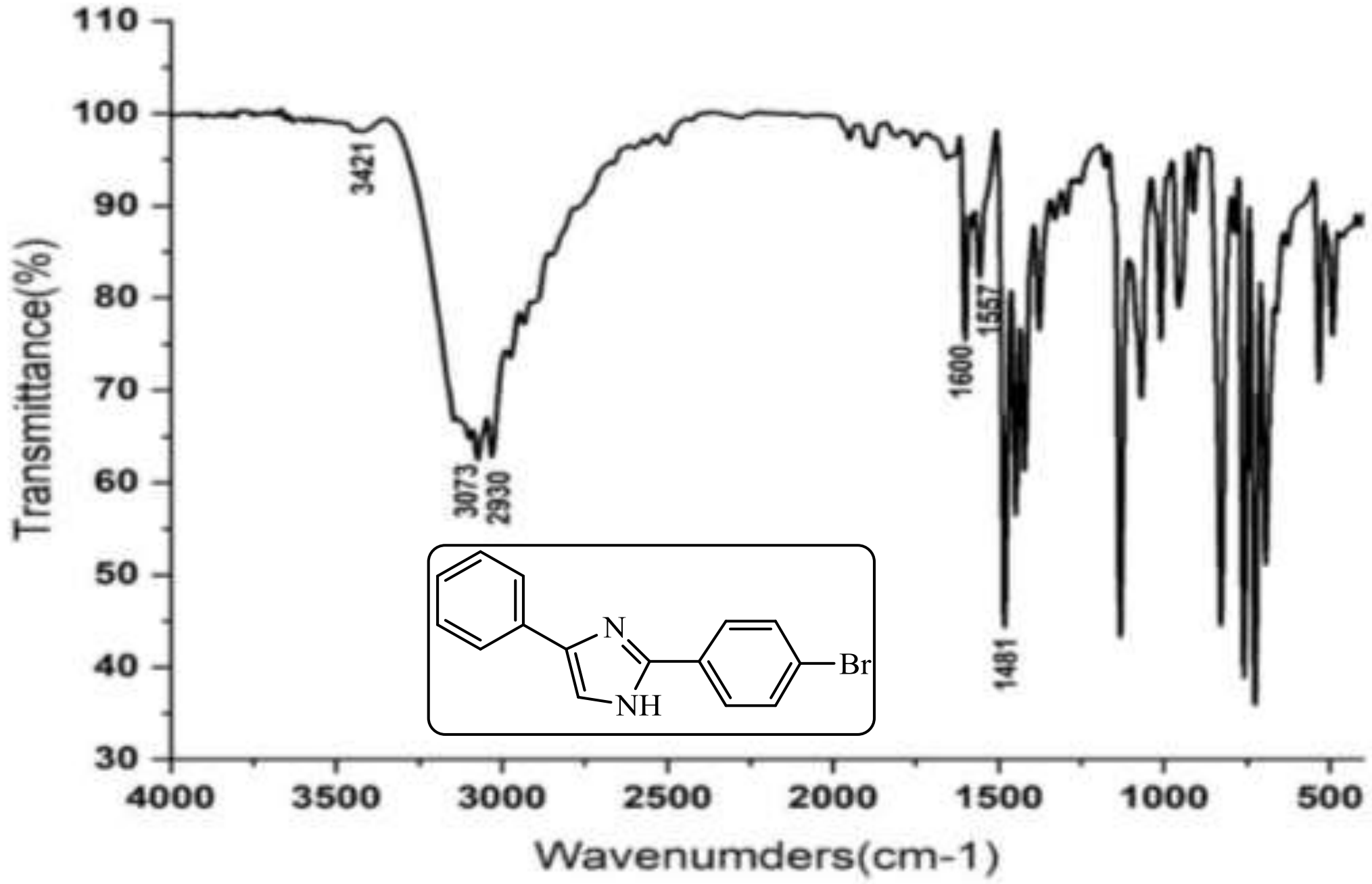


**Figure S13**: The FT-IR spectrum of compound **2-(4-Boromophenyl)-4(5) – phenyl imidazole (3e)**

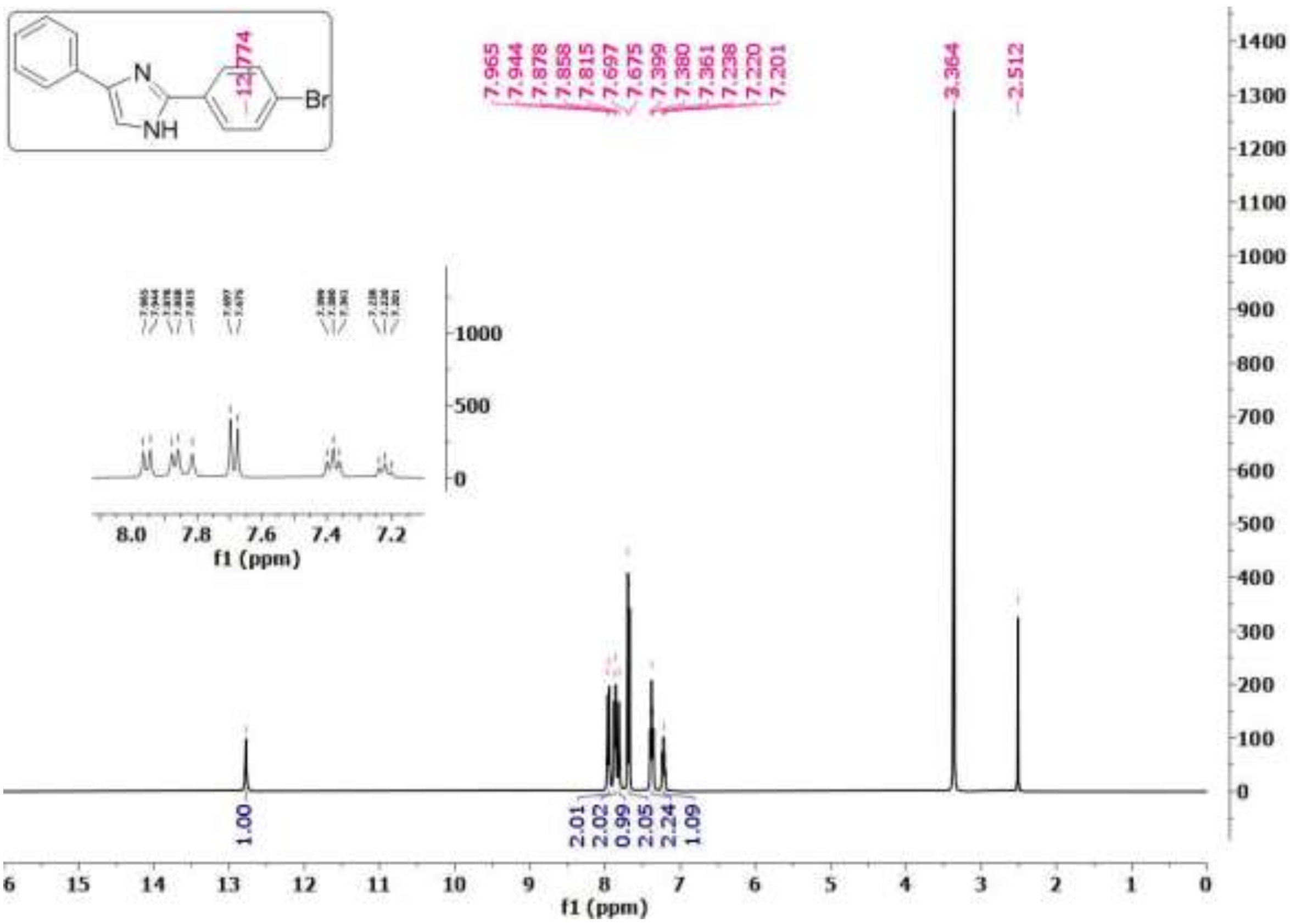


**Figure S14**: The ¹H NMR spectrum of compound **2-(4-Boromophenyl)-4(5) – phenyl imidazole (3e)**

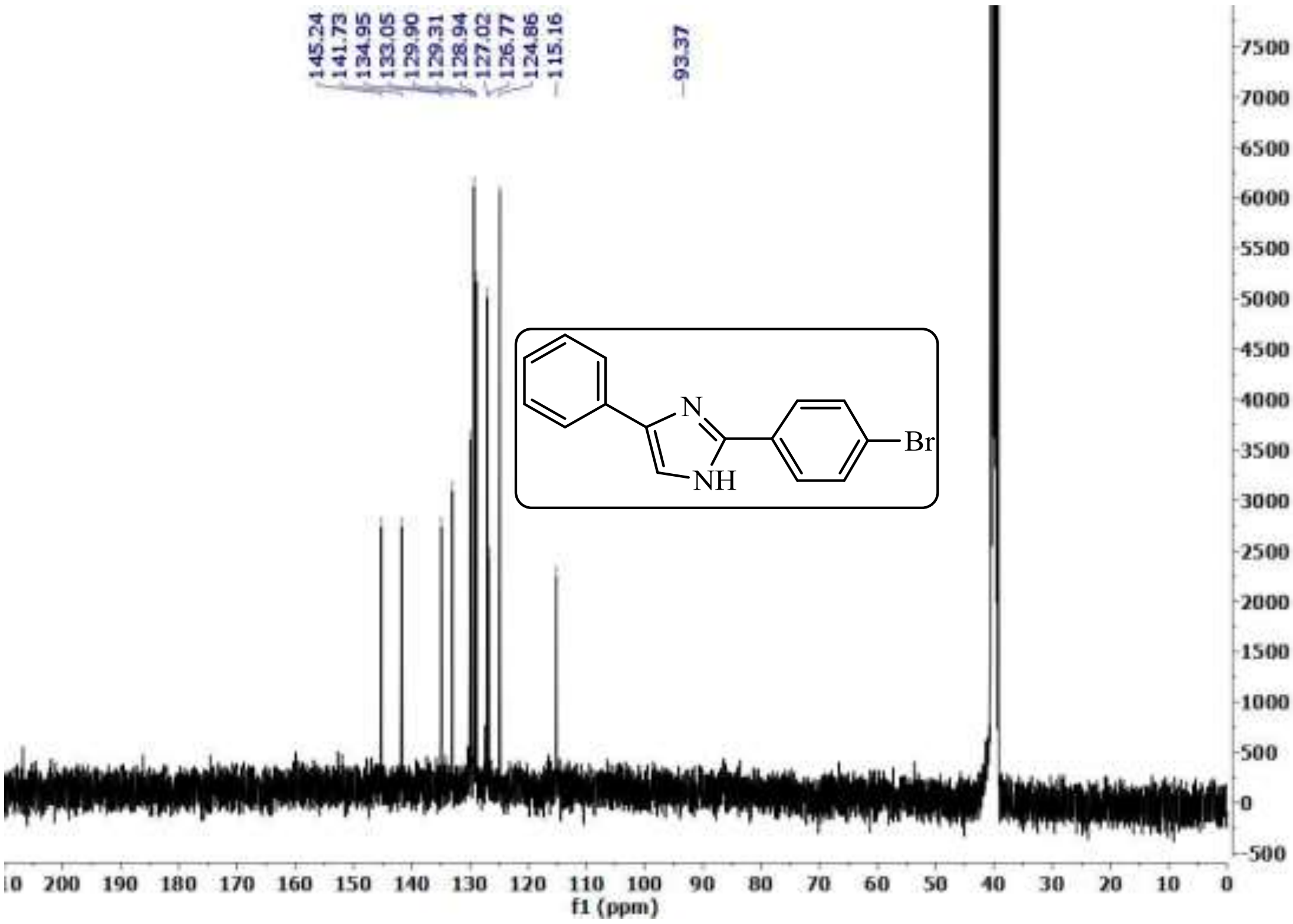


**Figure S15**: The $^{13}$C NMR spectrum of compound **2-(4-Boromophenyl)-4(5) – phenyl imidazole (3e)**

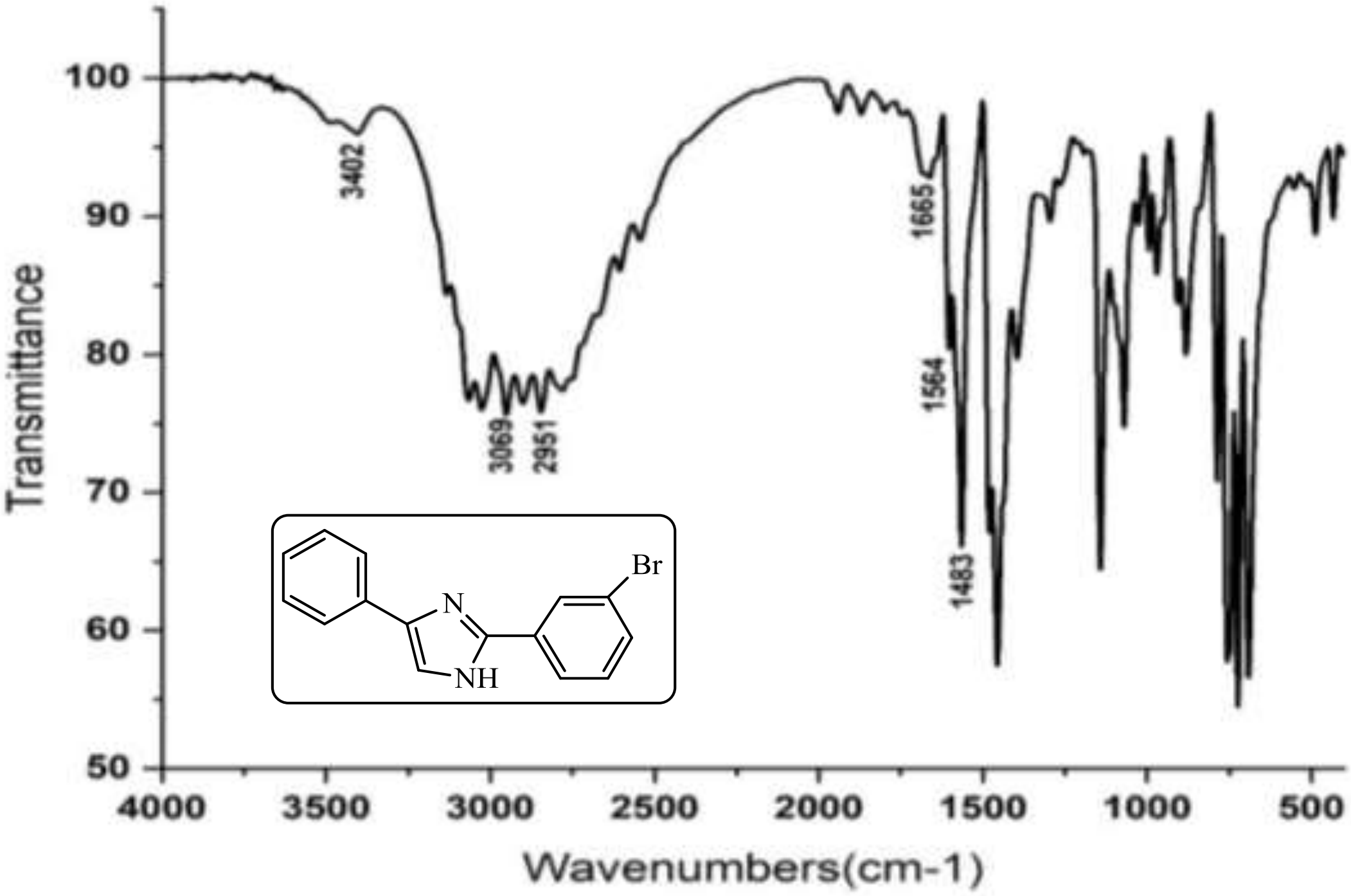


**Figure S16**: The FT-IR spectrum of compound **2-(3-Boromophenyl)-4(5) – phenyl imidazole (3f)**

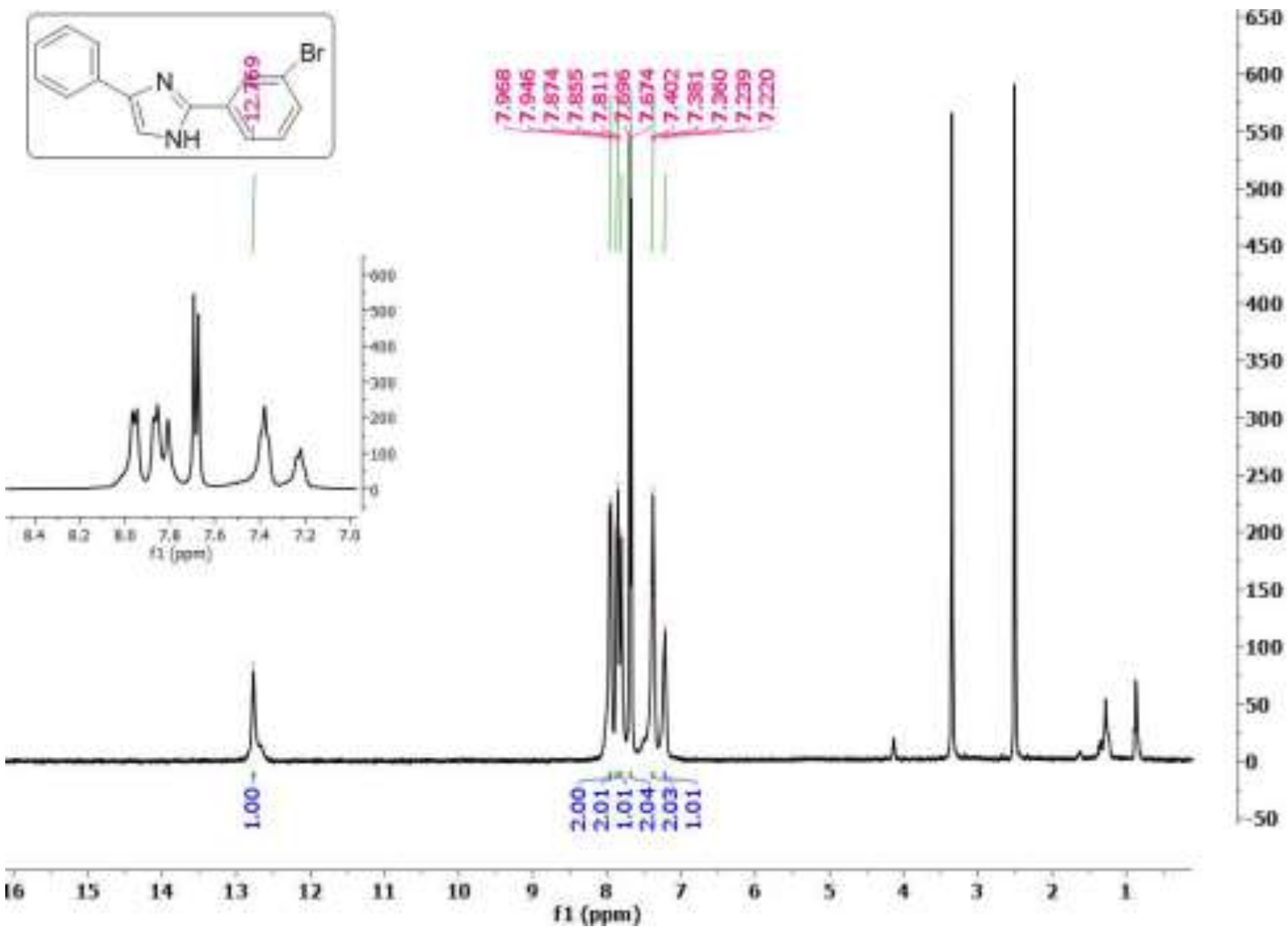


**Figure S17**: The $^{1}$H NMR spectrum of compound **2-(3-Boromophenyl)-4(5) – phenyl imidazole (3f)**

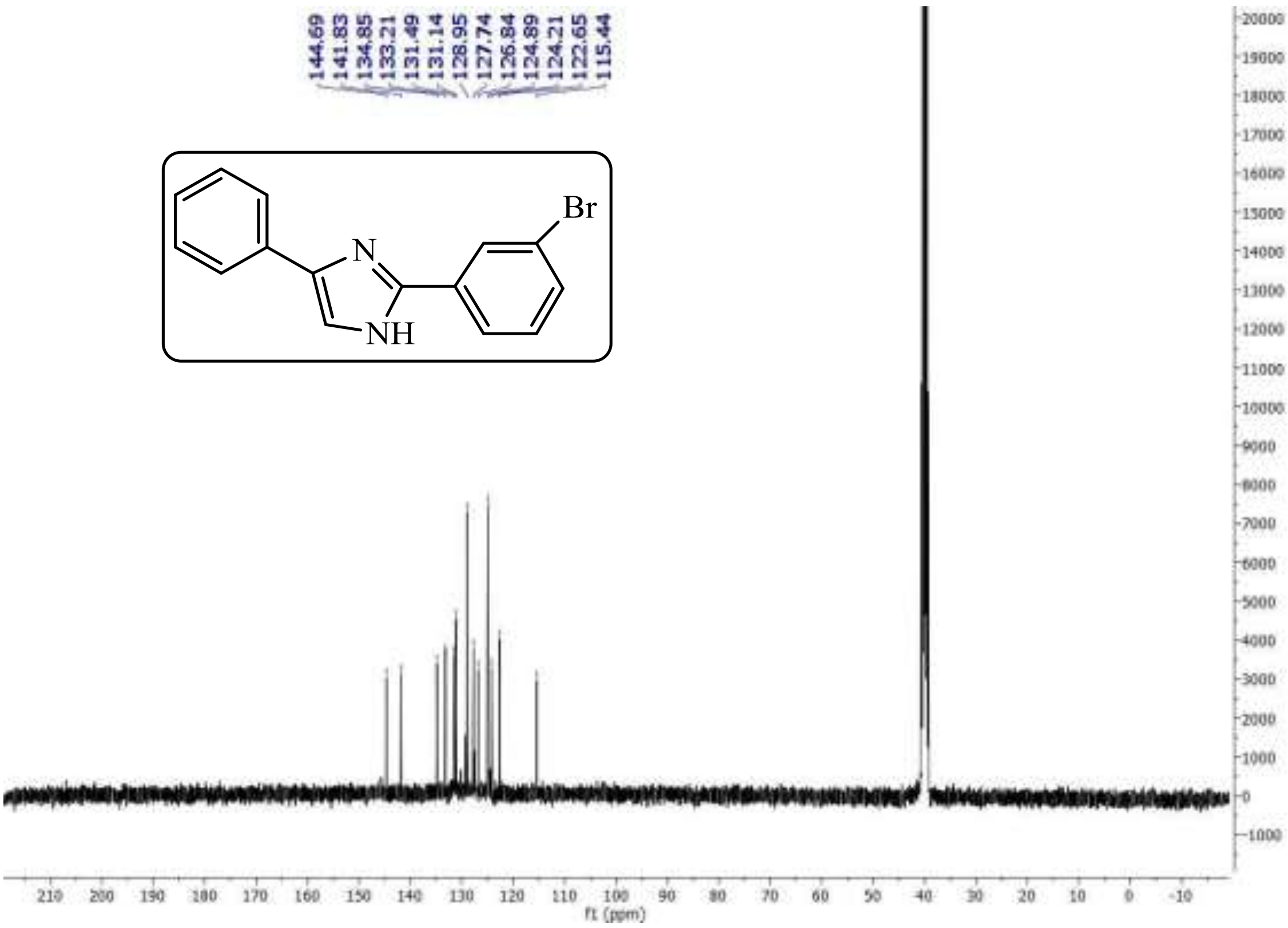


**Figure S18**: The $^{13}$C NMR spectrum of compound **2-(3-Boromophenyl)-4(5) – phenyl imidazole (3f)**

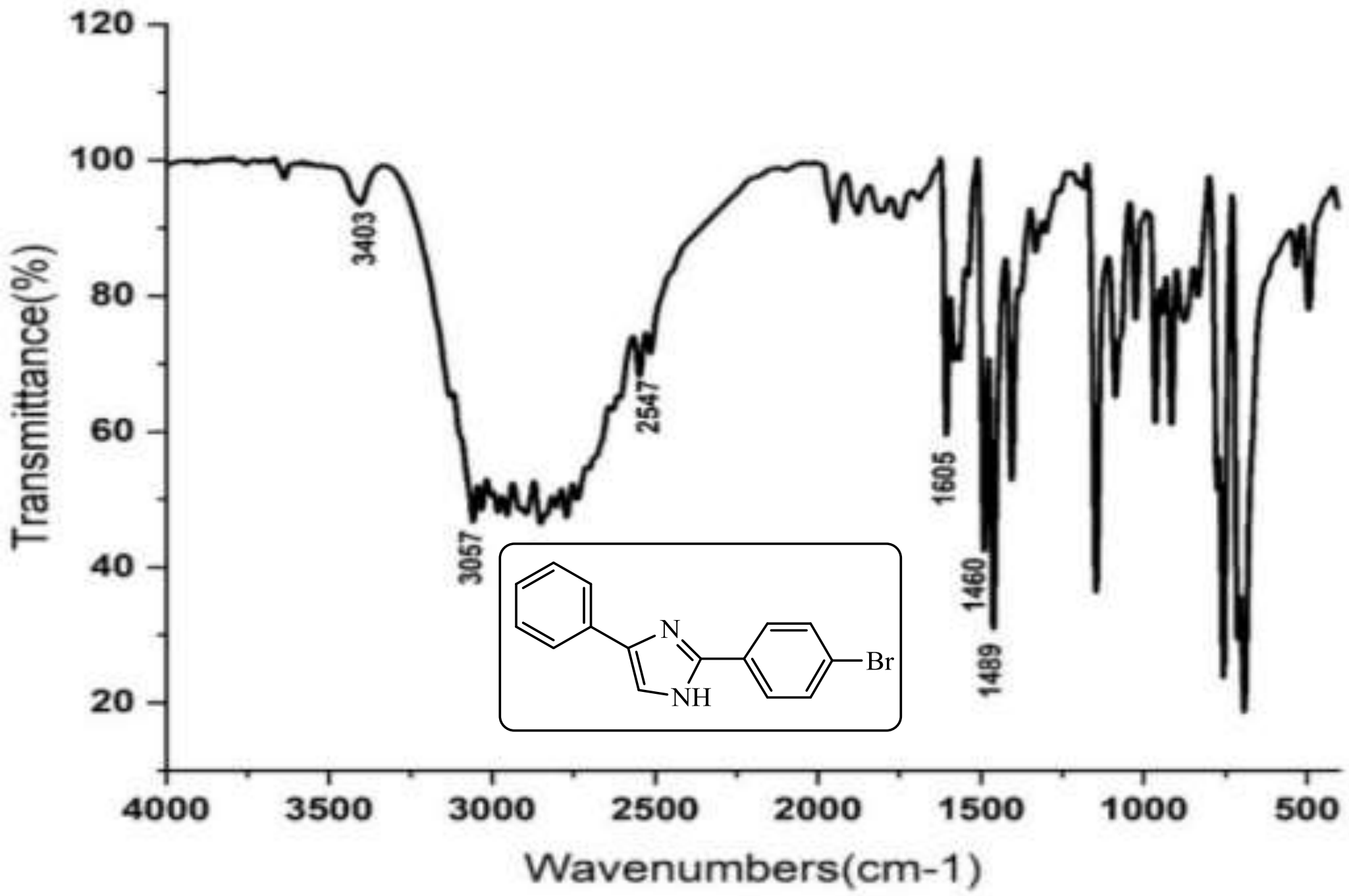


**Figure S19**: The FT-IR spectrum of compound **2-Phenyl-4(5)- (4-bromophenyl) imidazole (3g)**

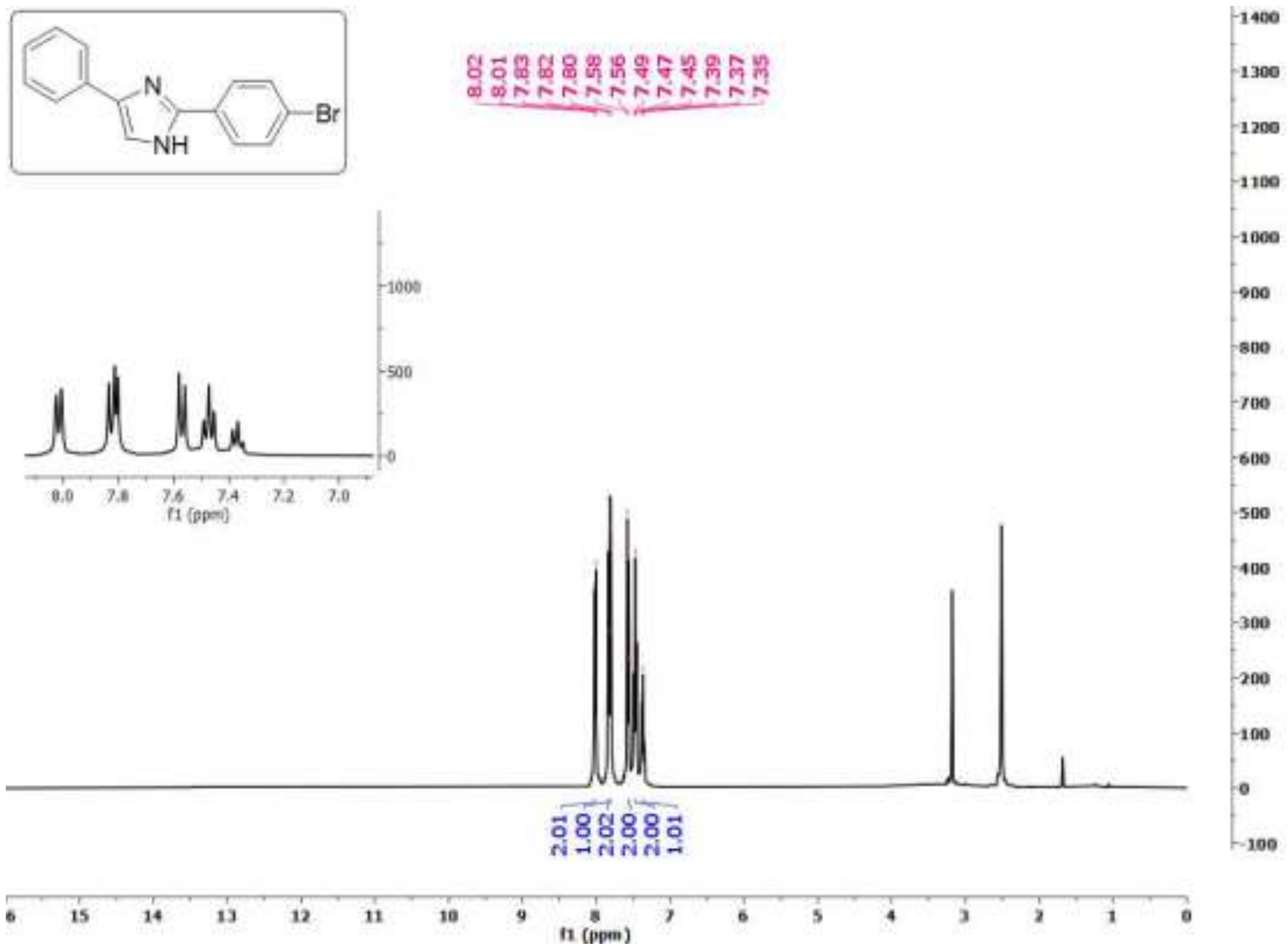


**Figure S20**: The $^{1}$H NMR spectrum of compound **2-Phenyl-4(5)- (4-bromophenyl) imidazole (3g)**

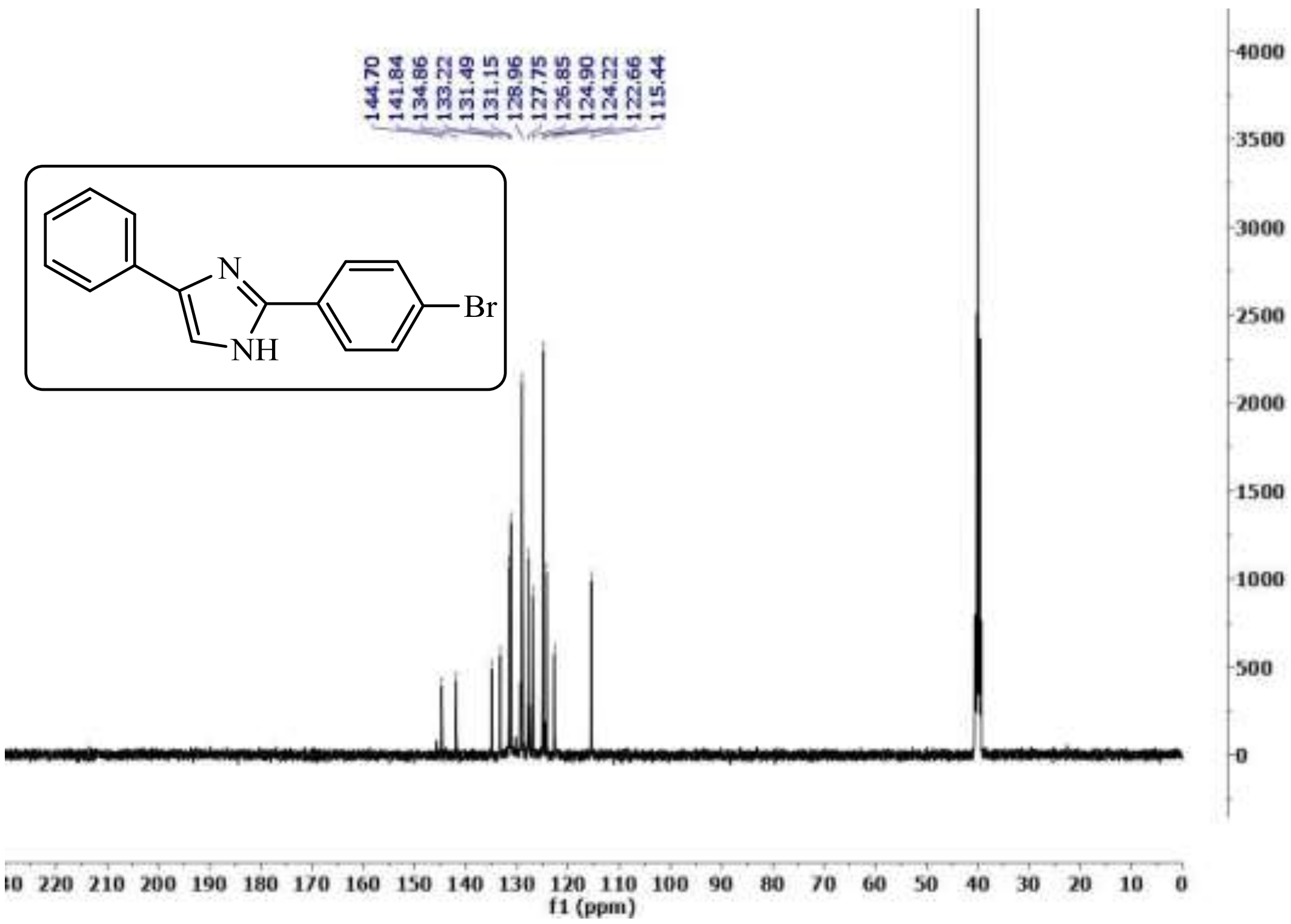


**Figure S21**: The $^{13}C$ NMR spectrum of compound **2-Phenyl-4(5)- (4-bromophenyl) imidazole (3g)**

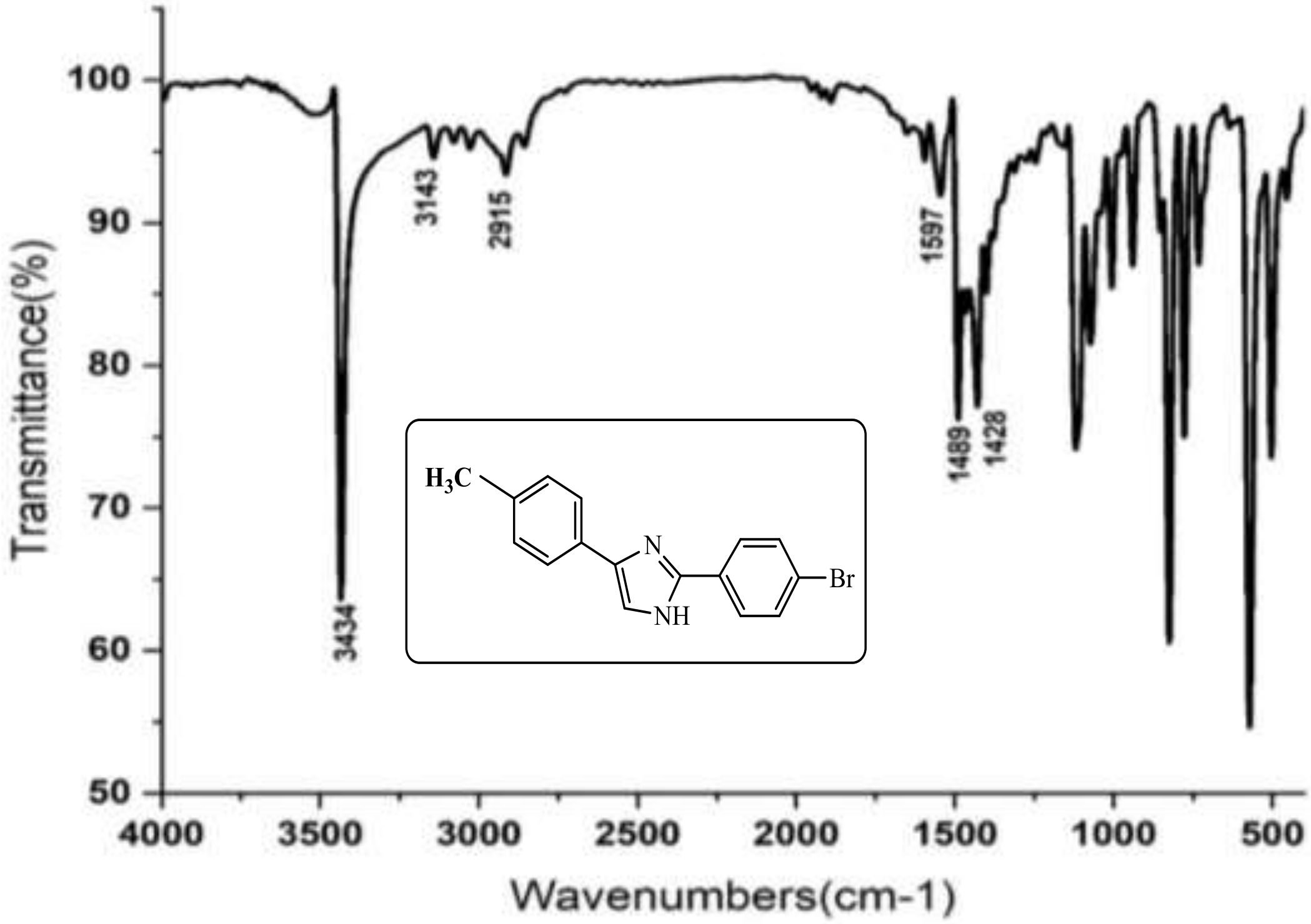


**Figure S22**: The FT-IR spectrum of compound **2-(4-methylPhenyl)-4(5)- (4-bromophenyl) imidazole (3h)**

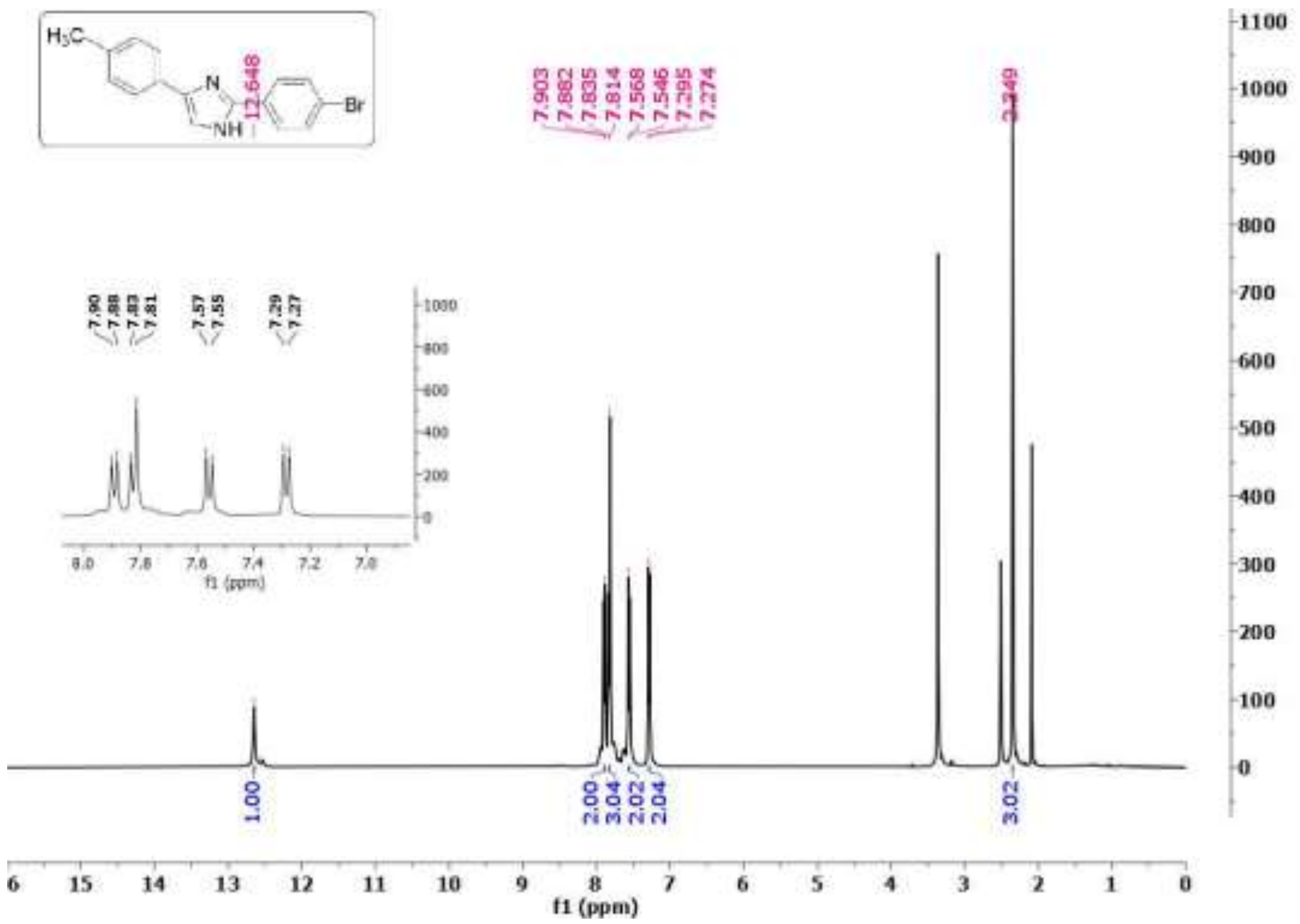

**Figure S23**: The $^{1}H$ NMR spectrum of compound **2-(4-methylPhenyl)-4(5)- (4-bromophenyl) imidazole (3h)**

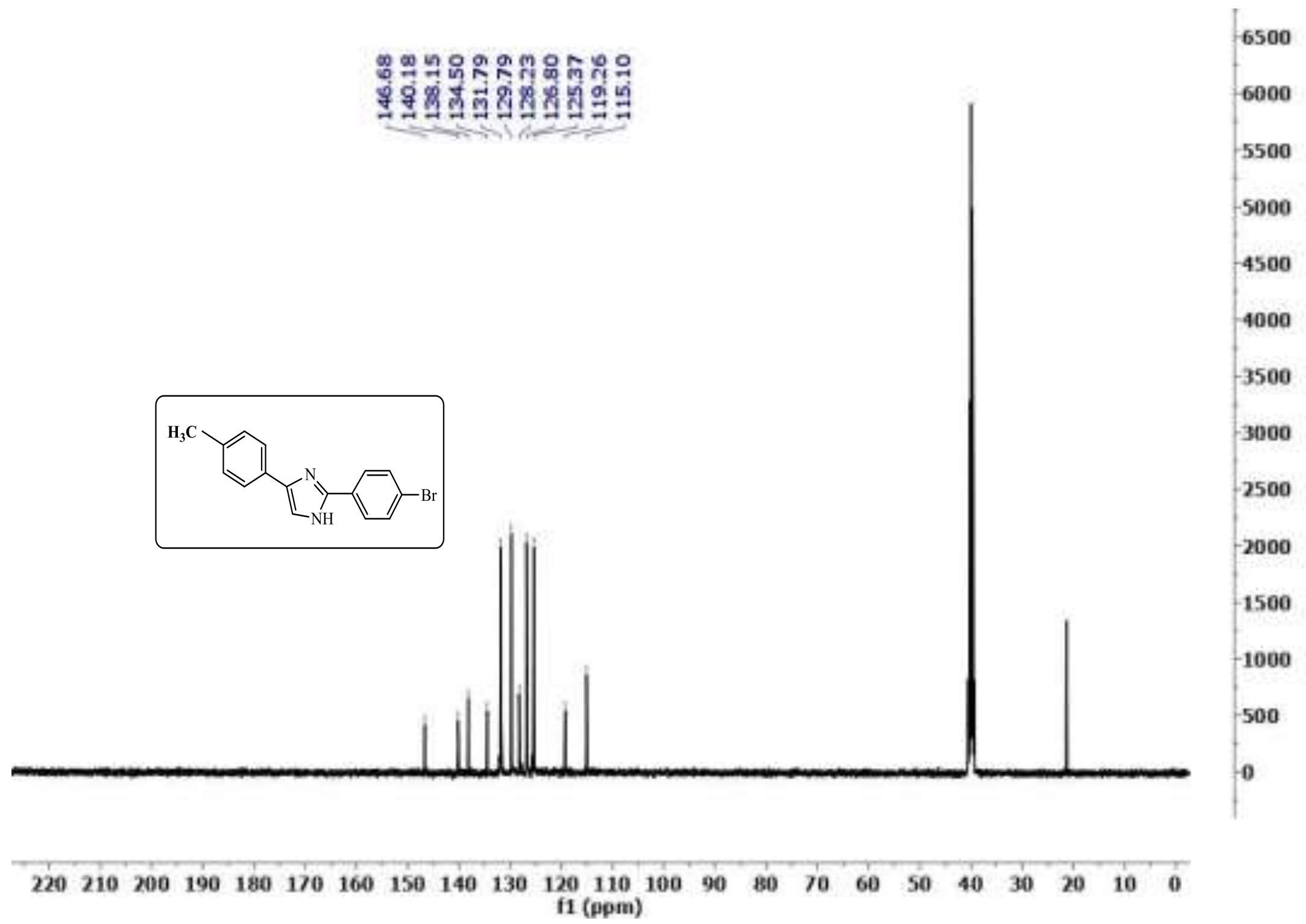

**Figure S24**: The $^{13}C$ NMR spectrum of compound **2-(4-methylPhenyl)-4(5)- (4-bromophenyl) imidazole (3h)**

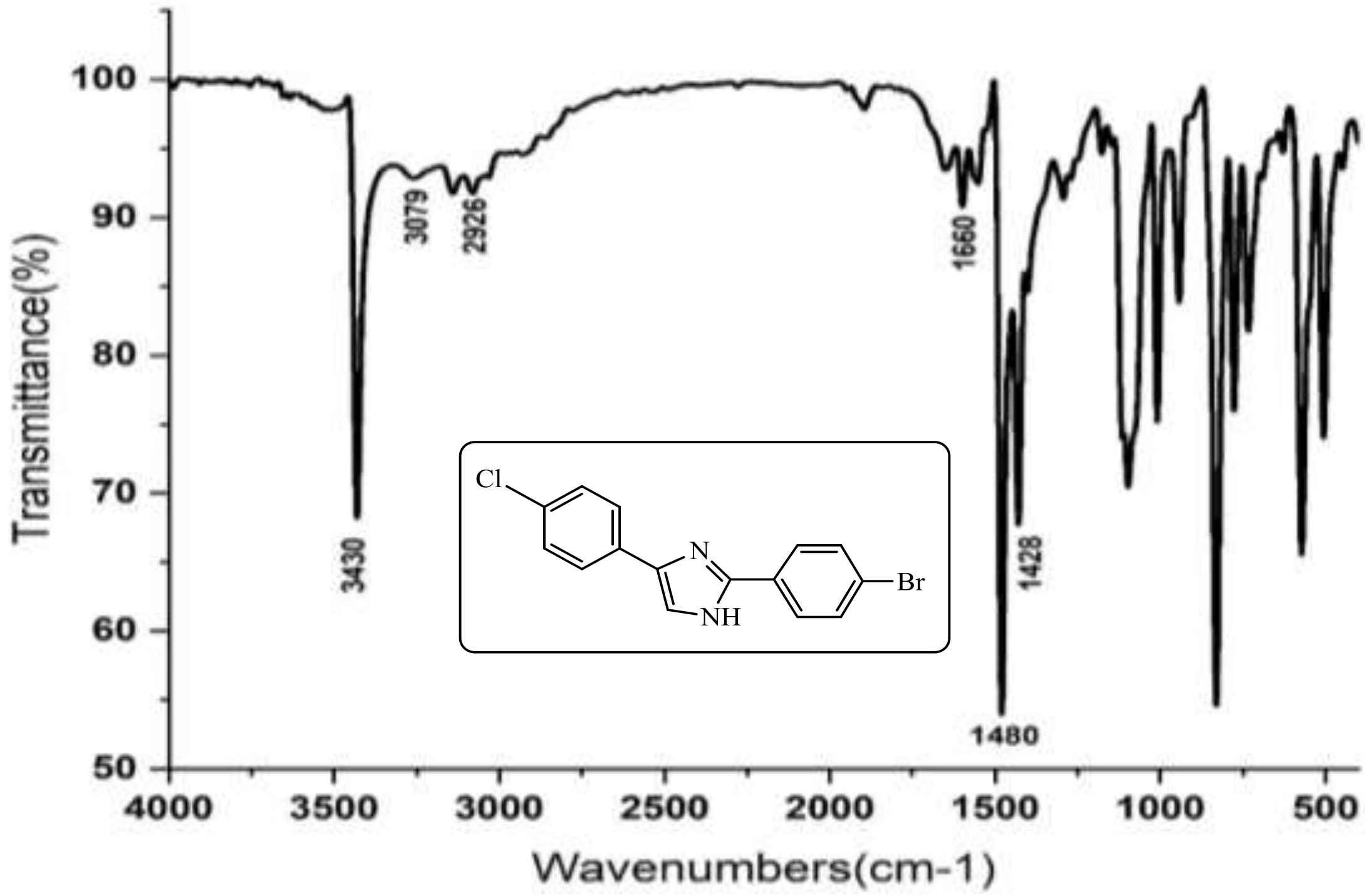


**Figure S25**: The FT-IR spectrum of compound **2-(4-ChloroPhenyl)-4(5) - (4-boromo phenyl) imidazole (3i)**

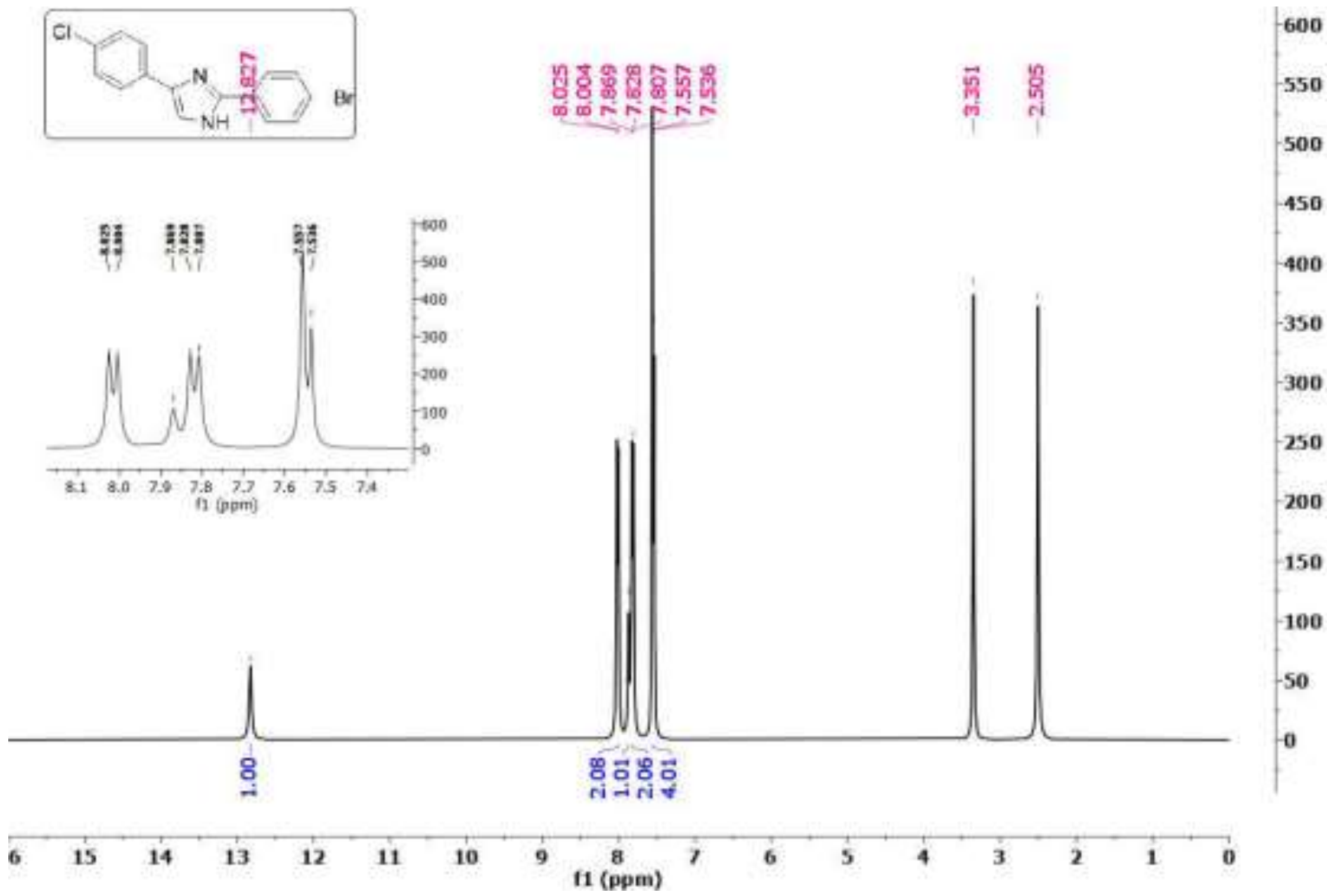


**Figure S26**: The $^{1}$H NMR spectrum of compound **2-(4-ChloroPhenyl)-4(5) - (4-boromo phenyl) imidazole (3i)**

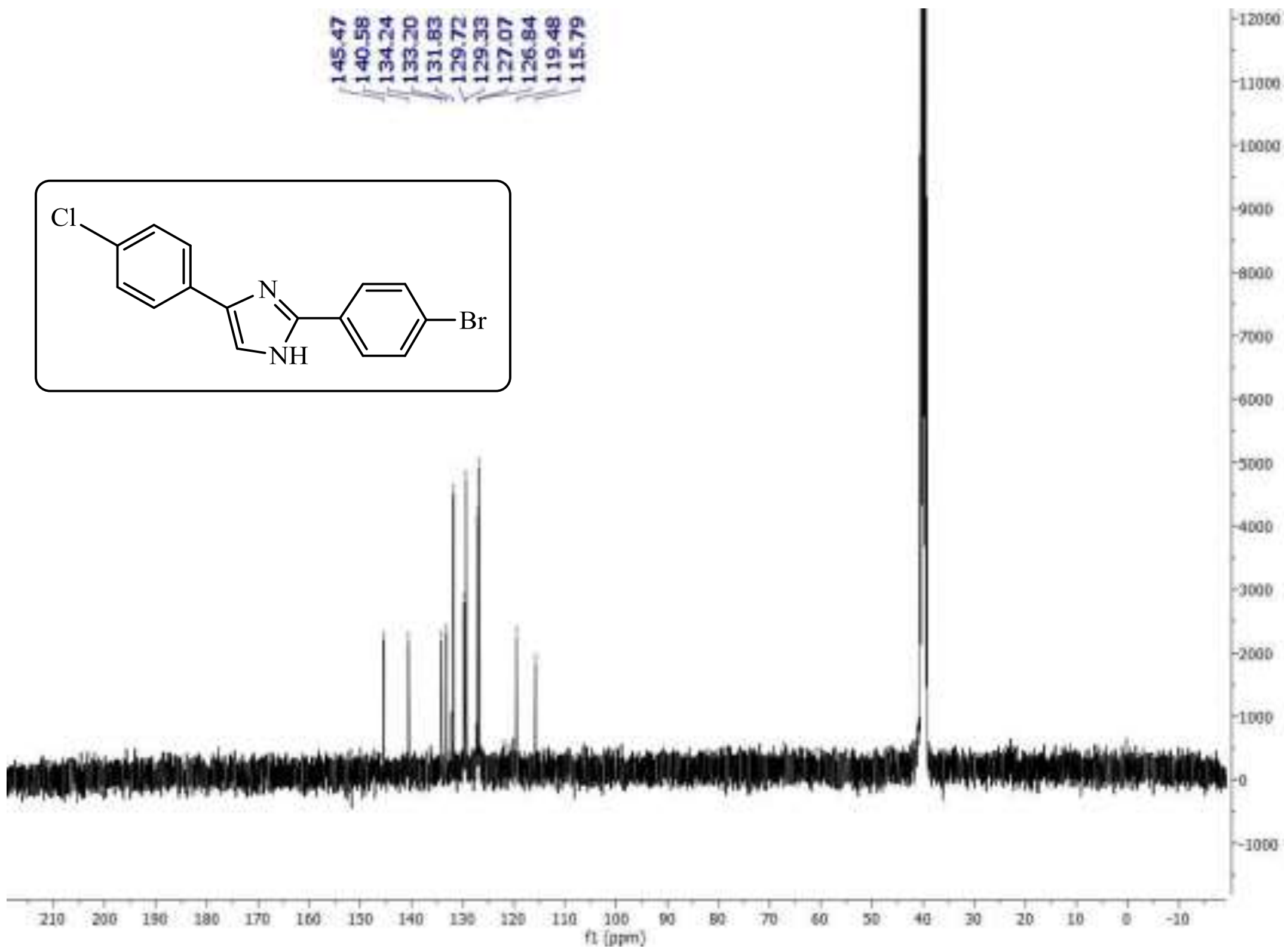


**Figure S27**: The $^{13}C$ NMR spectrum of compound **2-(4-ChloroPhenyl)-4(5) - (4-boromo phenyl) imidazole (3i)**

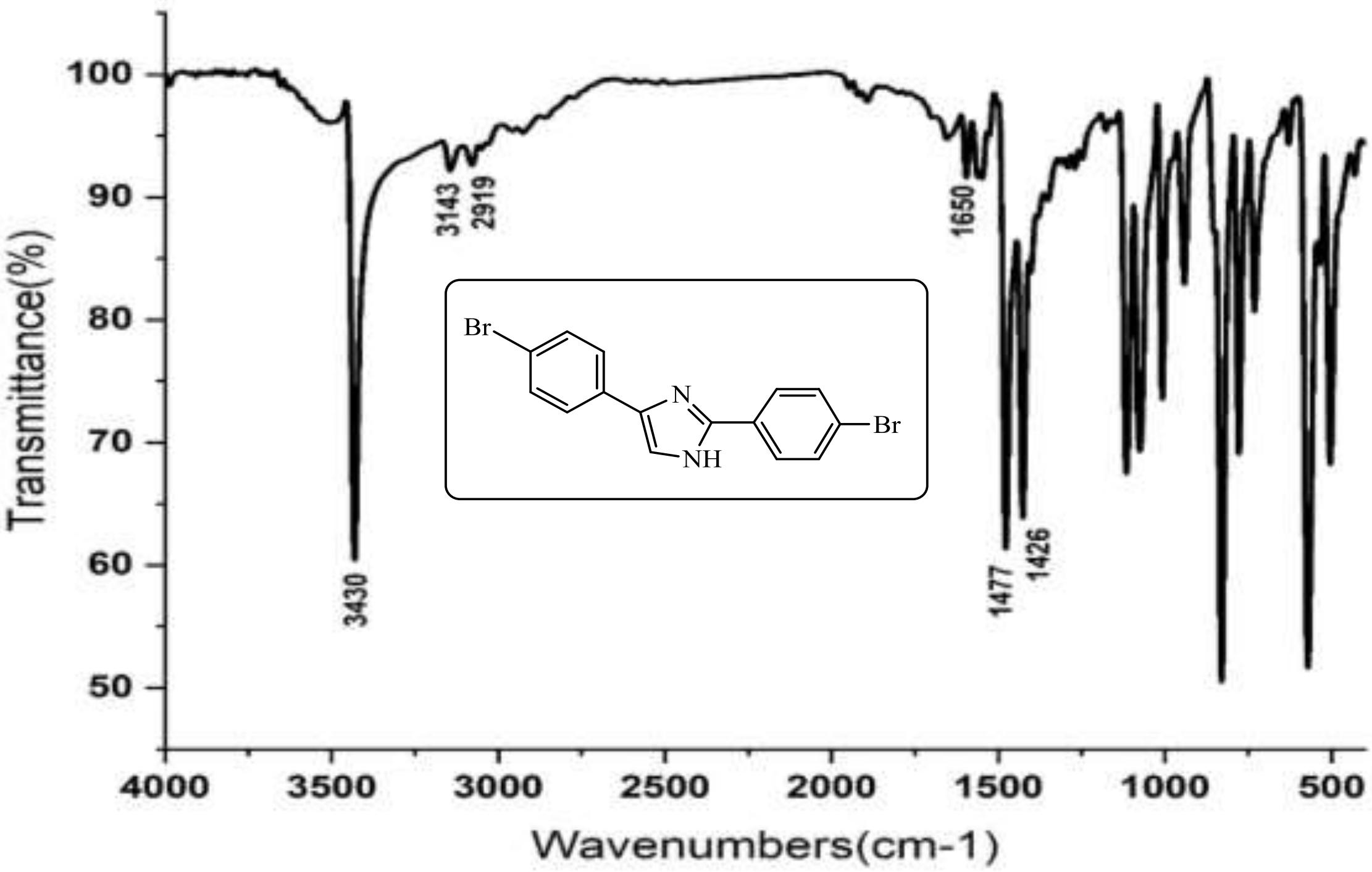


**Figure S28**: The FT-IR spectrum of compound **2-(4-BoromoPhenyl)-4(5) - (4-boromo phenyl) imidazole (3j)**

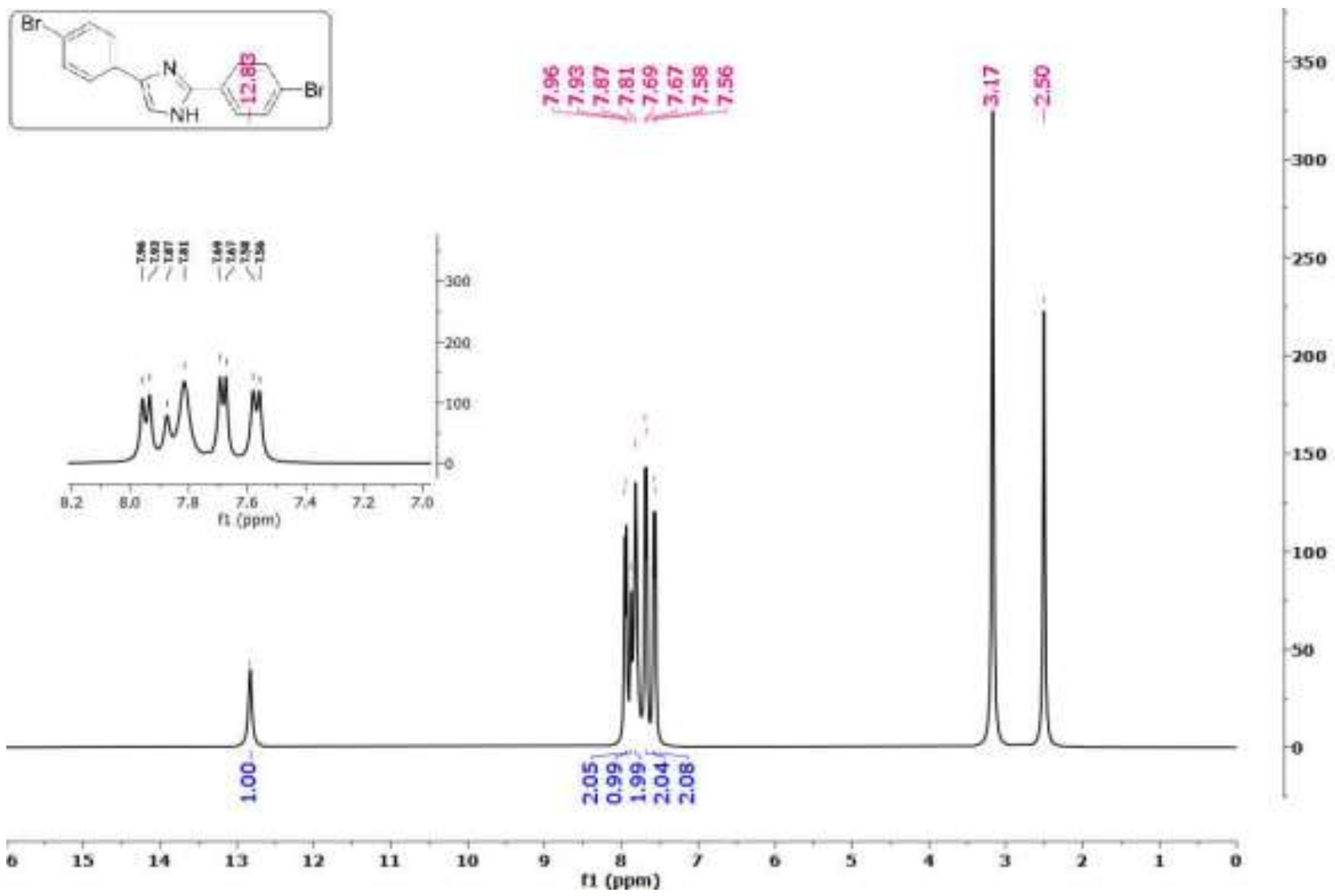


**Figure S29**: The $^{1}$H NMR spectrum of compound **2-(4-BoromoPhenyl)-4(5) - (4-boromo phenyl) imidazole (3j)**

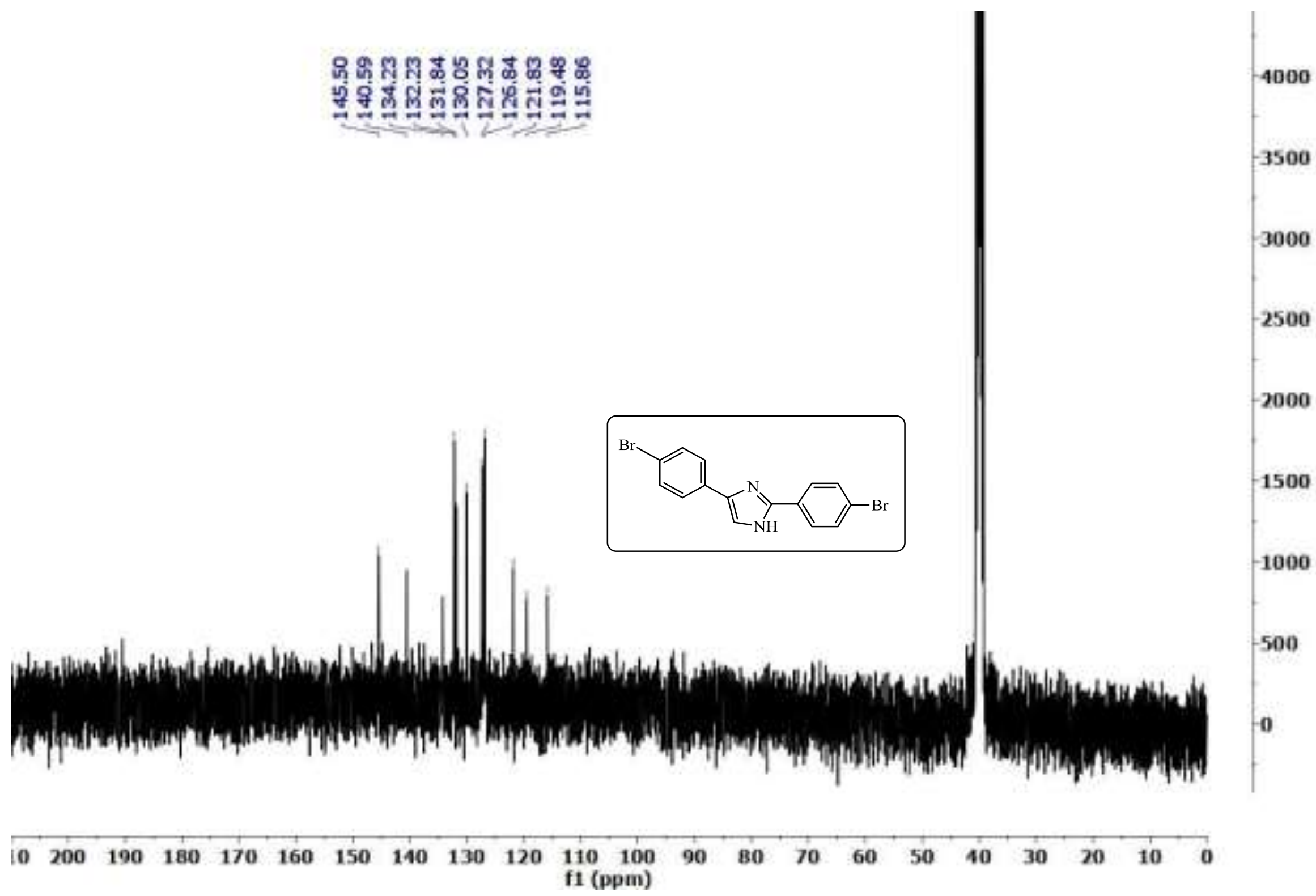


**Figure S30**: The $^{13}$C NMR spectrum of compound **2-(4-BoromoPhenyl)-4(5) - (4-boromo phenyl) imidazole (3j)**

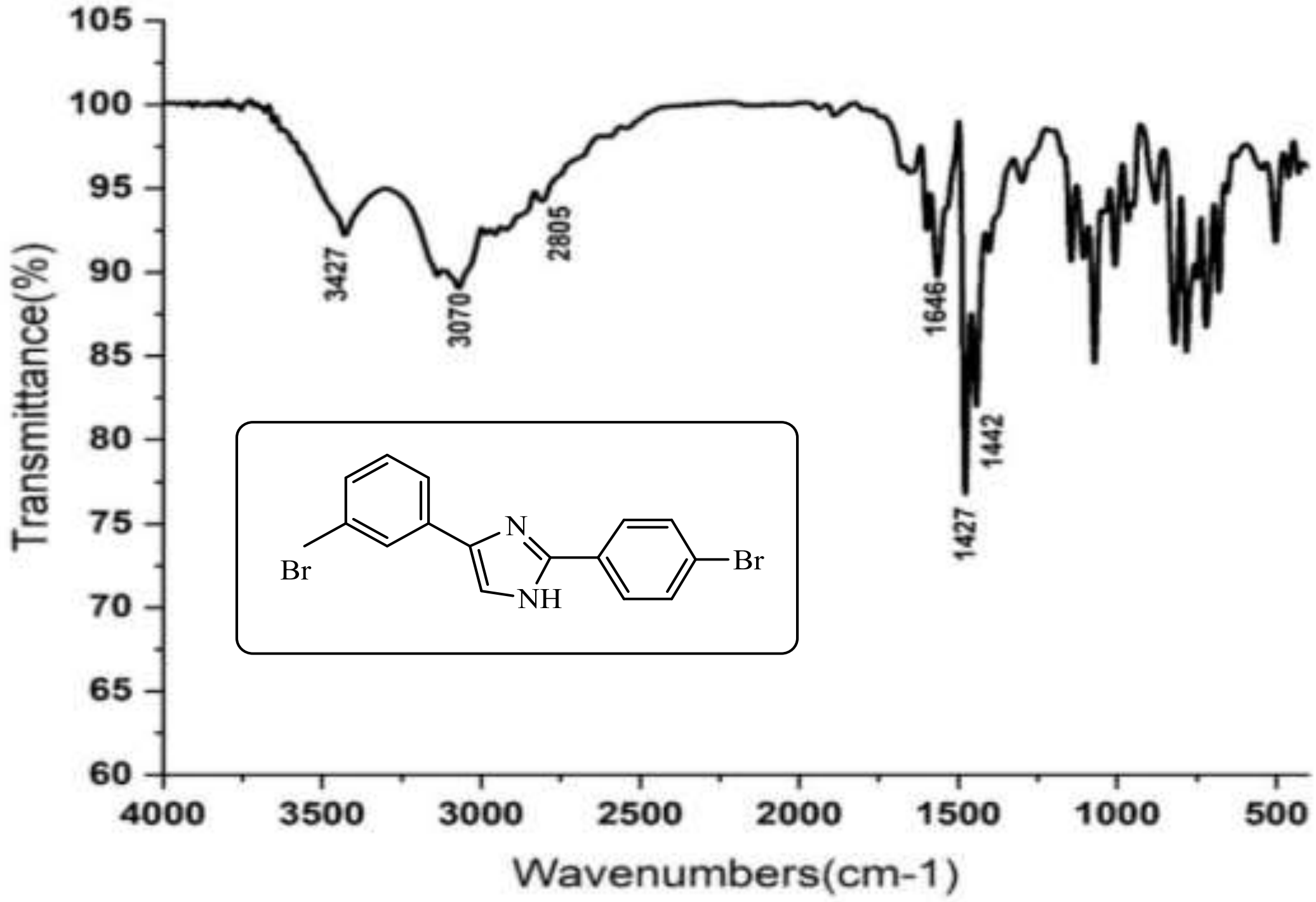


**Figure S31**: The FT-IR spectrum of compound **2-(3-BoromoPhenyl)-4(5) - (4-boromo phenyl) imidazole (3k)**

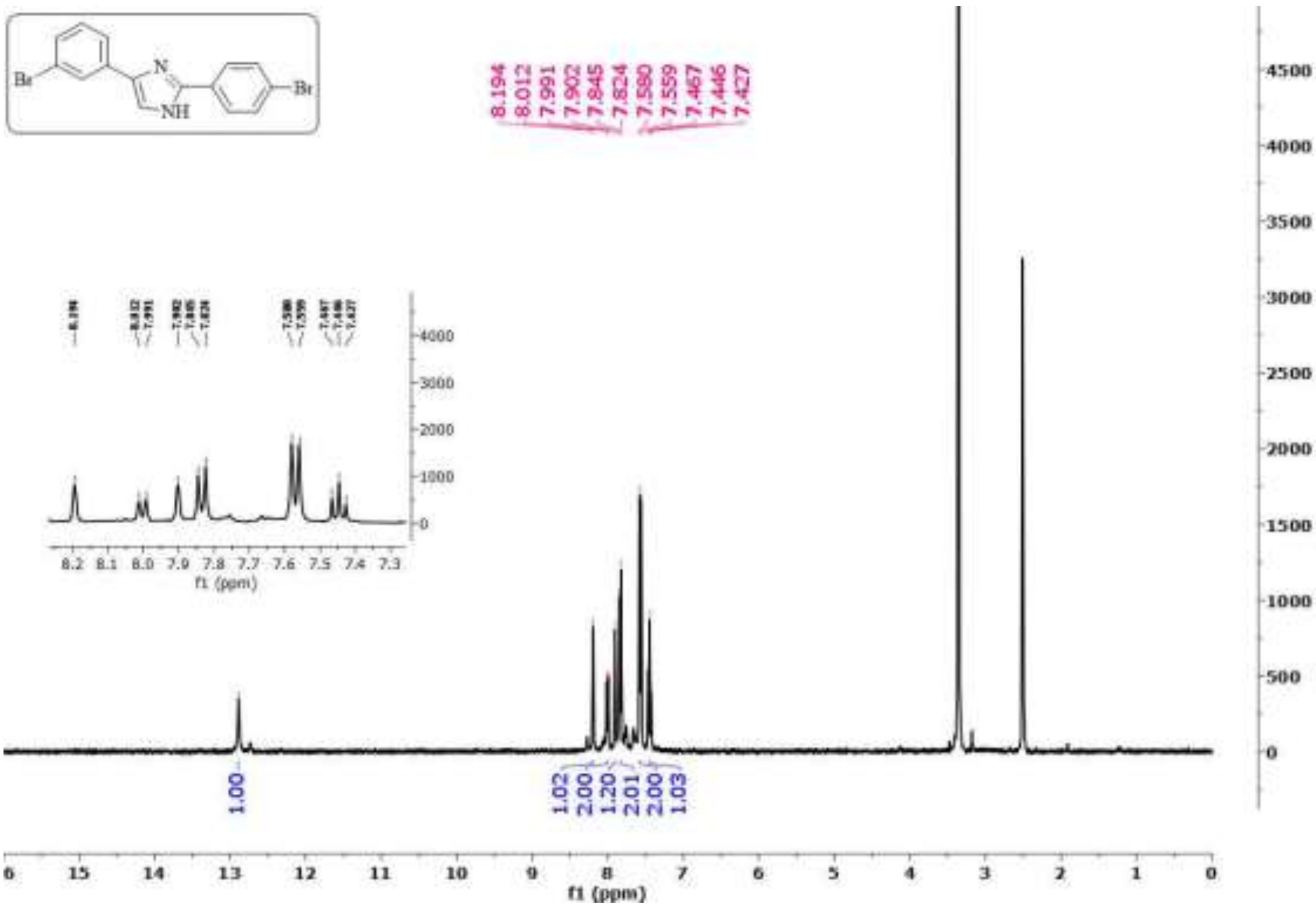


**Figure S32**: The $^{1}$H NMR spectrum of compound **2-(3-BoromoPhenyl)-4(5) - (4-boromo phenyl) imidazole (3k)**

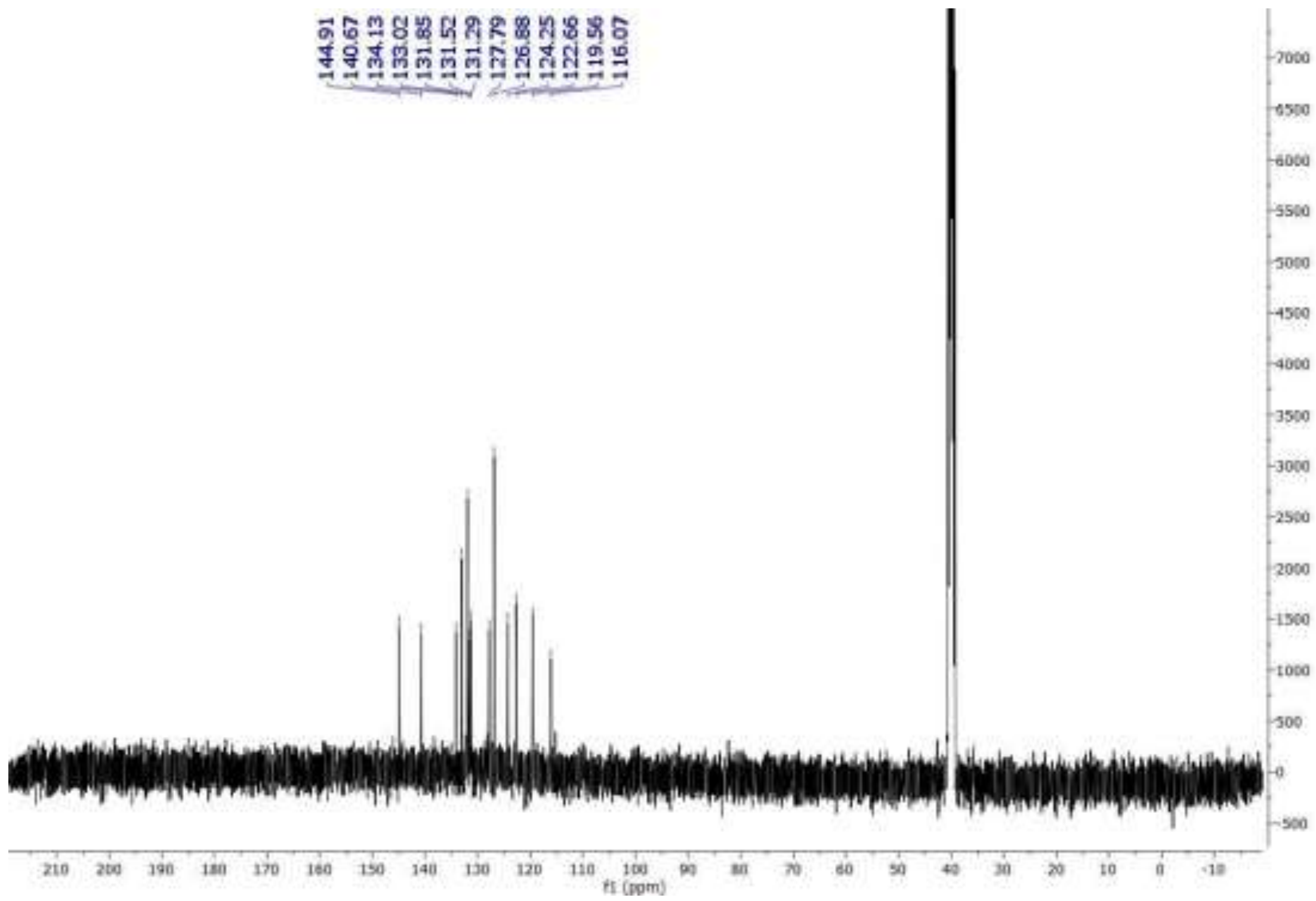


**Figure S33**: The $^{13}$C NMR spectrum of compound **2-(3-BoromoPhenyl)-4(5) - (4-boromo phenyl) imidazole (3k)**

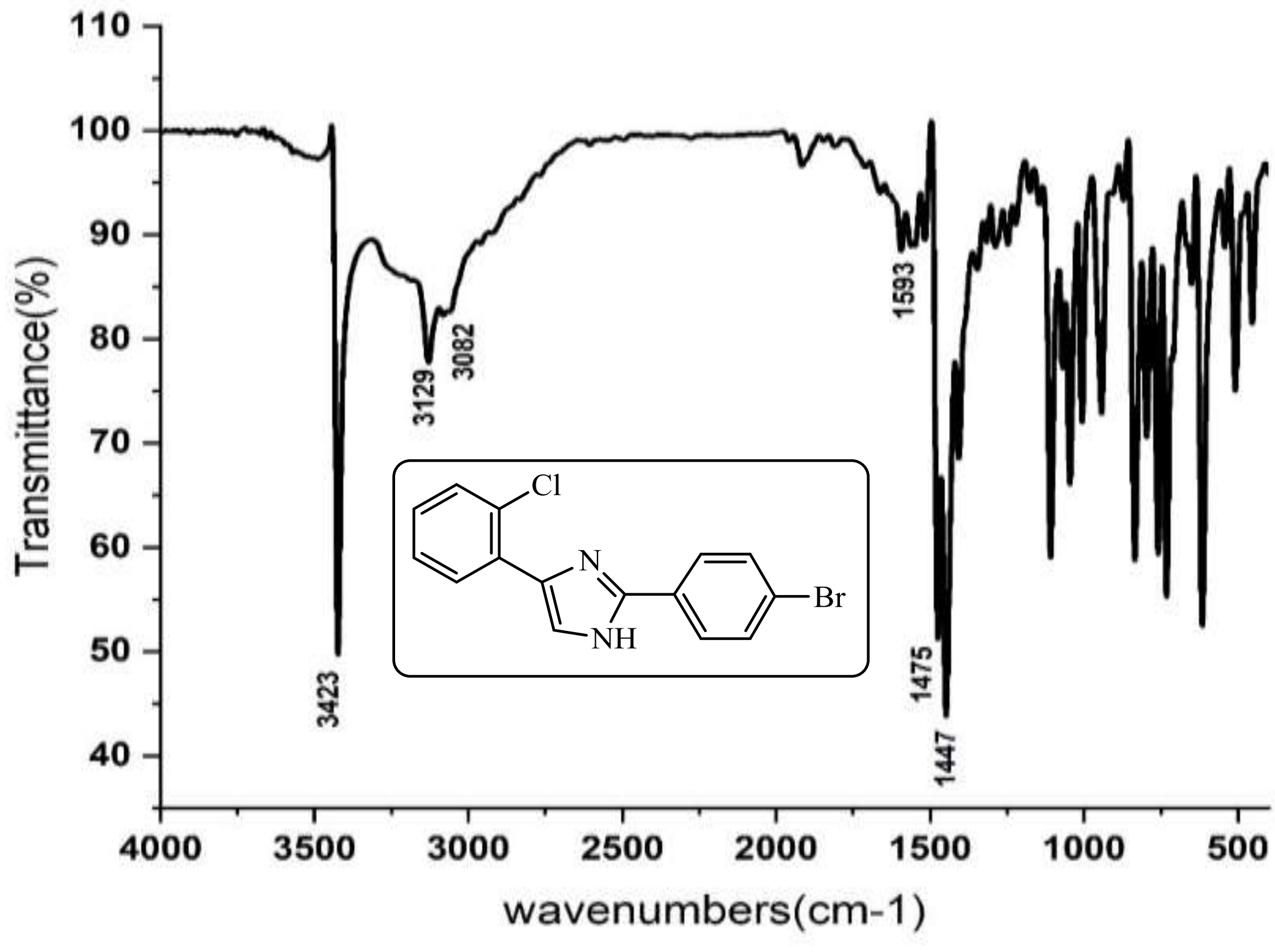


**Figure S34**: The FT-IR spectrum of compound **2-(2-ChloroPhenyl)-4(5) - (4-boromo phenyl) imidazole (3l)**

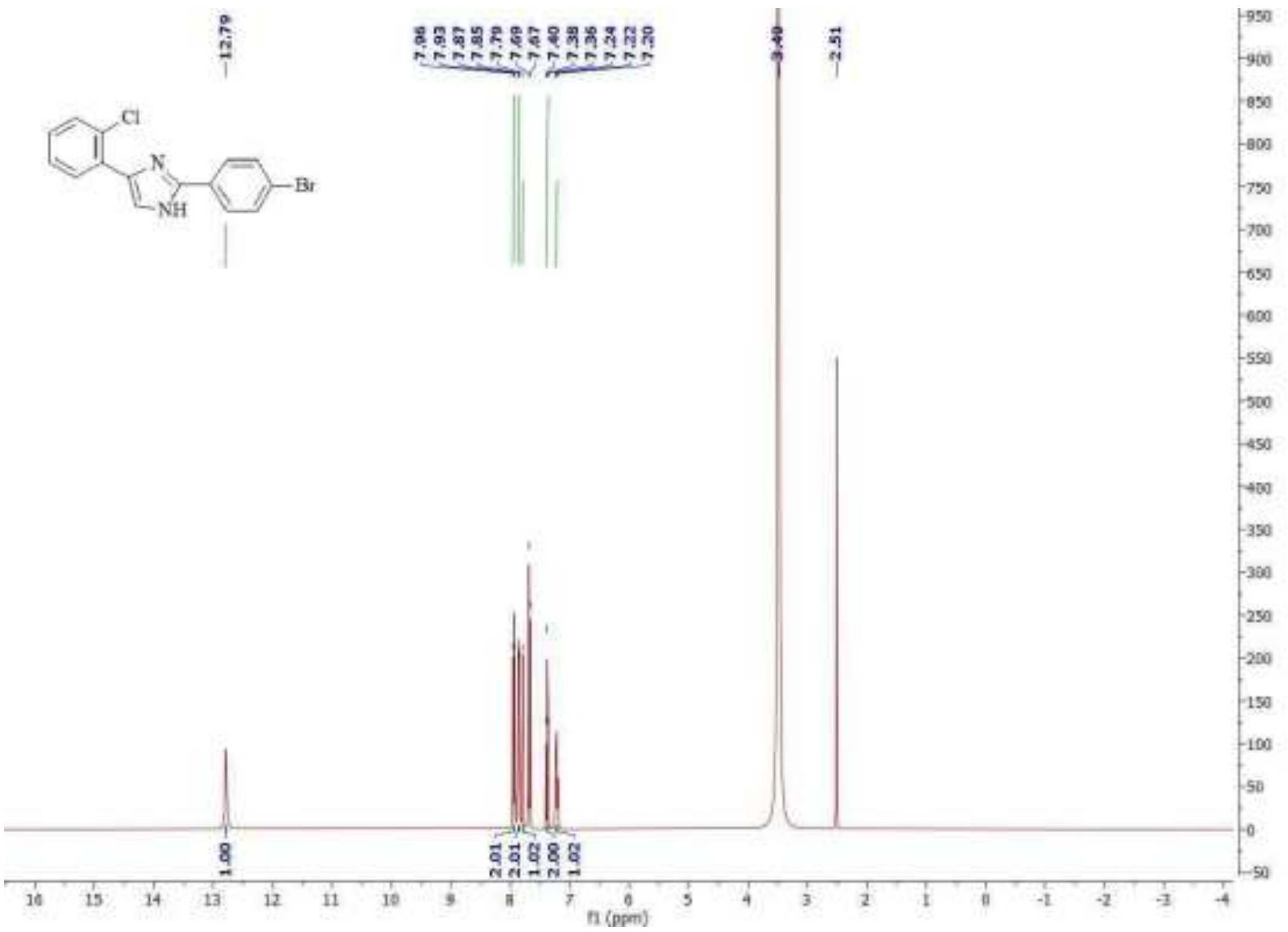

**Figure S35**: The $^{1}H$ NMR spectrum of compound **2-(2-ChloroPhenyl)-4(5) - (4-boromo phenyl) imidazole (3l)**

***Procedure for the synthesis of Phenylglyoxal derivatives***

Phenylglyoxal, one of the starting materials in the synthesis reaction, was synthesized and used after characterization and confirmation by $^{1}H$ NMR. The $^{1}H$ NMR spectrum acquired in $CDCl_3$ displays a peak corresponding to the phenylglyoxal aldehyde group at 9.70 ppm, confirming the presence of the dicarbonyl.

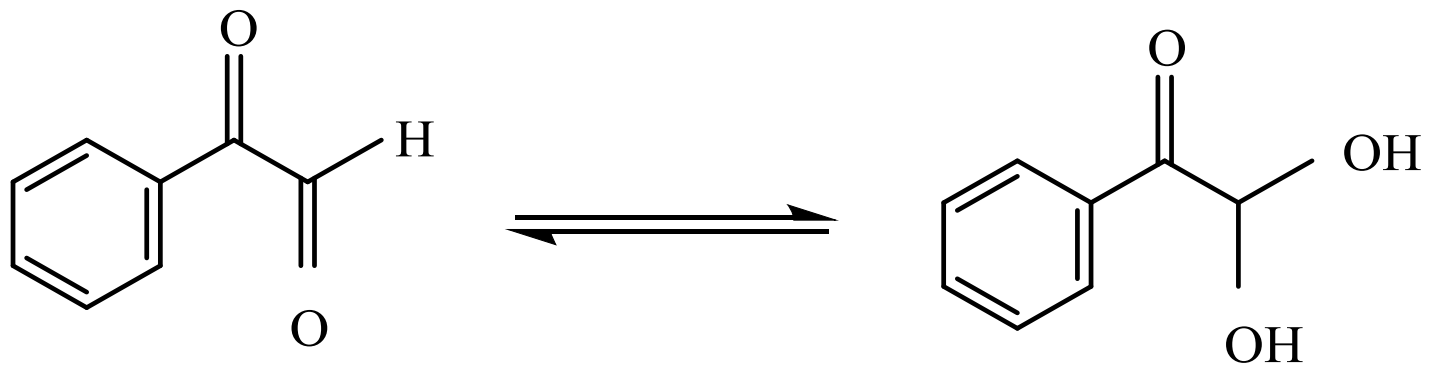

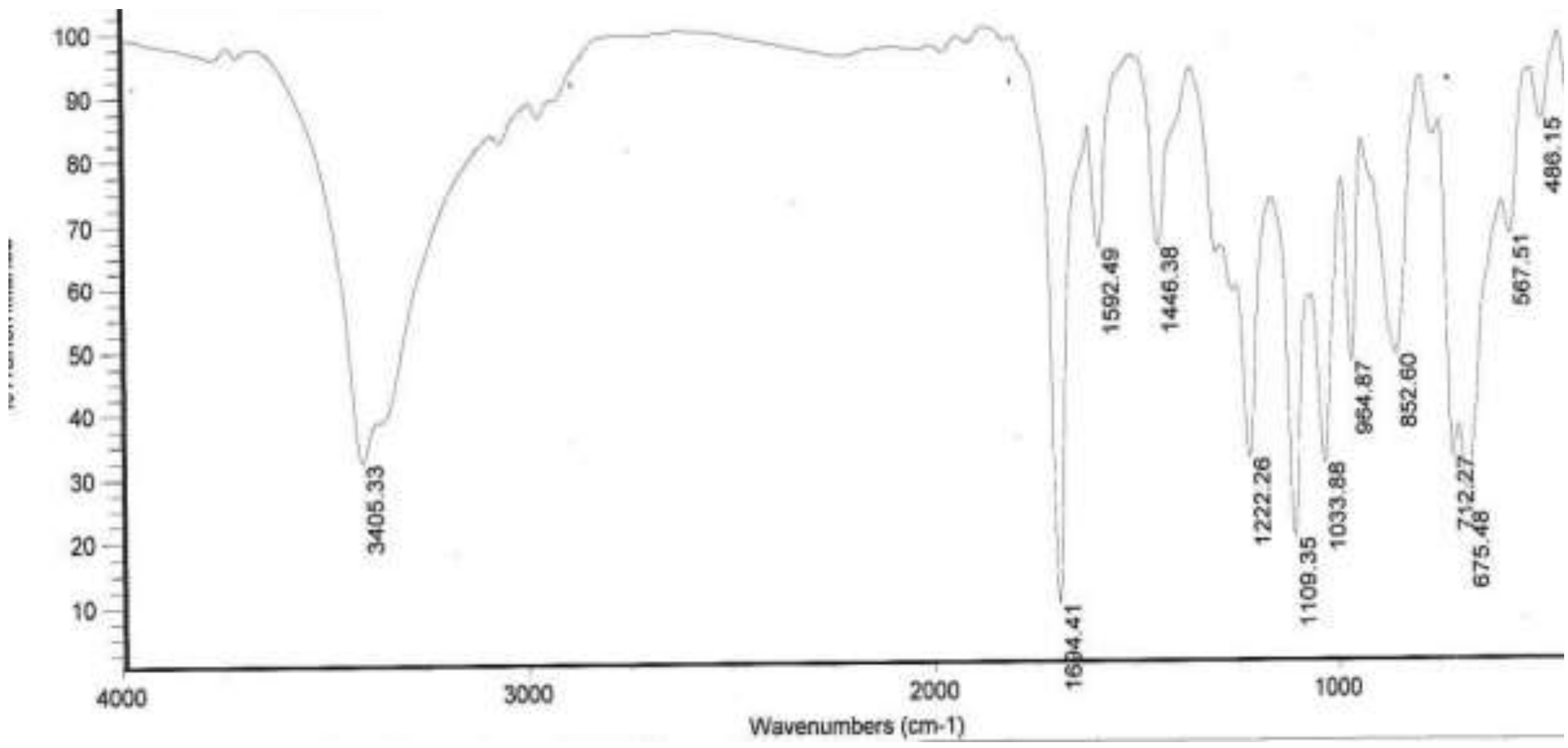


**Figure S36**: The FT-IR spectrum of Phenylglyoxal

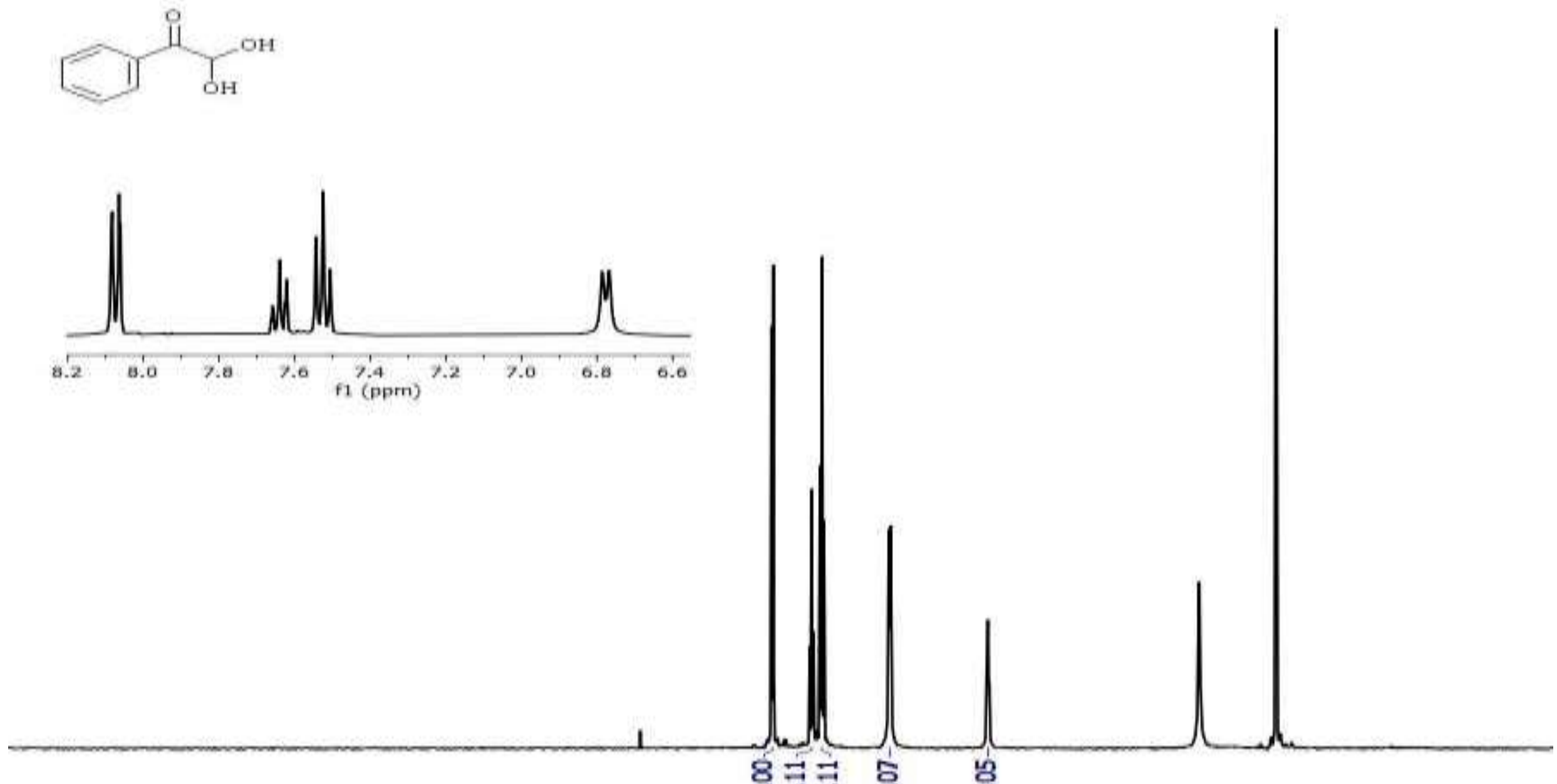


**Figure S37**: The $^{1}H$ NMR spectrum of Phenylglyoxal
